# Clinical Genomic Sequencing Reports in Electronic Health Record Systems Based on International Standards: Implementation Study

**DOI:** 10.2196/15040

**Published:** 2020-08-10

**Authors:** Borim Ryu, Soo-Yong Shin, Rong-Min Baek, Jeong-Whun Kim, Eunyoung Heo, Inchul Kang, Joshua SungWoo Yang, Sooyoung Yoo

**Affiliations:** 1 Office of eHealth and Business Seoul National University Bundang Hospital Seongnam Republic of Korea; 2 Department of Digital Health Samsung Advanced Institute for Health Sciences and Technology Sungkyunkwan University Seoul Republic of Korea; 3 Big Data Research Center Samsung Medical Center Seoul Republic of Korea; 4 Department of Plastic and Reconstructive Surgery Seoul National University Bundang Hospital Seongnam Republic of Korea; 5 Department of Otorhinolaryngology Seoul National University Bundang Hospital Seongnam Republic of Korea; 6 Research and Development Center ezCaretech Seoul Republic of Korea; 7 Big Data Center Macrogen Seoul Republic of Korea

**Keywords:** standardization, genomics, electronic health record, information system, data exchange

## Abstract

**Background:**

To implement standardized machine-processable clinical sequencing reports in an electronic health record (EHR) system, the International Organization for Standardization Technical Specification (ISO/TS) 20428 international standard was proposed for a structured template. However, there are no standard implementation guidelines for data items from the proposed standard at the clinical site and no guidelines or references for implementing gene sequencing data results for clinical use. This is a significant challenge for implementation and application of these standards at individual sites.

**Objective:**

This study examines the field utilization of genetic test reports by designing the Health Level 7 (HL7) Fast Healthcare Interoperability Resources (FHIR) for genomic data elements based on the ISO/TS 20428 standard published as the standard for genomic test reports. The goal of this pilot is to facilitate the reporting and viewing of genomic data for clinical applications. FHIR Genomics resources predominantly focus on transmitting or representing sequencing data, which is of less clinical value.

**Methods:**

In this study, we describe the practical implementation of ISO/TS 20428 using HL7 FHIR Genomics implementation guidance to efficiently deliver the required genomic sequencing results to clinicians through an EHR system.

**Results:**

We successfully administered a structured genomic sequencing report in a tertiary hospital in Korea based on international standards. In total, 90 FHIR resources were used. Among 41 resources for the required fields, 26 were reused and 15 were extended. For the optional fields, 28 were reused and 21 were extended.

**Conclusions:**

To share and apply genomic sequencing data in both clinical practice and translational research, it is essential to identify the applicability of the standard-based information system in a practical setting. This prototyping work shows that reporting data from clinical genomics sequencing can be effectively implemented into an EHR system using the existing ISO/TS 20428 standard and FHIR resources.

## Introduction

In order to realize the potential of personalized precision medicine through genetic testing, the results of genetic testing must be integrated with other clinical information to improve patient care. Because the electronic health records (EHRs) of today seem ill suited for managing genomic data, other solutions are required, including universal data standards and applications that use application programming interfaces (APIs), along with a commitment on the part of sequencing laboratories to consistently provide structured genomic data for clinical use [1]. In addition, a clinical interface is needed for genetic test reporting that provides genetic evidence as support for clinical decision making. Many standards for genetic test reporting have been presented so far, but they often lack guidance for specific data items and realistic implementation guidelines. Tarczy-Hornoch et al reported that the parallel efforts across clinical sequencing exploratory research sites in the creation of systems for report generation and integration of reports into the EHR system, as well as the lack of standardized approaches to interfacing with variant databases to create active clinical decision support, create opportunities for cross-site and vendor collaborations [2-4]. This has prevented clinical implementation of these standards. Nonetheless, these standards offer a potential foundation for the creation of clinically relevant standards. ISO/TC 215 is the International Organization for Standardization's (ISO) Technical Committee (TC) on health informatics. TC 215 works on the standardization of health information and communications technology (ICT) to ensure compatibility and interoperability between independent systems. ISO also published two genomics standards [5,6] to exchange genomic sequence variation in XML format and to incorporate a structured clinical sequencing report to EHRs. In addition, many other genomics standards for whole genome sequencing, gene fusion reports, and omics data are now under development [7-11]. ISO 25720:2009 is a standard that defines how gene sequence variation information is presented in an XML-based data exchange format [5,12]. This standard enables the transmission and reception of genetic variation information around the world. The scope of ISO 25720:2009 is the data exchange format, but notably it does not include a database schema. From a biological point of view, single nucleotide polymorphisms are the main target of this standard. ISO/TS (Technical Specification) 22220:2011 defines data elements and structures in the medical sector to ensure accurate personal identification through calculation [13]. This standard also defines statistical and other identification data elements to facilitate the improved identification of patients at medical institutions. Finally, ISO/TS 20428 is a standard for defining structured techniques so that genetic screening reports can be effectively communicated and utilized by the clinical workforce [6]. The standard is called *Health informatics— Data elements and their metadata for describing structured clinical genomic sequence information in electronic health records*, which proposes standard formatting for the results of next generation sequencing (NGS) genetic testing of patient samples in electronic medical records. These standards are being employed by various domestic companies and hospitals. For example, a recent study by Park et al showed the implementation of ISO/TS 20428 standard and developed a clinical research information system to integrate patient NGS data with clinical data [14].

Despite these standards, most hospitals in Korea produce genetic test reports in text or PDF format and provide them to medical staff. Furthermore, detailed examination results are managed in different formats by different agencies. To overcome these discrepancies, standards for utilizing genetic information at medical sites should be developed, and an analysis pipeline should be created where data can be freely processed in the form that meets the needs of the site. Of note, it is difficult to apply the current standards to actual sites because the previously proposed ISO/TS 20428 standard contains only definitions and concepts of data items. Significantly, it does not provide the practical guidance needed for actual development and implementation, nor does it provide guidelines for integrating this data with clinical records. As such, it is beneficial to investigate existing data from hospital systems that have implemented these standards. This study examined the field utilization of genetic test report data by designing the Fast Healthcare Interoperability Resources (FHIR) Genomics for Health Level 7 (HL7) for data elements based on the ISO/TS 20428 standard for genomic test reports. This study aims to verify the actual implementation of the ISO/TS 20428 standard in clinical settings and to report an approach for the implementation of a structured clinical sequencing report in EHRs according to international standards. We demonstrated the feasibility of implementing the ISO/TS 20428 standard by studying its application at various sites and integrating the clinical sequencing reports into the EHR system of a tertiary university hospital according to the HL7 FHIR standard.

## Methods

### International Standards for Clinical Sequencing Reports in EHRs

ISO/TS 20428:2017, entitled *Health informatics – Data elements and their metadata for describing structured clinical genomic sequence information in electronic health records*, defines the data elements and their metadata required for a structured clinical genomic sequencing report [6]. ISO/TS 20428 focuses on genomic data generated using NGS technologies from only human samples. The data elements are divided into data fields required for clinical applications and optional data fields for clinical research, such as clinical trials and translational research. The metadata explains how and where particular appropriate terminological systems, which describe the genomes and/or diseases, can be applied. Thus, this standard attempts to integrate clinical and genomic information for a structured clinical genomic sequencing report template into an EHR system.

HL7 FHIR is an emerging standard that is rapidly gaining attention and being adopted by the health information technology industry and health care organizations [15]. This standard implements various types of information generated in the clinical environment in the form of a *resource* for exchanging clinical data and, thus, it ensures interoperability. Resources are an instance-level representation of a certain type of health care entity. Tailoring the FHIR specification to a specific use case is an important aspect of implementing the FHIR standard. This process, also called profiling, enables FHIR to be adjusted to the needs of users and defines how exchanging organizations use the FHIR specification. FHIR profiles, as conformance resources, are an important aspect of the FHIR standard. Among the diverse FHIR profiles, FHIR Genomics has also been developed. FHIR Genomics comprises the MolecularSequence resource and several profiles built on top of existing FHIR resources, including a DiagnosticReport-genetics profile on DiagnosticReport, a ServiceRequest-genetics profile on ServiceRequest, an Observation-genetics profile on Observation, and a human leukocyte antigen (HLA)-genotyping-results profile on DiagnosticReport [16]. The MolecularSequence resource is designed for NGS data. The observed sequences of patients should be represented by recording reference sequence IDs and strings as well as detected variants. Extensions of the MolecularSequence resource address complex cases, and they can associate these with repositories for retrieving a patient’s full sequence data. Other modifications include a set of genetics profiles for other FHIR resources. In addition, the Observation-genetics profile adds new references such that the Observation can report the genetics test results to be integrated into the EHR. The Observation-genetics profile is used to interpret variants from the sequence resource. Clinical usage may need more specific representation of variants at the locus or structural variants in the entire genome. This DiagnosticReport-genetics is designed based on the DiagnosticReport. The new profile is then used to describe the genetics test report. The resulting element in the DiagnosticReport refers to the Observation resource that can lead to a bundle of genetic observations. The elements of code, effective[x], issued, performer, request, and specimen are used to describe the details of the genetic test. Extensions of AssessedCondition and FamilyMemberHistory are also added. Overall, this profile extends the DiagnosticReport resource to enable the reporting of structured genetic test results. In addition, it denotes the condition context for genetic testing, which may influence the reported variants and interpretations for large genomic testing panels. Further, there are new genetics-extension profiles for extending DiagnosticReport, ServiceRequest, and FamilyMemberHistory for reporting genetics results. We have provided the suffix “-genetics” to all the aforementioned FHIR genetics profiles (eg, “DiagnosticReport-genetics profile”). New profiles built upon the DiagnosticReport have been created for reporting HLA genotyping results. FHIR Genomics focuses on clinical genetic data reporting and covers many aspects of genetic reporting, which includes bacterial and viral specimens, representations of simple discrete variants and structural variants, and full or partial DNA sequencing. In this study, existing FHIR Genomics resources were used, and additional extensions were further defined to expose gene variant data for presentation to the end user.

### ISO/TS 20428 Standard Implementation Using FHIR

FHIR is a standard that focuses on providing a secure interoperability environment for health service providers to exchange data using the Representational State Transfer (REST) API [15,17,18]. We built a repository with the FHIR server that applied the STU3 (Standard Trial Use 3) version of FHIR to provide *create*, *read*, and *delete* operations for resources (Observation, Sequence) containing genome analysis result information. The build environment consisted of C# (.NET 4.5), Windows Server 2012, Oracle Database, etc. The Seoul National University Bundang Hospital (SNUBH) FHIR server supported the receiving and processing of the following sequence resource and genetics profiles operations: create, history, read, search, and update. To apply the ISO/TS 20428 standard to clinical sequencing reports within the FHIR infrastructure and to investigate its clinical applicability, profile tooling is an essential process. Therefore, we conducted a series of five collaborative workshops to compare the ISO/TS 20428 reporting standard and FHIR Genomics resources to better understand the comprehensive data requirements to define a mapping and create additional extensions. A total of 10 researchers participated in these workshops: one clinician, one nurse, five bioinformatics specialists, and three FHIR developers. The clinician—an otolaryngologist—reviewed the data items required for a clinician interface as the project supervisor. The nurse had 10 years of experience working in the medical informatics field and helped to identify the location, type, and structure of the data stored in EHRs and provided guidance. The bioinformatics specialists in this workshop had a variety of backgrounds: licensed HL7 standard professional, ISO/TS 20428 standard author, experts in medical informatics with biological backgrounds, and a PhD in genomics and systems biology. These experts were familiar with the content of ISO/TS 20428 and HL7 FHIR standards. They examined how real genomic data can be expressed and transmitted for the data elements presented in each standard. The three FHIR developers worked to implement the new standards in compliance with the security requirements for HL7 FHIR, facilitated further development for scalability, and enabled interagency data transmission.

The aim of these workshops was to develop a method for implementing the ISO/TS 20428 standard using HL7 FHIR. The opinions of all participants were collected for internal discussions; subsequently, a mapping table between the ISO/TS 20428 standard and the FHIR Genomics resources for implementing the ISO/TS 20428 standard using HL7 FHIR was constructed. All members of the workshop reviewed the ISO/TS 20428 standard and FHIR resources; they chose suitable FHIR profiles to implement the standard in the form of ISO/TS 20428 elements and a specification spreadsheet was derived (see Multimedia Appendix 1). Both the required and optional fields in ISO/TS 20428 were considered. In the case of inadequate or undefined resources in FHIR Genomics, new extension definitions were suggested, including datatypes, when there was a need for supplementation. We used the Forge program tool (Firely), the official HL7 FHIR profile editor, for FHIR profiling [19]. It allows the modelers to easily create, edit, and validate the FHIR profiles, extensions, and implementation guides. The users can fetch and publish FHIR resources and profiles. Forge enables users to create their own FHIR profiles, based on the FHIR base resources [19]. When using these extensions, the extension registry at the HL7 website [20] or Simplifier.net has to be examined to identify the already-defined extensions that may be suitable for appropriate needs. For example, a patient profile can be extended with the place of birth with an already-existing extension from the HL7 extension registry. Based on the FHIR server infrastructure, SNUBH EHRs used the REST API of the FHIR server to obtain the genome analysis results of a specific patient and provided a service that shows the genome analysis results to a clinician through a web interface.

Regarding data security and privacy, we applied Transport Layer Security (TLS) and OAuth 2.0, which is an industry-standard protocol for the authorization process [21]. The TLS was used for encryption of transmission data and authentication for trusted servers. OAuth 2.0 was used to allow only authorized users to have limited access to FHIR resources. Access tokens were used for security credentials for applications to make API requests on the behalf of a user. These tokens represent the authorization of a specific application to access specific parts of a user’s data [21].

## Results

### Mapping Table for Resource and Extension Definitions of ISO/TS 20428 to FHIR

A total of 32 data elements were included in the required fields of ISO/TS 20428 for patient information, type of sample, variant information, and recommended treatments. In the nomenclature of optional data, 29 items were defined to deliver more detailed information for biomedical and translational research. Five existing FHIR resources—ProcedureRequest, DiagnosticReport, Observation, Medication, and Patient—were used to represent the required fields of ISO/TS 20428. ProcedureRequest-genetics is a record of a request for a service such as diagnostic investigations, treatments, or operations to be performed; its name is changed to ServiceRequest-genetics. The DiagnosticReport-genetics profile contains the findings and interpretation of diagnostic tests performed on patients or groups of patients and on devices, locations, and/or specimens derived from these. The report includes the clinical context, such as request and provider information, and a combination of analysis results, images, textual and coded interpretations, and formatted representations of diagnostic reports. The required fields in ISO/TS20428 include clinical sequencing order information; information about the subject of care; and information about the person legally authorized to make the order, the performing laboratory, biomaterials, and genetic variations. Therefore, existing FHIR genetics elements sufficiently cover the genetic reporting standard as well. The other five targeted FHIR resources—Condition, DiagnosticReport, MolecularSequence, Observation, and Device—were leveraged and mapped by the ISO/TS 20428 standard’s optional field elements.

The ISO/TS 20428 standard and detailed data element definitions were reviewed. Opinion gathering and decision making were conducted through workshops in such a way that the individual data elements of the ISO/TS 20428 standard were mapped to the FHIR Genomics resource. The data items of the ISO/TS 20428 standard and the FHIR Genomics resource were made into mapping tables and an analysis workflow based on this table was created.

Table 1 shows part of the ISO/TS 20428 standard and FHIR resource mapping table. It indicates how the data elements in the ISO/TS 20428 genetic analysis reporting standard are mapped to the existing FHIR Genomics resource. Multimedia Appendix 1 shows the full mapping table.

Table 2 shows the number of FHIR Genomics resources in ISO/TS 20428. The Observation resource includes the most data elements, whereas the Medication resource contains only one subelement for the ISO/TS 20428 standard’s required fields. The existing FHIR resources must be defined to cover more content for the ISO/TS 20428 standard’s optional field that focuses on clinical trials and translational biomedical research. To manipulate the detailed sequencing information on quality control metrics, base calling, sequencing platform, and analysis platform, additional FHIR extensions should be defined in this study.

**Table 1:** Specification example of the International Organization for Standardization Technical Specification (ISO/TS) 20428 standard and the Fast Healthcare Interoperability Resources (FHIR) Genomics resources.

ISO/TS 20428 standard					Example data				Maps to FHIR Genomics resources (proposed)			
Data elements			Metadata		Value		Representation		Target FHIR resource		FHIR.Xpath	
**Information on subject of care**												
	Identifiers			ISO/TS 22220:2011		12345678		12345678		DiagnosticReport		DiagnosticReport.subject(patient)
	Name			ISO/TS 22220:2011		Gildong Hong		Gildong Hong		DiagnosticReport		DiagnosticReport.subject(patient)
	Birth date			ISO 8601		1947-04-29		April 29, 1947		DiagnosticReport		DiagnosticReport.subject(patient)
	Sex			ISO/TS 22220:2011		1		Male		DiagnosticReport		DiagnosticReport.subject(patient)
	Ethnicity			Health Level 7 (HL7) version 3 code system race		2040-4		Korean		DiagnosticReport		DiagnosticReport.subject(patient).extension(ethnicity)
**Genetic variation**												
	Gene symbols and names			Human Genome Organisation (HUGO) Gene Nomenclature Committee (HGNC)		HGNC:1097, B-Raf proto-oncogene (BRAF)		BRAF		Observation		Observation.extension(observation-geneticsGene)
	**Sequence variation information**											
		Notation		Human Genome Variation Society (HGVS)		c.1799T > A_p.V600E		c.1799T > A_p.V600E, Kinase domain (exon 15)		Observation		Observation.extension(observation-geneticsDNASequenceVariantName)
		Effects of variants		Text		Substitution (missense)		Substitution (missense)		Observation		Observation.extension(observation-geneticsDNASequenceVariantType)
		Sequence variant ID		Database unique ID		COSM476		Catalogue of Somatic Mutations in Cancer (COSMIC) website		Observation		Observation.extension(observation-geneticsDNAVariantId)

**Table 2:** Number of data elements per Fast Healthcare Interoperability Resources (FHIR) Genomics resource mapped by the International Organization for Standardization Technical Specification (ISO/TS) 20428 standard.

Standard field and FHIR resources		Number of elements in each resource	Number of extension elements
**ISO/TS 20428 required field**			
	ProcedureRequest	3	0
	DiagnosticReport	12	5
	Observation	8	8
	Medication	1	0
	Patient	2	2
	Total	26	15
**ISO/TS 20428 optional field**			
	Condition	1	0
	DiagnosticReport	2	2
	MolecularSequence	16	10
	Observation	6	6
	Device	3	3
	Total	28	21

### Implementation of ISO/TS 20428 Standard and FHIR Genomics Resources

**Figure 1:**
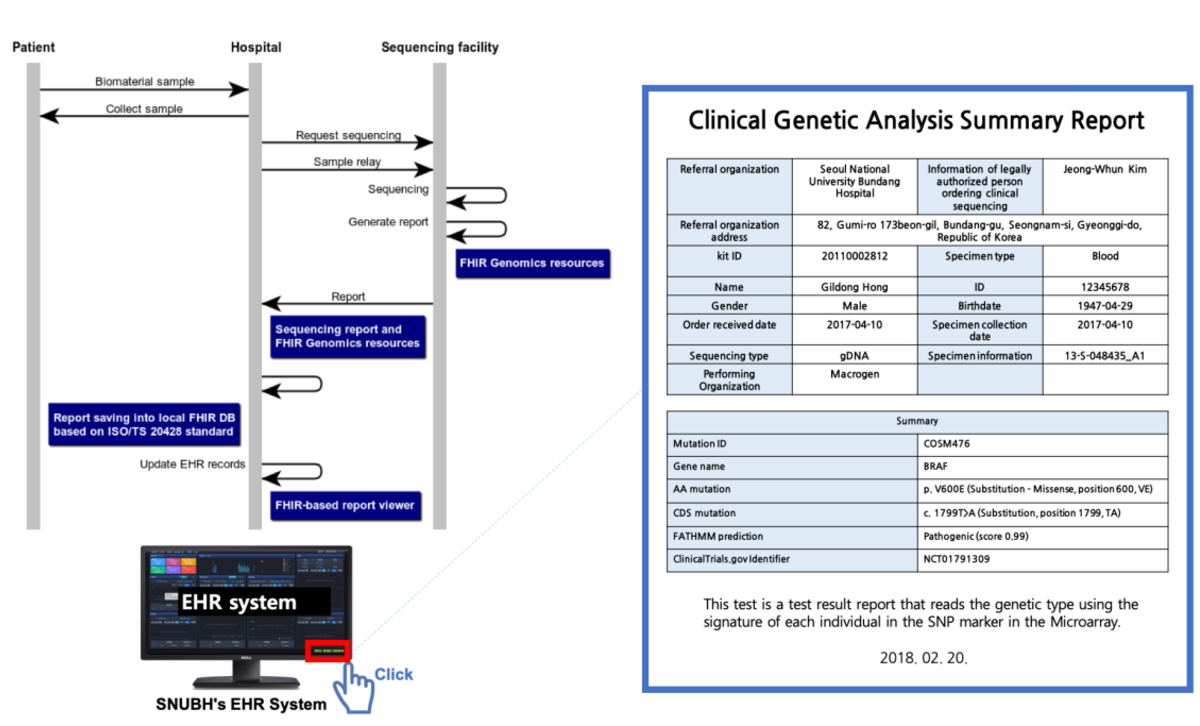
Implementation of the clinical next generation sequencing (NGS) report viewer. DB: database; EHR: electronic health record: FHIR: Fast Healthcare Interoperability Resources; ISO/TS: International Organization for Standardization Technical Specification; SNP: single nucleotide polymorphism; SNUBH: Seoul National University Bundang Hospital.

A bundled FHIR resource was created for clinical sequencing. It was delivered to the hospital from the sequencing facility and reconstructed in the clinician interface. The clinical sequencing report interface is populated with information about the variant results of the test, a summary, and information about the test (see Figure 1). Finally, we tested the feasibility of the ISO/TS 20428 standard on FHIR by building a clinician interface with sequencing from the sequencing facility. On this interface, patient data and FHIR Genomics resources from the FHIR server were combined. The clinical genetic analysis summary report consists of basic metadata for the sequencing order and a summary of the sequencing results. Data could be accessed, presented, and delivered with other types of patient information from the FHIR server. The clinical genetic analysis summary report was populated with information about the interpretation summary and the test. Multimedia Appendix 2 describes sample testing data for diagnostic requests.

## Discussion

### Principal Findings

To implement standardized machine-processable clinical sequencing reports in an EHR system, the ISO/TS 20428 international standard was proposed for a structured template.

Genomic sequencing laboratory reports are still provided in PDF or text formats in many clinical practices. Several difficulties, therefore, arise in clinical applications of these results due to unstructured image-based or narrative text–based genomic sequencing reports [22,23]. The ISO/TS 20428 standard was established to solve the variability in NGS reporting. It also defines the composition of a structured clinical sequencing report, which consists of the required and optional data fields and their metadata for a structured clinical sequencing report.

HL7 FHIR introduced web programming techniques to provide all standards and related resources in JSON (JavaScript Object Notation) or XML, making it easy for developers to implement. In particular, Sync for Genes, one of the pilot programs addressing significant technical challenges in this area, aims to standardize the sharing of genetic information by building FHIR Genomics. To this end, a pilot program involving laboratories, providers, governments, developers, and patients is developing ways to convert each participant’s internal format to the FHIR Genomics format. Sync for Genes aims to use HL7's FHIR as the basis for data exchange to enable sharing of genetic information and, ultimately, to incorporate clinically tested genomes into patient care [24]. FHIR Genomics resources predominantly focus on transmitting or representing sequencing data, which is of less clinical value.

Genetic test reporting standards are beginning to develop in many standard development organizations, and there are no standard implementation guidelines for data items from the proposed standard at the clinical site and no guidelines or references for implementing gene sequencing data results for clinical use. This is a significant challenge for implementation and application of these standards at individual sites. The goal of this pilot was to facilitate the reporting and viewing of genomic data for clinical applications.

In this study, we developed a prototype interface based on ISO and HL7 FHIR standards and verified its field applicability. Further, we demonstrated that genomic data reporting can be successfully integrated with an EHR system based on these two standards. We described the design and delivery of a clinical genome sequencing report, including a summary suitable for interpretation by clinicians.

Lessons learned from this study are as follows. First, to manipulate the detailed sequencing information on quality control metrics, base calling, the sequencing platform, and the analysis platform, additional FHIR extensions were required to be defined. For this prototype, FHIR Genomics required a new source, *extension*, which was introduced in this study. For example, sequence variation information such as “c.1799T > A_p.V600E, Kinase domain (exon 15)” is related to the Observation resource domain, but there is no specific entity that is appropriate to contain the associated expression. Thus, we defined the Observation extension as *Observation.extension(observation-geneticsDNASequenceVariantName)*. In addition, pathological tier information, which is written as “Tier 1 (Pathogenic, Identified),” was divided into three extension entities: (1) *Observation.extension(observation-classificationVariants).Pathogeny* to express “Pathogenic,” “Likely pathogenic,” “Unknown significance,” “Likely benign,” and “Benign”; (2) *Observation.extension(observation-classificationVariants).Tier* to express “Tier 1”; and (3) *Observation.extension(observation-classificationVariants).ClinicalRelavance* to express “Identified,” “Likely identified,” “Uncertain,” and “Not identified.” In addition, the existing FHIR resources must be defined in order to cover more content for the ISO/TS optional field that focuses on clinical trials and translational biomedical research. Second, we found several challenges related to the lack of terminology standardization in the genomics domain. The HL7 FHIR standard could be easily applied to EHR systems to incorporate the genome sequencing reports for clinical practice. However, semantic interoperability should be further improved by standardizing a vocabulary to describe genetic mutations. Third, as the level of detail in reporting clinical genomic sequencing varies between hospitals, the ISO/TS 20428 standard needs to be expanded to meet the requirements of various clinicians. The development and evaluation of clinical decision support systems that utilize the standardized sequencing reports should also be considered in future research. Lastly, during this implementation, it was also found that the system integration workflow between the EHR system and the sequencing facility should be designed to fit clinical process in a secure manner. The OAuth 2.0 authentication technique and dynamic informed consent–based data sharing by patients should be considered for protecting patient data as well. Further study of usability and screen interface design is needed for individuals using mobile apps in the genome sequencing workflow and with physicians in verifying and using the genomic data in the EHR system.

As a limitation of this study, since the standard interface was implemented and tested only for one EHR system in Korea, the standard implementation may be restricted depending on the EHR system level. In addition, as previously mentioned, because the usability test was not conducted on patients and medical professionals, there was a limitation that the system performance and usability of the integrated system using the FHIR standard could not be verified. As the FHIR standards are evolving rapidly, a review of the latest standards may be needed again.

With the pipeline definition for technical frameworks and the mapping resource presented in this study, we hope that the ISO and HL7 standards will be applied to real genomic data at various institutions.

### Conclusions

Sequencing laboratories still provide their reports primarily in unstructured formats, making it challenging to leverage or populate EHR data. To share and apply genomic data, especially for clinical practice, it is crucial to identify the applicability of the standard-based information system infrastructure in a practical setting. With existing FHIR resources, this prototyping study showed that reporting data from clinical genomics sequencing can be effectively stored and derived for EHRs for use by clinicians using the ISO standard and FHIR resources. We implemented a standardized clinical genomic sequencing report using the ISO/TS 20428 standard and FHIR. This systematic infrastructure is expected to enable the transfer and observation of clinical genomic sequencing reports in actual clinical applications.

## Appendix

Multimedia Appendix 1 The mapping table of the International Organization for Standardization Technical Specification (ISO/TS) 20428 standard and Fast Healthcare Interoperability Resources (FHIR) Genomics resources.

Multimedia Appendix 2 BundleTransactionRequest.json file.

## Abbreviations

API: application programming interface
EHR: electronic health record
FHIR: Fast Healthcare Interoperability Resources
HL7: Health Level 7
HLA: human leukocyte antigen
ICT: information and communications technology
ISO: International Organization for Standardization
JSON: JavaScript Object Notation
MSIT: Ministry of Science and ICT
NGS: next generation sequencing
NRF: National Research Foundation of Korea
REST: Representational State Transfer
SNUBH: Seoul National University Bundang Hospital
STU3: Standard Trial Use 3
TC: Technical Committee
TLS: Transport Layer Security
TS: Technical Specification
