# Supplementary material for: Clinical Genomic Sequencing Reports in Electronic Health Record Systems Based on International Standards: Implementation Study
Source: J Med Internet Res. 2020 Aug 10;22(8):e15040. doi: 10.2196/15040 (PMC7445611; doi:10.2196/15040)
Supplement: Multimedia Appendix 1 [file jmir_v22i8e15040_app1.docx]

| **ISO/TC 20428 standards** | | | | **Metadata (Primary)** | **Value**^b^ | **Representation**^c^ | **FHIR Genomics resources** | |
| --- | --- | --- | --- | --- | --- | --- | --- | --- |
| **Data elements** | | | |  |  |  | **Target FHIR Resource** | **FHIR.Xpath** |
| Clinical sequencing orders | | Clinical sequencing order code | Order code | LOINC | 14-RM-0000056 | 14-RM-0000056 | ProcedureRequest | ProcedureRequest.code |
|  |  |  | Information on sequencing order | TEXT | OncoPanel_V2 (Targeted NGS for 505 genes, T/N pair) | OncoPanel_V2 (Targeted NGS for 505 genes, T/N pair) | ProcedureRequest |  |
|  |  | Date and time | Order date | ISO 8601 | 2014–04–14 | April 14, 2014 | ProcedureRequest | ProcedureRequest.authoredOn |
|  |  |  | Specimen collection date |  | 2014–04–17 | April 17, 2014 | DiagnosticReport | DiagnosticReport.specimen.collected |
|  |  |  | Order received date |  | 2014–04–25 | April 25, 2014 | **DiagnosticReport** | **DiagnosticReport.Extension(orderReceivedDate)** |
|  |  |  | Report date |  | 2014–05–08 | May 08, 2014 | DiagnosticReport | DiagnosticReport.issued |
|  |  |  | Addendum creation date |  |  |  | **DiagnosticReport** | **DiagnosticReport.Extension(AddendumDate)** |
|  |  | Specimen information | | ISO/TS 22220:2011 | 13-S-048435_A1 | 13-S-048435_A1 | DiagnosticReport | DiagnosticReport.specimen |
| Information on subject of care | | Identifiers | | ISO/TS 22220:2011 | 12345678 | 12345678 | DiagnosticReport | DiagnosticReport.subject(Patient) |
|  |  | Name | |  | Gildong Hong | Gildong Hong |  | DiagnosticReport.subject(Patient) |
|  |  | Birth date | | ISO 8601 | 1947–04–29 | April 29, 1947 |  | DiagnosticReport.subject(Patient) |
|  |  | Sex | | ISO/TS 22220:2011 | 1 | Male |  | DiagnosticReport.subject(Patient) |
|  |  | Ethnicity | | HL7 v3 Code System Race | 2040–4 | Korean |  | **Patient.Extension(Ethnicity)** |
| Information of legally authorized person ordering clinical sequencing | | | | ISO/TS 27527:2010 | D060001 | Chulsoo Kim | DiagnosticReport | ProcedureRequest.requester.agent(Practitioner) |
| Performing laboratory | | Basic information | | TEXT | AMC Genomic Pathology Lab | AMC Genomic Pathology Lab, 02–3010–8460 | DiagnosticReport | DiagnosticReport.performer.actor(Organization) |
|  |  | Information of report generator | | TEXT | D110001 | Min Lee | **DiagnosticReport** | **DiagnosticReport.performer.actor(Practitioner)** |
|  |  | Information of legally confirmed person on sequencing report | | ISO/TS 27527:2010 | D030001 | Sejin Kim | **DiagnosticReport** | **DiagnosticReport.performer.actor(Practitioner) DiagnosticReport.performer.role** |
| Associated diseases and phenotypes | | | | ICD | C34.90 | C34.90 Malignant neoplasm of unspecified part of unspecified bronchus or lung | DiagnosticReport | DiagnosticReport.codedDiagnosis |
| Biomaterial information | Type of sample | | | SPREC | CEN | Non-blood tissue | DiagnosticReport | DiagnosticReportspecimen.type |
|  | Genomic source class in biomaterial | | | LOINC | 2 | Somatic | Observation | **Observation.Extension.(observation-geneticsgenomicsourceclass)** |
|  | **Conditions of specimen** | | | **TEXT** | Acceptable, Tumor proportion of the tested tissues (%): 90 | **Acceptable, Tumor proportion of the tested tissues (%): 90** | **Specimen** | **DiagnosticReport.specimen.Extension.(observedStatus)** |
| Genetic variations | Gene symbols and names | | | HGNC | HGNC:1097, BRAF | BRAF | Observation | **Observation.Extension (observation-geneticsGene)** |
|  | Sequence variation information | | Notation | HGVS | c.1799T > A_p.V600E | c.1799T > A_p.V600E, Kinase domain (exon 15) | Observation | **Observation.Extension (observation-geneticsDNASequenceVariantName)** |
|  |  |  | Effects of variants | TEXT | Substitution (missense) | Substitution (missense) | Observation | **Observation.Extension (observation-geneticsDNASequenceVariantType)** |
|  |  |  | Sequence variant ID | Database unique ID | COSM476 | <http://cancer.sanger.ac.uk/cosmic/mutation/overview?id=476> | Observation | **Observation.Extension (observation-geneticsDNAVariantId)** |
| Classification of variants | Pathogeny | | | ENUM (“Pathogenic”, “Likely pathogenic”, “Unknown significance”, “Likely benign”, “Benign”)^a^ | Pathogenic | Tier 1 (Pathogenic, Identified) | Observation | **Observation.Extension(observation-classificationVariants).Pathogeny** |
|  | Tier | | | Tier 1 (Pathogenic, Identified) | Tier 1 (Pathogenic, Identified) |  | Observation | **Observation.Extension(observation-classificationVariants).Tier** |
|  | Clinical relevance | | | ENUM (“Identified,” “Likely identified”, “Uncertain”, “Not identified”) | Identified |  | Observation | **Observation.Extension(observation-classificationVariants).ClinicalRelavance** |
| Recommended treatment | Medication | | | ISO 11615 | Vemurafenib, L01XE15 (ATC code) | Vemurafenib | Medication | Medication.code |
|  | Clinical trial information | | | Clinical trial ID | NCT01791309 | Vemurafenib and Panitumumab Combination Therapy in Patients with BRAF V600E Mutated Metastatic Colourectal Cancer  (https://clinicaltrials.gov/ct2/show/NCT01791309) | Patient | **Patient.Extension(preferredClinicalTrial)** |
|  | Known protocols related to a variant | | | TEXT |  |  | DiagnosticReport | **DiagnosticReport.Extension(establishedguidelines)** |
|  | Other recommendation | | | TEXT |  |  | DiagnosticReport | **DiagnosticReport.Extension(notes)** |

# Optional Fields

| **ISO/TC 20428 standards** | | | **Metadata (Primary)** | **Value**^b^ | **Representation**^c^ | **FHIR Genomics resources** | |
| --- | --- | --- | --- | --- | --- | --- | --- |
| **Data elements** | | |  |  |  | **Target FHIR Resource** | **FHIR.Xpath** |
| Medical history | | | ICD | N/A | N/A | **Condition** | **Condition.code** |
| Family history/Pedigree information | | | HL7 v3 IG: Family History/Pedigree Interoperability, | N/A | N/A | **DiagnosticReport** | **DiagnosticReport.Extension (DiagnosticReport-geneticsFamilyMemberHistory)** |
| Reference genome version | | | Genome Reference Consortium Human Genome release ID | GRCh37.p13 | GRCh37 Patch Release 13 (Released June 28, 2013) | Sequence | Sequence.referenceSeq.genomeBuild |
| Racial genome information | | | TEXT | 1000 Genomes | 1000 Genomes | Sequence | **Sequence.repository.datasetId** |
| Genetic variation | Gene symbols and names | | HGNC | HGNC:11998, TP53 HGNC:17278, PNRC1 HGNC:30988, BNC2 | TP53 PRNC1 BNC2 | Observation | **Observation.Extension (observation-geneticsGene)** |
|  | Sequence variation information | Notation | HGVS |  |  | Observation | **Observation.Extension (observation-geneticsDNASequenceVariantName)** |
|  |  | Effects of variants | TEXT |  |  | Observation | **Observation.Extension (observation-geneticsDNASequenceVariantType)** |
|  |  | Sequence variant ID | Database unique ID |  |  | Observation | **Observation.Extension (observation-geneticsDNAVariantId)** |
|  | **HGVS version** | | **HGVS version number** | **HGVS Version 15.11** | **HGVS Version 15.11** | **Observation** | **Observation.Extension(observation-HGVSversion)** |
| Detailed sequencing information | Clinical sequencing date | | ISO 8601 | 2014–05–04 | May 04, 2014 | Sequence | **Sequence.Extension(sequence-sequencingCompleteDate)** |
|  |  |  |  |  |  |  |  |
|  | Quality control metrics | Number of total reads | NUMERIC | 6950582 | 6,950,582 bp | Sequence | Sequence.quality Sequence.quality.method |
|  |  |  |  |  |  | Sequence | Sequence.quality Sequence.Extension(Unit) |
|  |  | Mean target coverage |  | 121.2 | 121.2 X | Sequence | Sequence.readCoverage Sequence.Extension(Unit) |
|  |  | % targets bases covered 100X |  | 93.1 | 93.1 % | Sequence | **Sequence.Extension(sequence-readCoverage-unit.structuredefinition)** |
|  | Base calling information | Read depth | NUMERIC |  |  | Sequence | **Sequence.Extension(sequence-meanDepth)** |
|  |  | Reference allelic depth |  |  |  |  | **Sequence.Extension(sequence-meanDepth)** |
|  |  | Alternative allelic depth |  |  |  |  | **Sequence.Extension(sequence-meanDepth)** |
|  |  | Allele frequency |  |  |  | Observation | Observation.Extension (observation-geneticsAllelicFrequency) |
|  |  | Genotype |  |  |  |  |  |
|  | Sequencing platform information | Type of sequencers | TEXT | MiSeq | Illumina MiSeq | Sequence | **Device.Extension(device-platformType)** |
|  |  | Library preparation methods |  | SureSelectXT Reagent Kit, HSQ 96 | SureSelectXT Reagent Kit, HSQ 96 |  | **Device.Extension(device-libaryType)** |
|  |  | Target capture methods |  | SureSelect Biotinylated RNA library “Baits” (SureSelectXT Custom panel) | SureSelect Biotinylated RNA library “Baits” (SureSelectXT Custom panel) |  | **Device.Extension(device-fragmentation)** |
|  |  | Read type | ENUM (“single-ended”, “pair-ended”) | pair-ended | Paired Ends | Sequence | **Sequence.Extension(sequence-read)** |
|  |  | Read length | TEXT | 150 | 150bp (75x2) | Sequence | **Sequence.Extension(sequence-readCoverage)** |
|  | Analysis platform information | Alignment tools | TEXT | BWA | BWA-MEM 0.7.12 | Sequence | **Sequence.Extension(sequence-softwareTool.alignment)** |
|  |  | Variant calling tools |  | SNV (Mutect2) indel (somatic indellocator) CNV (ReCapSeg) Structural variation (BreakMer 0.0.2) | SNV (Mutect2) indel (somatic indellocator) CNV (ReCapSeg) Structural variation (BreakMer 0.0.2) | Sequence | **Sequence.Extension(sequence-softwareTool.VariantCalling)** |
|  |  | Other tools |  |  |  | ?????? |  |
|  |  | Chromosome coordination system | ENUM (“zero-based”, “one-based”, “zero-based, half-open”) | one-based | one-based | Sequence | Sequence.coordinateSystem |
|  |  | Annotation tools and databases | TEXT | VEP: Variant Effect Predictor version 85 | VEP: Variant Effect Predictor version 85 | Sequence | **Sequence.Extension(sequence-softwareTool)** |
| References | | | TEXT | PMID: 12068308 PMID: 12198537 | [Davies H, Bignell GR, Cox C, et al., Mutations of the BRAF gene in human cancer. Nature. 2002 Jun 27;417(6892):949–54. (https://www.ncbi.nlm.nih.gov/pubmed/12068308) Rajagopalan H, Bardelli A, Lengauer C, et al., Tumorigenesis: RAF/RAS oncogenes and mismatch-repair status. Nature. 2002 Aug 29;418(6901):934.](https://www.ncbi.nlm.nih.gov/pubmed/12068308) | DiagnosticReport | **DiagnosticReport.Extension(references)** |
