# Supplementary material for: Clinical Genomic Sequencing Reports in Electronic Health Record Systems Based on International Standards: Implementation Study
Source: J Med Internet Res. 2020 Aug 10;22(8):e15040. doi: 10.2196/15040 (PMC7445611; doi:10.2196/15040)
Supplement: Multimedia Appendix 2 [file jmir_v22i8e15040_app2.docx]

{

"resourceType": "Bundle",

"type": "transaction",

"entry": [{

"fullUrl": "urn:uuid:a25c8171-6a7f-4915-986f-1dba98d0d57c",

"resource": {

"resourceType": "ProcedureRequest",

"status": "completed",

"intent": "original-order",

"code": {

"coding": [{

"display": "nextGenerationSequencing"

}, {

"display": "HiSeqXTen"

}]

},

"subject": {

"reference": "Patient/04294"

},

"authoredOn": "2017-06-20T11:01:00+09:00"

},

"request": {

"method": "POST",

"url": "ProcedureRequest"

}

}, {

"fullUrl": "urn:uuid:11a3459e-2d16-4d1b-b4c7-681f8a303d15",

"resource": {

"resourceType": "DiagnosticReport",

"meta": {

"profile": ["http://211.253.251.80:19080/FHIR/StructureDefinition/snubh-diagnosticReport-genetics"]

},

"contained": [{

"resourceType": "Patient",

"id": "04294",

"identifier": [{

"value": "kitId"

}]

}],

"extension": [{

"url": "http://211.253.251.80:19080/FHIR/StructureDefinition/extension-diagnosticReport-orderreceiveddate",

"extension": [{

"url": "SampleQC.beginAt",

"valueDateTime": "2017-06-16T14:47:42+09:00"

}, {

"url": "SampleQC.finishAt",

"valueDateTime": "2017-06-16T15:58:03+09:00"

}, {

"url": "LibraryQC.beginAt",

"valueDateTime": "2017-06-21T18:17:20+09:00"

}, {

"url": "LibraryQC.finishAt",

"valueDateTime": "2017-06-23T10:48:45+09:00"

}, {

"url": "Fragmentation.beginAt",

"valueDateTime": "2017-06-16T19:58:10+09:00"

}, {

"url": "Fragmentation.finishAt",

"valueDateTime": "2017-06-21T17:12:18+09:00"

}, {

"url": "Amplification.beginAt",

"valueDateTime": "2017-06-23T16:02:49+09:00"

}, {

"url": "Amplification.finishAt",

"valueDateTime": "2017-06-23T16:02:49+09:00"

}, {

"url": "Amplification.beginAt",

"valueDateTime": "2017-06-23T16:02:49+09:00"

}, {

"url": "Amplification.finishAt",

"valueDateTime": "2017-06-23T16:02:49+09:00"

}, {

"url": "Amplification.beginAt",

"valueDateTime": "2017-06-23T16:02:49+09:00"

}, {

"url": "Amplification.finishAt",

"valueDateTime": "2017-06-23T16:02:49+09:00"

}, {

"url": "Amplification.beginAt",

"valueDateTime": "2017-06-23T16:02:49+09:00"

}, {

"url": "Amplification.finishAt",

"valueDateTime": "2017-06-23T16:02:49+09:00"

}, {

"url": "Amplification.beginAt",

"valueDateTime": "2017-06-23T16:02:49+09:00"

}, {

"url": "Amplification.finishAt",

"valueDateTime": "2017-06-23T16:02:49+09:00"

}, {

"url": "Amplification.beginAt",

"valueDateTime": "2017-06-23T16:02:49+09:00"

}, {

"url": "Amplification.finishAt",

"valueDateTime": "2017-06-23T16:02:49+09:00"

}, {

"url": "Amplification.beginAt",

"valueDateTime": "2017-06-23T16:02:49+09:00"

}, {

"url": "Amplification.finishAt",

"valueDateTime": "2017-06-23T16:02:49+09:00"

}, {

"url": "Amplification.beginAt",

"valueDateTime": "2017-06-23T16:02:49+09:00"

}, {

"url": "Amplification.finishAt",

"valueDateTime": "2017-06-23T16:02:49+09:00"

}, {

"url": "InstrumentRunning.beginAt",

"valueDateTime": "2017-06-23T16:04:27+09:00"

}, {

"url": "InstrumentRunning.finishAt",

"valueDateTime": "2017-06-29T11:33:49+09:00"

}, {

"url": "InstrumentRunning.beginAt",

"valueDateTime": "2017-07-05T13:38:00+09:00"

}, {

"url": "InstrumentRunning.finishAt",

"valueDateTime": "2017-07-10T10:41:45+09:00"

}]

}, {

"url": "http://211.253.251.80:19080/FHIR/StructureDefinition/extension-diagnosticReport-references",

"valueString": "https://www.ncbi.nlm.nih.gov/snp/?term=rs583609"

}, {

"url": "http://211.253.251.80:19080/FHIR/StructureDefinition/extension-diagnosticReport-references",

"valueString": "https://www.ncbi.nlm.nih.gov/snp/?term=rs597470"

}, {

"url": "http://211.253.251.80:19080/FHIR/StructureDefinition/extension-diagnosticReport-references",

"valueString": "https://www.ncbi.nlm.nih.gov/snp/?term=rs597078"

}, {

"url": "http://211.253.251.80:19080/FHIR/StructureDefinition/extension-diagnosticReport-references",

"valueString": "https://www.ncbi.nlm.nih.gov/snp/?term=rs597076"

}, {

"url": "http://211.253.251.80:19080/FHIR/StructureDefinition/extension-diagnosticReport-references",

"valueString": "https://www.ncbi.nlm.nih.gov/snp/?term=rs35530163"

}, {

"url": "http://211.253.251.80:19080/FHIR/StructureDefinition/extension-diagnosticReport-references",

"valueString": "https://www.ncbi.nlm.nih.gov/snp/?term=rs397860579"

}, {

"url": "http://211.253.251.80:19080/FHIR/StructureDefinition/extension-diagnosticReport-references",

"valueString": "https://www.ncbi.nlm.nih.gov/snp/?term=rs35530163"

}, {

"url": "http://211.253.251.80:19080/FHIR/StructureDefinition/extension-diagnosticReport-references",

"valueString": "https://www.ncbi.nlm.nih.gov/snp/?term=rs630144"

}, {

"url": "http://211.253.251.80:19080/FHIR/StructureDefinition/extension-diagnosticReport-references",

"valueString": "https://www.ncbi.nlm.nih.gov/snp/?term=rs10714295"

}, {

"url": "http://211.253.251.80:19080/FHIR/StructureDefinition/extension-diagnosticReport-references",

"valueString": "https://www.ncbi.nlm.nih.gov/snp/?term=rs397860630"

}, {

"url": "http://211.253.251.80:19080/FHIR/StructureDefinition/extension-diagnosticReport-references",

"valueString": "https://www.ncbi.nlm.nih.gov/snp/?term=rs10714295"

}, {

"url": "http://211.253.251.80:19080/FHIR/StructureDefinition/extension-diagnosticReport-references",

"valueString": "https://www.ncbi.nlm.nih.gov/snp/?term=rs55842950"

}, {

"url": "http://211.253.251.80:19080/FHIR/StructureDefinition/extension-diagnosticReport-references",

"valueString": "https://www.ncbi.nlm.nih.gov/snp/?term=rs398089309"

}, {

"url": "http://211.253.251.80:19080/FHIR/StructureDefinition/extension-diagnosticReport-references",

"valueString": "https://www.ncbi.nlm.nih.gov/snp/?term=rs79346928"

}, {

"url": "http://211.253.251.80:19080/FHIR/StructureDefinition/extension-diagnosticReport-references",

"valueString": "https://www.ncbi.nlm.nih.gov/snp/?term=rs79346928"

}, {

"url": "http://211.253.251.80:19080/FHIR/StructureDefinition/extension-diagnosticReport-references",

"valueString": "https://www.ncbi.nlm.nih.gov/snp/?term=rs636523"

}, {

"url": "http://211.253.251.80:19080/FHIR/StructureDefinition/extension-diagnosticReport-references",

"valueString": "https://www.ncbi.nlm.nih.gov/snp/?term=rs636497"

}, {

"url": "http://211.253.251.80:19080/FHIR/StructureDefinition/extension-diagnosticReport-references",

"valueString": "https://www.ncbi.nlm.nih.gov/snp/?term=rs624660"

}, {

"url": "http://211.253.251.80:19080/FHIR/StructureDefinition/extension-diagnosticReport-references",

"valueString": "https://www.ncbi.nlm.nih.gov/snp/?term=rs634341"

}, {

"url": "http://211.253.251.80:19080/FHIR/StructureDefinition/extension-diagnosticReport-references",

"valueString": "https://www.ncbi.nlm.nih.gov/snp/?term=rs3913007"

}, {

"url": "http://211.253.251.80:19080/FHIR/StructureDefinition/extension-diagnosticReport-references",

"valueString": "https://www.ncbi.nlm.nih.gov/snp/?term=rs74176728"

}, {

"url": "http://211.253.251.80:19080/FHIR/StructureDefinition/extension-diagnosticReport-references",

"valueString": "https://www.ncbi.nlm.nih.gov/snp/?term=rs34664688"

}, {

"url": "http://211.253.251.80:19080/FHIR/StructureDefinition/extension-diagnosticReport-references",

"valueString": "https://www.ncbi.nlm.nih.gov/snp/?term=rs17798991"

}, {

"url": "http://211.253.251.80:19080/FHIR/StructureDefinition/extension-diagnosticReport-references",

"valueString": "https://www.ncbi.nlm.nih.gov/snp/?term=rs11664186"

}, {

"url": "http://211.253.251.80:19080/FHIR/StructureDefinition/extension-diagnosticReport-references",

"valueString": "https://www.ncbi.nlm.nih.gov/snp/?term=rs11872992"

}, {

"url": "http://211.253.251.80:19080/FHIR/StructureDefinition/extension-diagnosticReport-references",

"valueString": "https://www.ncbi.nlm.nih.gov/snp/?term=rs7506280"

}, {

"url": "http://211.253.251.80:19080/FHIR/StructureDefinition/extension-diagnosticReport-references",

"valueString": "https://www.ncbi.nlm.nih.gov/snp/?term=None"

}, {

"url": "http://211.253.251.80:19080/FHIR/StructureDefinition/extension-diagnosticReport-references",

"valueString": "https://www.ncbi.nlm.nih.gov/clinvar/RCV000009294"

}, {

"url": "http://211.253.251.80:19080/FHIR/StructureDefinition/extension-diagnosticReport-references",

"valueString": "https://www.ncbi.nlm.nih.gov/snp/?term=rs1260326"

}, {

"url": "http://211.253.251.80:19080/FHIR/StructureDefinition/extension-diagnosticReport-references",

"valueString": "https://www.ncbi.nlm.nih.gov/clinvar/RCV000009294"

}, {

"url": "http://211.253.251.80:19080/FHIR/StructureDefinition/extension-diagnosticReport-references",

"valueString": "https://www.ncbi.nlm.nih.gov/snp/?term=rs1260326"

}, {

"url": "http://211.253.251.80:19080/FHIR/StructureDefinition/extension-diagnosticReport-references",

"valueString": "https://www.ncbi.nlm.nih.gov/snp/?term=rs3817588"

}, {

"url": "http://211.253.251.80:19080/FHIR/StructureDefinition/extension-diagnosticReport-references",

"valueString": "https://www.ncbi.nlm.nih.gov/snp/?term=rs2180439"

}, {

"url": "http://211.253.251.80:19080/FHIR/StructureDefinition/extension-diagnosticReport-references",

"valueString": "https://www.ncbi.nlm.nih.gov/snp/?term=rs1160312"

}],

"identifier": [{

"use": "temp",

"value": "ed205acd-7ce8-42fa-8f2e-906a910f932b",

"assigner": {

"reference": "Organization/065dae06-f3c6-4f53-b1ee-cc3868ff60f6",

"display": "Macrogen"

}

}],

"basedOn": [{

"reference": "urn:uuid:a25c8171-6a7f-4915-986f-1dba98d0d57c"

}],

"status": "final",

"code": {

"coding": [{

"system": "http://loinc.org",

"code": "51969-4",

"display": "Genetic Analysis Report"

}]

},

"subject": {

"reference": "#04294",

"display": "kitId"

},

"issued": "2018-09-05T20:02:38.088+09:00",

"performer": [{

"actor": {

"reference": "Organization/065dae06-f3c6-4f53-b1ee-cc3868ff60f6",

"display": "Macrogen"

}

}, {

"role": {

"text": "BioinformticAnalysis"

},

"actor": {

"display": "?띻만??

}

}, {

"role": {

"text": "SampleQC"

},

"actor": {

"display": "源?뺣궓"

}

}, {

"role": {

"text": "LibraryQC"

},

"actor": {

"display": "?⑸???

}

}, {

"role": {

"text": "Amplification"

},

"actor": {

"display": "怨쎈???

}

}, {

"role": {

"text": "Amplification"

},

"actor": {

"display": "怨쎈???

}

}, {

"role": {

"text": "Amplification"

},

"actor": {

"display": "怨쎈???

}

}, {

"role": {

"text": "Amplification"

},

"actor": {

"display": "怨쎈???

}

}, {

"role": {

"text": "Amplification"

},

"actor": {

"display": "怨쎈???

}

}, {

"role": {

"text": "Amplification"

},

"actor": {

"display": "怨쎈???

}

}, {

"role": {

"text": "Amplification"

},

"actor": {

"display": "怨쎈???

}

}, {

"role": {

"text": "Amplification"

},

"actor": {

"display": "怨쎈???

}

}, {

"role": {

"text": "InstrumentRunning"

},

"actor": {

"display": "?꾩???

}

}, {

"role": {

"text": "InstrumentRunning"

},

"actor": {

"display": "?꾩???

}

}],

"specimen": [{

"reference": "urn:uuid:60b84035-6476-4c94-b917-9db1ef7c04fc"

}],

"presentedForm": [{

"contentType": "application/pdf",

"language": "kr",

"data": "JVBERi0xLjUNCiW1tbW1DQoxIDAgb2JqDQo8PC9UeXBlL0NhdGFsb2cvUGFnZXMgMiAwIFIvTGFuZyhrby1LUikgL1N0cnVjdFRyZWVSb290IDE0IDAgUi9NYXJrSW5mbzw8L01hcmtlZCB0cnVlPj4+Pg0KZW5kb2JqDQoyIDAgb2JqDQo8PC9UeXBlL1BhZ2VzL0NvdW50IDEvS2lkc1sgMyAwIFJdID4+DQplbmRvYmoNCjMgMCBvYmoNCjw8L1R5cGUvUGFnZS9QYXJlbnQgMiAwIFIvUmVzb3VyY2VzPDwvRXh0R1N0YXRlPDwvR1M1IDUgMCBSPj4vRm9udDw8L0YxIDYgMCBSL0YyIDExIDAgUj4+L1Byb2NTZXRbL1BERi9UZXh0L0ltYWdlQi9JbWFnZUMvSW1hZ2VJXSA+Pi9NZWRpYUJveFsgMCAwIDcyMCA1NDBdIC9Db250ZW50cyA0IDAgUi9Hcm91cDw8L1R5cGUvR3JvdXAvUy9UcmFuc3BhcmVuY3kvQ1MvRGV2aWNlUkdCPj4vVGFicy9TL1N0cnVjdFBhcmVudHMgMD4+DQplbmRvYmoNCjQgMCBvYmoNCjw8L0ZpbHRlci9GbGF0ZURlY29kZS9MZW5ndGggMjI1Pj4NCnN0cmVhbQ0KeJx9z01Lw0AUheH9wPyHs9SCybmT+SqEQJLGolCoOOCiuBLblYL6/8FJFUmIdH/nfc6g3KOuy11/twGbBt2mx4dWBAuSYgwDgiGcJT5ftXpa4V2rcvvocPrSSnD6Oya90M6ujyutHrTCsOuBiSS/Updy61YgEek41nIKgspYmFAVwSC9jf0zstXqUNNFYVu1dFXH2EvzjHSv1ZD+UcxMMQtl7QuaKXS4wvWFXrVYnd/beVNcIbnpTCHnJG74c/SSt/uwpnWeMQa2bqB37tJ+u9i/8CyzZ6ceR2r5j2+zimAsDQplbmRzdHJlYW0NCmVuZG9iag0KNSAwIG9iag0KPDwvVHlwZS9FeHRHU3RhdGUvQk0vTm9ybWFsL2NhIDE+Pg0KZW5kb2JqDQo2IDAgb2JqDQo8PC9UeXBlL0ZvbnQvU3VidHlwZS9UeXBlMC9CYXNlRm9udC9BQkNERUUruLzAuiMyMLDttfEvRW5jb2RpbmcvSWRlbnRpdHktSC9EZXNjZW5kYW50Rm9udHMgNyAwIFIvVG9Vbmljb2RlIDI0IDAgUj4+DQplbmRvYmoNCjcgMCBvYmoNClsgOCAwIFJdIA0KZW5kb2JqDQo4IDAgb2JqDQo8PC9CYXNlRm9udC9BQkNERUUruLzAuiMyMLDttfEvU3VidHlwZS9DSURGb250VHlwZTIvVHlwZS9Gb250L0NJRFRvR0lETWFwL0lkZW50aXR5L0RXIDEwMDAvQ0lEU3lzdGVtSW5mbyA5IDAgUi9Gb250RGVzY3JpcHRvciAxMCAwIFIvVyAyNiAwIFI+Pg0KZW5kb2JqDQo5IDAgb2JqDQo8PC9PcmRlcmluZyhJZGVudGl0eSkgL1JlZ2lzdHJ5KEFkb2JlKSAvU3VwcGxlbWVudCAwPj4NCmVuZG9iag0KMTAgMCBvYmoNCjw8L1R5cGUvRm9udERlc2NyaXB0b3IvRm9udE5hbWUvQUJDREVFK7i8wLojMjCw7bXxL0ZsYWdzIDMyL0l0YWxpY0FuZ2xlIDAvQXNjZW50IDEwODgvRGVzY2VudCAtMjAwL0NhcEhlaWdodCA4MDAvQXZnV2lkdGggNDYzL01heFdpZHRoIDIxNzUvRm9udFdlaWdodCA0MDAvWEhlaWdodCAyNTAvU3RlbVYgNDYvRm9udEJCb3hbIC05NzcgLTIwMCAxMTk5IDgwMF0gL0ZvbnRGaWxlMiAyNSAwIFI+Pg0KZW5kb2JqDQoxMSAwIG9iag0KPDwvVHlwZS9Gb250L1N1YnR5cGUvVHJ1ZVR5cGUvTmFtZS9GMi9CYXNlRm9udC9BQkNERUUruLzAuiMyMLDttfEvRW5jb2RpbmcvV2luQW5zaUVuY29kaW5nL0ZvbnREZXNjcmlwdG9yIDEyIDAgUi9GaXJzdENoYXIgMzIvTGFzdENoYXIgMzIvV2lkdGhzIDI3IDAgUj4+DQplbmRvYmoNCjEyIDAgb2JqDQo8PC9UeXBlL0ZvbnREZXNjcmlwdG9yL0ZvbnROYW1lL0FCQ0RFRSu4vMC6IzIwsO218S9GbGFncyAzMi9JdGFsaWNBbmdsZSAwL0FzY2VudCAxMDg4L0Rlc2NlbnQgLTIwMC9DYXBIZWlnaHQgODAwL0F2Z1dpZHRoIDQ2My9NYXhXaWR0aCAyMTc1L0ZvbnRXZWlnaHQgNDAwL1hIZWlnaHQgMjUwL1N0ZW1WIDQ2L0ZvbnRCQm94WyAtOTc3IC0yMDAgMTE5OSA4MDBdIC9Gb250RmlsZTIgMjUgMCBSPj4NCmVuZG9iag0KMTMgMCBvYmoNCjw8L0F1dGhvcij+/81cXAAgx3XIFSkgL0NyZWF0aW9uRGF0ZShEOjIwMTgwNzMxMTEwODUwKzA5JzAwJykgL01vZERhdGUoRDoyMDE4MDczMTExMDg1MCswOScwMCcpIC9Qcm9kdWNlcij+/wBNAGkAYwByAG8AcwBvAGYAdACuACAAUABvAHcAZQByAFAAbwBpAG4AdACuACAAMgAwADEAMCkgL0NyZWF0b3Io/v8ATQBpAGMAcgBvAHMAbwBmAHQArgAgAFAAbwB3AGUAcgBQAG8AaQBuAHQArgAgADIAMAAxADApID4+DQplbmRvYmoNCjIwIDAgb2JqDQo8PC9UeXBlL09ialN0bS9OIDkvRmlyc3QgNjAvRmlsdGVyL0ZsYXRlRGVjb2RlL0xlbmd0aCAzNDU+Pg0Kc3RyZWFtDQp4nI1SwYrCMBC9C/7D/EGatlYFEZZV2UWUYoU9yB5ina3FNpGYgv79TtKqle1hL8nMm3kvb6blI/DA92HAwQ+AeyH4HPhgCHxIcAScgDEVBhCMQuARRL4HfAxRGMBkwmLb58GGJSw5C8m2tzOyxOgqNfMCSxZnELj6ErzptN9zFHqhpsR/+pc7ICPf8CD+kxRA2EV6mNvi1ezVtYtKw1OPnZ6uTpHwPmGRH7DT8qiW4N0SLcZWI26UMmyjClyJs12sFY+FRumqdsduYbvGvdV7VNc0xxJvwBvpBWlJZZCt7TGXh2dyHznB1LAPFAfUdWw59/hTFrnE5CisQwu8SVIQJleyybXJfwQFLvtS+rRX6sRmKq1K8uSQyxHRWJOGrUSqVSt/P9LZyme5KFTWAuqdPnvrd6gt06JkizyrNDazrqvysgP397m1WPi5e//lCl6+ab/3C6C01sINCmVuZHN0cmVhbQ0KZW5kb2JqDQoyNCAwIG9iag0KPDwvRmlsdGVyL0ZsYXRlRGVjb2RlL0xlbmd0aCAyODk+Pg0Kc3RyZWFtDQp4nF2RTWrDMBCF9zqFluki2I4jWwZjcJQUvOgPdXsARxqngloWsrLw7TtSQgoVSPAx7w1vRonojp3RnibvbpY9eDpqoxws89VJoGe4aEOylCot/Z3iK6fBkgTN/bp4mDozzqSuafKBxcW7lW5aNZ/hiSRvToHT5kI3X6JH7q/W/sAExtOUNA1VMGKjl8G+DhPQJNq2ncK69usWPX+Kz9UC3UXObmHkrGCxgwQ3mAuQOsXT0PoZT0PAqH/16uY6j/J7cKjeswLVh7xMGySWHwJxJiLxLFAleKCCsUBHHpVFWQU67VkgzkskUZb7SCL4BN9FZZu3SMe0iD1bdgqU5WlMd88RcoZ1PpYgr87h/HHncfAwsjbw+BY72+AK9xf/hYZTDQplbmRzdHJlYW0NCmVuZG9iag0KMjUgMCBvYmoNCjw8L0ZpbHRlci9GbGF0ZURlY29kZS9MZW5ndGggMjAxMDkvTGVuZ3RoMSAyMDg1NjA+Pg0Kc3RyZWFtDQp4nOyZCXxU1b3H/+feO2smycwkE5IMyx0GEBhgMkQ2Tc0ACVtYAgSbsGgiSUggJCEJi2sCloIRFUTQulKf7bM2xQlgG3AhKggqCGrRalVw12rcsUiSmfc/594sUOqz/bRPff1/f9zfPffcc8/9n3VmAjAASERToC5j9uSJ4Sl7XgDZ/jFAr30TMzInwIDyNJA+r8NSvSdmz5i9JO7KXXi9Da+nTpw9Z9xLY56Mw2ss33v7tMxZEyEBGD5/J963zZjtH/74p34DgGTG6/yLM6blrpx+zdMAX94GYDixcGlB5fDqd6MBvlmF9+9buKJGLU9YVQjM/AGAPLi4ctHSIbf9YQjA6Z4AVuOigupKSIbzQAK8BvuissuLmw/dOxiYLR7vP1BSVFDoPHDfJAC2Ae+PLMEMa639FF7vw+t+JUtrVpUOjqrCeEoAPh+2pKiqHO6AVGBfpvD2lVUsLHj4ykemA0toBnitcmnBqsplr1ovwecxHlCXFtUUjE6v+hrbdz9eZ5QXLC1KvvyaZfh8Njb3t5UV1TXhalgJLNLGy1dWFVU+/3bvB4Ep/bCD963AeGzjEn6O8Q/h8azAeKqTTU68zubxAB8L42/X7qv0Hr00Nu2kauHdBvBYYFIDP7/YUHTvqZSwYrnVVI2XFnxOA58zPxR2YNYHeP9uy62ipm5Y83lO+SjsO0VkSGAHP8zHXh5uNWo58kNsIxjAbLjdkIpV9tLO0iNQLFWbDVKUQZb4c8pGAP+gl69qxWec/MFps1UVggCRdmNxuJhHwj4FsxaBkmzYy3sCZMM0eEA+jOlDWMlRPLJhjzwItsKPBNPF8OS/q27leSj6Z56T/ggN/+pYCIIgCIIgCIIgCIL44WEG5v2+YyAIogszwOrvO4YfIuaz/yxNEMQ/zQ9tn8H1PfX7joEgCIIgiB8H+D3ms+87hv8LsJ2bvu8YCIIgiH8PP8Df5IHvOwaCIAiCIAiCIAiCIAiCIAiCIAiCIAiCIAiCIAiCIAiCIAiCIAiCIAiCIAiCIAiCIAiCIAiCIAiCIAiCIAiCIAiCIAiCIAiCIAiCIAiCIAiCIAiCIAiCIAiCIAiCIAiCIAiCIAiCIAiCIAiCIAiCIAiCIAiCIAiCIAiCIAiCIAiCIAiCIAiCIAiCIAiCIAiCIAiCIAjiRwTj4oANTpkjYAZLJAwW9HawCo8CK7oNbOjREB1pgxiIRY8FO7odHOgOiEN3ggs9DuIjrRAPPdBdkISeILwHJKMnop+GJOiFngy90d3QB70n+jfQCzzovaEveh/woqvQP3IKPML7wgB0L5yH3g/9r9AfBqEPgMHo54EPfSAMRR+E/jUMBj+6DwLoQ2A4+lD0kzAMUtH9wlNgJHoA/SsYDqPQU9G/hPNhDPoI9C9gJFyAPgrS0EcLHwMXoV+A/jlcCOnoaTAO/ScwHv0iyIh8BumQiR6ECehjYSL6OPRPYTxMQs+AKeiZkIU+Af0TmAhT0SfBdPTJkI0+BWaiZ6G3wFSYhT4NZqNPhznoM9A/hmy4GH0m5KLPgjz02egfQQ7MQ58D89EvhgXoP4VLIn+BXOF5kI8+Fy5DnwcFkQ9hPhSiL4Ai9EugGP1SKEHPR/8ACmAx+mXCF8IS9EIoi7wPRVCOXgwV6IugEr0ElkXeg1KoQl8M1ehLoAa9DFZE3oWlsBK9XHgFrEKvhCvQl6G/A1VwFXq18Bq4Gn05XBN5G1ZALfpKWI2+CtagXw7XRt6CK4RfCT+LvAlXwVr0q2E95lyD/ibUop+AOqhHXw3Xo6+BG9CvRT8OP4Ob0NfCjeg/h43o69DfgPVwM/p1cAt6PWxBvx79ddgAt6LfALeh3wi3o9+E/hpshDvQN8Gd6DfDXeib4e7In+EW2Ia+BX6JvhXuRb8V/VW4De5D/wX8Cv12+DX6HeivwJ1wP/pd8Bv0u+EB9HugAX0b+p/gl7Ad/V54EP2/IIR+H+yIvAy/Ev5r2In+37AL/X70l+A38Af0B6AJ/bewG70B/Rj8Dvagb4eH0R+ER9FDsDfyR2gUvgOa0XfC4+i74InIi/AQPIn+e9iH/gd4Cr0JDqDvRn8B9sDT6A/DM+iPwCH0R9Gfh8eE74Xn0JvhCPrjcDRyFJ6A59GfFL4PXkTfD3+MHIGn4Bj6AXgJ/SD8Cf1p9OfgGXgF/Vl4Ff0QvIZ+GF6PHIbn4A1MH4Hj6EfhBOY8D29GDsEL8DamX4R30P8I72LOMfRn4SV4H9Mvwwfof4IPMecV9GfgVfgY03+GFvTX0J+B1+GTyNPwBnyKfhw+w5wT8AWm30Q/CG/BSfS34WvMeQf9ILyLfgDeg7+ivw+nMecD9APwIbRGnoK/QBv6R9COOR9DBNMtEI7sh08YoH/KJPTPmIL5nzMDpr/A9D74EtP74CtmxpyTzILpr9GfhL8yK/opFo0536A/CafRn4BWFoPexpyY047+BISZI/I4RDD9OO7wEh4yHvHoTJwVnLWAO6uKKU407qzTcEVX7axsmr/74KN1j2UeqDmoHi17PiUS0Uuo31KCRU5G3taU/d7U41m7Jx2bdP7YuOAvxzw3Zph457cSnDlvbl7OrGlTs6ZMnjRxwvhxY4PpF/0k7cILxoweNXLE+anDAyn+YUOH+AYPGnjegP79vH09ap/evXq6k5MSeyS44uOcDntsTLQtymoxm4wGRZYYDGGJocTxuZmLQ0nj80M2b4bXroZs0z+b5g+B0+3xOtRUf95QvVTI4AtBXFYoPju3EYKj80JG39lFpofk/vYvPPjwNLeaGVL64z/vlILC0MBZuR6v/SV35/08fCaUPD7X43GHpP74bzLewn9TCtTCkD0b8z1uLWdyCLJz+dEUeWs0ZsJoTx76rNxQ747LvLxzBbkbINJ8VpjTWb290ZY0PiME8Y1geysELl7ss9G4wNNCA30YiB1Tojbwh1j8FyEWF2KuaRjyma/gj50YfY4+yCxc7M0sLMUeLczv6tPPtB71qPVq/axcRyomRdBZoYMzcxujrOO944usmAEiAxqtUZgTxTOwispGZruIiYRky7ygUQJzNHafk4ebyY/FoeD1+ZjwZmC/4Z24rjtNkeYN3W8BPtaRitNSWhAh4/iQSQtCLQ0FC0Jwvdo4pLl+Q5MdLsv32Qq9hQXzc0NyARZoBLl/ZklOqGdW9lzMwlfhkV+i8uHOEMYHT80sUevxmpfNR/dm8EE/I7+wpCifTxOW783Ae5bxues8ze6QE8+ZIYcvFI3Foq94xy3XZyaWqvyyvn6dGtqG4Xa76+GOkyARQ6/P9OLbsLLMxeP4kPg7h03MxsmFYnCC1xeoobrLFmtzr2BDx/z31NtDtq89ODo4PvikeFDvysL8xTzkxQW8mZmL1frri0RTN4im4XxVMxdn8IM/iLMf5uDTc3MzS7yZXS/EhmNC7n/2sx5PKMnHH6yvz+QhFhRi9FrIeKMrfr4m3D6G8YwPBXPECXLEGOAbgwUZeXqWXmAuf4zfyc/Iy/No445FQ6b+6wzDvGo9r9HUPxTvs3v24b3moUOyZuVmZrhF60PS+NyftCS6WzCdld2ZzRKxTL2/xa31UdZsb9ZMbRaUdFh+jraApc6Rx6J6eVHr4UT3YUxP8E7Ir6+f4FUn1OfXFzRF6i7zqnZvfaPNVl+Zma+Klc8wf8/17tCEDXkhe34JuwAHmc+3CbOyQnEz5/HhmaCWFGibRbrXM9rtceR1lMn+e7f1dYYzHuc9X2f19o8xNhvuSG51At9emnBXcIfso/kyxUjm5OI6WCjmrDBcH7OxcjdfKXJe/8zS2XoH4WzUJwzf92bquViJx8PX0PVNQbgML0J1M3O1axUuc++AoN+HY5fP7zR33HHN4XfqOu50Pp7vxbFKzJr9v8zp7vO53uF1qmP8ov/FdlsYas7BNp4aHTKP1oc7bnyu7Jb0lOSWecrqw+0rLdTDJx7kfYK7ZL3dqx71huy+kGF8brM7LU+1O3B7Y1hmko+vGtxFj3qfZnzvhHh7iKWFWALPB9xLxZYu9xiNNzsnj5pZn6/Pru7N0j8ACkvO3TYsY/di89xaeYfTy1t4SGxp+k7dfwJfS26PVmJKXiiG78ehmI+FYbzu8bkq7j64WmeKhJqplvDBDqn5GWIbyHN3z26KnMjP4NsehsyLuPVpja517Zlz7bvP8Dqc4as35JXg7A4FB2ML1BH4WrFacnL1Xhrt1lcRf9dk3pQz73f2YkeZv+3drJwzrrrVK+6N7lz4ObmhCb6OerTriT5398tJZ92e3HEbd4er3VfwTwmJjWv0svUzG4Ns/ey5ufi5NW63Hb9Rrc/J3SExaXz+uLzGfng/d7eKX29ErsRzeSa/UPkFZDGscYdkFuXdu4MAdeKuIjLE9cImBiLP3JHHYGGTpOXZtRcNEC8K4ve8hU2KdifYUVrBPLOWVyfyBI3AGxa0GoLmoCVok6IldyPjWTswZw9+X7Mw2Glj0czdiE/NEtlNrK7REnRrJeqwRFCLcP2crlfPmZu70wb4mHB80TgO9mMd9mQ2rhB8xzz+fSmkJj/trrfz/TeU5+N71LtDsaNmHT+R0KPnH4+hXXlVgvvKq5KefwHTK1aiLa1EK6tAW1Ke4F5SXluVXLM83tVz0WK04lK0opJ4d1HJ2mXJSdUJV4xP8lyOhzI2B4NloLBa4dcIvwL9K5H/lcj/SuR/xfPZhWwkNsfHLtDPY/TzaP08Sj+P0M/ns+H4vdrHUvVzQD+nML84D9PPQ/XzEP2sMg8Y8dyH9QYrnnvv2GT0PcJ6sSSR3XPHFtnXxNw7tpjxlLxjiw1PSTs28avE4H2bYn2zpyq+U1sU31/xOInHL/DYhEdwS1zCmA3XGX3X43HdWqNvdS34rq41+moxvX4t+Nbh8TM8rsVr9yhX4kiXa4TLeb4rNtVlG+6yBFzGFJfsd8Ew14DzYgaeFzvYFzPEF9vXG9PPG9u7T4zaJzbW7rBZrFE2o8lskxWDDZhkix3cZ7Ck+li+77hPGmAs7ANjF7JC7Na9wu1sIXo2+hE8JFBZAV770ffiwa8vxet89Eo87sHjQTz24mHAnEugDo978HgQDwWOMgf+cHLgc0H0fDzq8JBFHn+LHT0FPYhHHR4yHGdOzHsQfS8eR/CQoRb9JjzuEVfZei1HRU0p6HrdwRHyjLaKtto2eUZ7Rbt0Uyub0Xppa0Wr3KetT7sU2+Zvu7RNvqltb9vxNrmi7cG2SJuc3saym5ghmMzS2x9sj7TLvLzkb01vndEqV7Y3t0sn9jIDBlSJvhGPZizcS64M14Wl2tZPW6Xa05+eltRwSjgYzg4rajtTeXUzmVEu9MWCH9KhAmrxF/ynYEqPrYuVYmXmZr2iE03J0S57j2inEh89JG1w2sC0AWn90vqmqWm909xpiWmuNGdabJolzZgmp0FadmoOCzmzICtnXCiO4Xn2uFCqL6tJVmeFhvuyQpbsebmNjN2Yh7khaT1uRTkhZT3uPjn4PXbuvFyckPz2WvduXDcQyspfe0Oez9crVMg/6ep65YWG88TGXnn4nWT4zJDbO853Lqqrq/mhUaOxXNCViYcmX83y6m7PNg4ckBkanFkQGpKZn9G9UtaVhHO+tdvbxUt9+F00lI5dcHaBRgvvi+xZ47JCZvxmZs6eF0r24sVBvBiJFzbvuEbc3PHHjiLfmGeMgySYb2hGny78DBQ35tyIP7De51ddHu4ZaeleTnpWehaK8XmX9NPIR3IJuLDcR2fWZZK7Xxl3RKq1lFk/tJ/2sAY2iPMyvWC58Kth4bf8IH8B9Y/zAB6/1NP34nGXnt4mjm2d5Tbr53Wd53XwXXkNXmPx7BQ7dI57a1AA+1HXQDbMQeWi1sANqGV4XndGPcKlHrAKz5fCFeJ6rfDL8FqLaaVeuhbK9NQqWCMNEH34BNwBW2ArbIJCqIYC+Xx5gnI5jAUwbAi/Ao9BlpyLPV+KPV3NEvG5O7DGTVCqXK7cAEPZic5IfgH34Iz4BfwKV/ONWKZQH6Gz4W/agPfXYI2r8I3lWHchzMVY52Brs75zD34LxmORqZHVRg9UGO823vS/vnEWzIdi/vdb+BnshifhMLwMb8JJiMDFkA+LoYb/HRZuhjvh13AMjsOH0I7vuM6YGnkkUmvcCvwvUzhnlHsNd+CGa4LkYJQCThOTDQaLAv7DzjH+w3hKPxxISXV4HP09Ds8D8qm2bdLT7aMMd5wuuk1ZgjXsiXzCtuBKkSEWBgTj8/Hrl2QKRcfJMshskiRZH1T8LWP8LanDIT29Zbh/QSCFyV5ZPn9k6vAEV7zR23cAW+Vc40zEhT8+ffDwoKG59c9K/9O3+4Pjhg4aH+RRbpVPSpvFO0wwKBhvNEmySZZlxWTINhot+J5sib/A2fEa+2H7Ye01HjxYRp/KPqy9d4VqaA6PYM/yg/+/y5PYG7xWN4wLepWvkmTbVw7Z7nSZneB01yZBgsmMC9ncy+60RUXF1tp5M1IPO5xjxvj99sNj7Pta8FWHsVHpLYEUj+P8UV6jiXnZgPO8CS6H15HKEnqkjhzFUh2GL2INiXHtkclhiE8wmO96kR0aHW2MHcNePiC/Vf2bhQNbb1CW+EpGvdXmxsZ/eU3KoHrFfFq0vCjyvtIP9zQ79IKhwUQpeYshgbmi47dE2WOhVzRcy1ifHtea/S2i7am8i1P5gQF5R3ixc6URdkgd3iPVkSqfP8Db1+iKT0gdPnKE0u8uZfnXT7Uw859eKVLuumv7mjsf+u3mDX946Dnm/pLJ7MIGaUNbcd2uDw889Opzd/HeasBwDipDMZKBwQSTyxxrjYFgb/8kwK/BX8fYrcZrTf4W7PyW/fanfA4MAgdAvJB3hSt1JA53D+ng0FllN99zj9Jv87xx/eVVfV/Y3v6e4n614uoYHERvZKrxbeMxHOUo3G9v2Q1RrCgYo5gVo8OmKDaHUYG4h1kx8LJFD4HFEstsTcy/w+XCbD9mG5k/6DDdFHM8RjrCLcYUYwInFglGHYll+bEsPZbFsiZ2yUOKUoFNasJvN+kLhvvT/QsWLGgR47pgmb3FjrO0BYfZz/wt2LE9MNvvd6TacZQNjlRHf4dnhAfPzIgLwuVxqKOM07e0t4WXsZtuCTc/1r4d18cz7MP6XabrTq1MV8w/k2499Tp7MVH5dG1bUjy+djUUmmqNQyEOVu+0GmXzY6wI0zZ0BzYBm8ziHIw54uTo2GibzY7x74yNihJtNIMJW4NfQMwsHWaAxCeoEruHXYZd4n8oXalQJKUJv8+lL2hJ19qzQGsPP5jfrzWGZ9lb9tn34RB5RGu0luDB0+wmJaO1WTnWule5vL1BTv1Gkke0v6LcwHpd2348/Pja8C/XrsWew1dCZKqpAUfMhCMWC0v4iBXviDVgFxf9XsIvRZIxBpM7zMaYx0TwgG7AXyb+oNMSNDO/Jd0imQ0Wi8EsSzKOy84K3D20UUnXQtXit7dg7NoAYUP4QDA+qzBghqHLGLPxki1b2PRvcuSbNrffLhXLU9ok03Vtp9eGxyj3t+Yq/dsu4X+DX43x1hrvFPHGwa8bZetjOJ8AIyrC6WNixUF7lBIFMVFROMecuO5tdh64QwRuxd9c/uBIOdYxwyH5HZ86pHwH81tZtnWbVQpas60hq9xHZnX830Z5myw7rLJsdcisIoZhN1y6w8A7phDSU/3pC/TWLatasAznXpXWyG5DxKebyOJN5btvKjbT5eHNxjFysXs3y1s3b25bLG8N125tO2wMy+tkV7iFxbV9xGrb2tvaJ0h71uFHqMKm4qrar6+qWFxXN2vryhprjnWi7CaMrXiHIrv4SIGdL5ddcQBxiram+GANNOEnwow4VhvDNsawShOrMzWbjprkbAuLizFZLKaYOIVV2Jl9D/8RgT8Q0jsaiONXhe1zpIo5J07Mn8rX13AxmtqqYmJVdbQSJ6CYk0b/1tPrNks5t9zS3iDlsLbWJuUNqVa5uPWB6+XV4d1sYlst2xU+0rpXGruufTT/8dHZ0ljc0+fs5sO6MykJ4vggm/imgUNtx/ZY/NZ0q2S1AtvDf81gjs2fwPITWEJC921hgVhE/gUt2j6AG6y2AbC+4OCbKne5r+SKd+JHz4jzJQy4LeG18OfMdvwNZg9/+vrtzU/cduuTe8VGwN5nC5kff6VeEr43fDh8JHyPcnf4zZaPw8dZ348+wd+gCmyKbDL10kdKhQHw1c4Bcq9+OCw7PQmeKBylYBxuhokeo1tVFNVt9CQaIQ6XTdEfIDaxb99+iUa+VTSbmKkp0hyMjrZPMpmScOPE2duPj6pnQByLa4p8trNH8qS4JpYSdDpfUdkHKjOqCao0QP1alVSenWDzO1nQlm2rs220Nds+sxmdNqfN8zDLh56QiL0Vk2TBpZCUnjQjSU5K4hsqn9QLsIdwwqZ3bDt8ZttbfHg4xvi1f74FYl91juE9qs8QbRLwY7jYkLrvsAOk84yS6byRzhEj+Kz3YFf3EMvBZZy1JRwX3sMmbA6XLn57SP+3Vi39/V/C98nTw9s+eqD92FZlLltmKDx9Z6r0xVq2+fTXrFcaY8XV4Q+mLlrASp7JXbcud3d4Z3ZbobQgPOjnIHblEO4NvfDzrewRiMEZI/EtOWhXTIrRIimKZDEqsRBregSXhcL3MewIq9EIf7awjyzM0sT3X2sFTqtHcf+NwWXgx1ovw8/D4bgZL1jGp097S+f61hd4IGXUCI8zznCe1uR4XOOrDb16mVtrVGV76y7l+CCWHVrLxjLTTzYPYneubT/SHp6wFiQWwLm+Q+y8PeCOoK2HFBcdHSf1UMBleRjnuR03s6IdCkTzz0fcxcZaMRwLPsf/9oEbL+4D8Rj/YIfPcqFlieUOi7LLwtJlJvld6a5al9zsYn5HukOyyC6HwyVbFIPYnTsXR3rH6l5w6YKOLdrekuoXq5svbu0+Xy/6UDpS1VE9jErHB42xV3gtu3JLq+t9tme/9Rfhu9tPyne2nZZSd8mvXCsPPbVSGRPun7TaaTFY14XnrxZfjz26ZuP32n+Hjv4jYq5v0QQSiUQikUj/gdpD+v8hqc8PSCtJPwp9/q+RXEwikUjn0FP/91LWkkj/UXrtXyPDHBKJRCKRSCQSiUQikUgkEolEIpFIJBKJRCKRSCQSiUQikUgkEolEIpFIJBKJRCKRSCQSiUQikUgkEolEIpFIJBKJRCKRSCQSiUQikUgkEolEIpFIJBKJRCKRSCQSiUQikUgkEolEIpFIJBKJRCKRSCQSiUQikUgkEolEIpFIJBKJRCKRSCQSiUQikUgkEolEIpFIJBKJRCKRSCQSiUQikUgkEolEIpFIJBKJRCKRvosAgOGRjC6BTaQNMCfyNMyBiZG3gaOyn4t8XjJaLw9Y2gQxeloGV2e+0q2MAWt06Wkjpj1YkikWvCqEoXqa4bs36WkJYpQiPS3DYGWynla6lTFAolKtp42Yvl5Pm2CN8l962gyD4UI9bYGRhv562io3ddYTBSmGeXrahunf6eloeb3hdT0dA7P0Mk6M02op0tNdcTrRk62telrpVqYrTme3OJ3dYnNibPYol562sq2dz3bF5tRim1ZQtmh5uTqxoqakdKE6q2jR8rKCqouLqqpLK8rVkcNGjtIKaPfhN6DCcEiBAIzE1DQohYVQBRVQjUcx1GDeeExVQaXwAswpxVQ5DMM7Y6EMpcIszFsEJXivWlwV4bkIS69AL8SS0/C5MiyxHJ9TYSI+X4Ol+ZtUdP5MAR5Z4g1FmOalisVb+PsLMY/XVIY5laJGFS6Dy9FrsOwSrKEc6+Z1FGK5AvFUAeYUYR5vA29Bmai3CnLwuUpxpwZ9IUZRjmV4zYvwzhBRS7l4Q6l4d6XoixV4VShapaKvwueKRMuL9Ai1qAs7I+FlKkUPlIoyC8UbK0UENaLdy/FpflUm2sFbWi36vUiU5307We/NIvGepfq7tL6qEvUt1N9b09knBaLfC/UaVdHLWlu1VhdjiYXd+mWSGJGlIu7FmF8h4i/C3KVn9EU11qC9aylGw0trb1kpeqkEU0vx2ULR4o6xq8K8jvfwfi4Qs2ehiLZKb0e1qKNIn0Xa9XLR5x3zqSuGjnJDRSSL9B7saLsWYQXOBj5KvP9y9LZ3zSNtpq0Q7+e9XibqKcV+KxMlC0Spaoy7QI+oGu9eoY97V1tL9XHoarMWh6rPz3LR4grRtx2tPXtmaSO7Qi9d2lly6Dn6isdRKa67ZrpW0wrRpuUiXlWMbHnnMx3jpIr2rMKrpXpruuZFx7wtwD4oFU/wmTIMN2WrOL5t3c7qNgpVcLHezlJ95o3EWkbCqDNq6P78d9sRakQ/FIr5z9+ypHNGdbTgXPvVInHN51HX/KvAGipFv5XqMyQLW6qN0SLsdb52eH1TxQq8EFMZImcJjrvWoizRg3xO1mBNF4AftVJomOjZM6MYpr/Tr6+9ChFVAaZK8NrfOSur/06N1foYV4iZqNU2DKOpOmeLq0VrK8UIauuho36+EvJEO7V5cLlYYdWds0Dr6641UoOpM3eWIWIHLhVzsEzU0H0ua2tVe3ahXkvHrqitZ20fXCrmVtdOdXm3MSwTLSoSe2DHzsyfqBbjV/U3OcWdbRjyneaDtocX4jN8RQ0R/aV9OmnvHdL5nrNbUCpGfqU+M0v+Tp+t1FtaKnb6MnxPof6597d9z5/R9vyBWH6Q2AWWYn9o/XKu2ks795N/pm+7au/6dKoSK6BGjNzCzs/zc7Wg4+1/G9eF3eYAb4nWlhrxvo5vClViT7lczJ8K7KVy8Tlb8Hdbqs29gjNmlbaXVeiutUpLLxerZnm3bwil+mebVk+J+Lyo/NY5qn2HKddHpqv2jhVSqvcynz8l4pOiVO/nYbufPxAEteHEQy5+Ptc3jO0pT6gHMkA9lHH09WO5+z/G0j0f6XlsPqjH5m/HL8ovfvZC04GaPZV8/r2w++X1oD5x39Gy3SFQmx/ff/TxnqD+/rP9u0J34nOwc//jAV7XwZ4NJ4/N3/EOj3x7yiOnQN1z/66DPIKXDj7cdtB1YOqBXLwDBzYe2rX7KlEm8UBa03z+5IFNx3KfggNp2hv33L8zl8dyYONLroY2fMf7DSfw6jYs6drdsAvf/tIN/I3NDU/9Rov+JWUnHGQHVmFZewPj9x6t2/8xr1OPB9/K3/74DQ0nGxR+f//U3W80bMAn5zzylVbHgU081qNl21N4C3fuw16wd7St8eau6BpO7F7FY2tQeGxPvNbAQH3a8cTHj6taa7p65Hff7ISXcjqi6BqVsz+hDtQ0PPpICp+324M74Xcb+O8QQbgSp+a5YFjCit/q7WCKRCCW/zJRgkoQ8Et55N3Ie+bkzl8uOtZ8w0cwUD6MyUP4k+co/ozpCwMlNy6QHwlG+78vVuNV/1zdytU/nv4jCIIgCIIgCIIgCOKfx1wHq7/vGAiC6MJcxwZ93zH8EKG9iiD+deB62vN9x9AdWt8EQRAEQXxX/lO+N/yntJMgCOI/EXMdG/N9x9Ad+swhCIIgCIIgCIIgCIIgCIIgCIIgCIIgCIIgCIIgCIIgCIIgCIIgCIIgCIIgCIIgCIIgCIIgCIIgCIIgCIIgCIIgCIIgCIIgCIIgCIIgCIIgCIIgCIIgCIIgCIIgCIIgCIIgCIIgCIIgCIIgCIIgCIIgCIIgCIIgCIIgCIIgCIIgCIIgCIIgCIIgCIIgCIIgCIIgCIIg/iEYHjY8EvCQ+FX5SHRFv6O5FSbBFJAzJkzNgfiSgvJF0LusoKYczhflIBIRZVjWjFkq2PVrzbvud9U1H/5yRl2qqGtCZ1n+9o7aEkWOImIzgFm/YiCDESzdnjCBtWzx0gq4SPjUGu5zheevKKqqgWLhZcKrhK9awe9eI/znWI0Ba5GwXgVTZ18x/uZOh3O6hBGZOs8O6AdDsH/SIAOmQg7kQwlUwiqog/WwEW6DbXA/Pse1ActjHSxBP+/QztI4/Xy/dpZVcZblcnmNvFXLs23Tzs5fixiYS9HOCU7t3EPVzonDtHNSmnZOnqKd3T318ycY9RToG5gXuCiQF5gb+CnrGbg48JNAeiAYGBsYFxgfyAhMCGQGJgUmBiYHpgSyAlMD0wLTAzNyrgpkB2YGZgVmB3ICc3Kqcqqxxr5MYg6WxJKZm/Vk4yTJ9pXtpO2vtlO2b2ynba22Nlu7LWyLREO0FC2nnEh5M+WdlPdSvkj5MhAXcAVSAyOym7J3Y9/znuUtGSiitPDxiHrAhmMeeDZwBOYFjgdasV8liLfF2hw4ieNtCSDbkmxJYLS5bQPAZBtk80Gc7X+IMw+oqBJlXdcmhyYJIgooIIgBsLtBwKyAiAq6q2ormCUqBkDEPCYMYxwdw5hGxTDqmHPOOeecxZxzwPgKdeZ4zzqec99a794nC6WD3bt3f9//V9GBOgO46oJ1YeCuq6yrDF66arpq4K2rqasNProoXRT46aJ10VDGcMxwHPwNJw2noJzhg+EDVDB8MppCgNHcaAlGo7XRGioZ7YwOEGp0MhaByt+O0UWOoTzkfqP7R0dpr5MvOcoiOmd554rp3ORY/XVlwVwXoAuQYzXqjGClC9eFg7Wuiq4q2OgidZGg09XR1QE7XV1dXbA3fDR8BAejmdEMHI1WRiuhTJ5NF6ObLM922nAByOhsjISE/3JcCV+Oy/aHx2WpM9fZyXGV0JWQZ66uqyHPHKGLAJ3hhOEE2BltjDqwNzoaHf/Ds/3jndL9L5+Df35+u/8nr9VUHkt8MRV3TKO+PK594XWm0fJdQr71/7jOwlcuW8i3x7frTKCPulHdCKBuU7eBou5Wd4OJekA9AKbqUfUomKmn1FNgrp5Xz4OFekW9ApbqDfUGWKl31btgrT5SH4GN+lx9DrbqG/UN6NQP6gewQwUVsEcLtAAHtEVbcET5AicsikWhCJbAEuCMpbAUuKAv+kJRLIflwBWDMAiKYTAGgxuGYzgUx+pYHUpgBEaAO9bFuuCBsRgLnih/oCQ2wSZQCptjc/DCNtgGvDEVU8EH22N7KI2ZmAm+mIM54Ic9sSeUwb7YF/xxIA6EsjgUh0I5HIWjoDyOxbFQASfiRAjA3/F3CMSZOBOCcC7OhYq4EBeCHpfhMjDgalwNRtyAGyAYt+JWCMFduAsq4X7cD6F4BI9AGJ7EkxCO5/AcVMbLeBmqYD7mQ1W8g3egGj7Eh1Adn+EzqIGv8TXUxPf4HmoREEBtMidziCAbsoFIciAHiCIXcoE6VJyKQzSVpJJQl0pTaYihslQW6lEgBUJ9MpIRGlAYhUEsVaNqEEe1qTY0pGiKhkbUgBqASvIFSI2pMRA1o2bA1Jpag0YplAKNKZ3SoQllUAbEUxfqAgnUg3pAU+pDfaAZ5VIuNKef6WdoQSNpJLSkX+lXaEW/0W/QmqbSVGhDeZQHifQH/QFJtIAWQDItpaWQQqtoFaTSeloPabSFtkBb2kk7oR3to32QTofpMLSnE3QCOtBZOgsd6RJdgk50na5DBt2m25BJD+gBZNFTegqd6RW9gmx6R++gC32mz5DDZmwGXdmaraEb27M9dGdndoYe7MZu0JM92RN6sQ/7QG/2Z3/46UsymIufIBl6CiINHyQfY36QF4V2bFY3ix071B1ix151r9hxSD0kdhxXj4sdZ9QzYsdF9aLYcU29JnbcUm+JHffV+2LHE/WJ2PFSfSl2FKgFYscn9ZPYYYqmYocVWokddmgndhTBImJHMSwmdnigh9jhjd5iRxksI3ZUwApihx71YkclrCR2VMEqYkdNrCl2RGGU2FEP64kdDbGh2MHIYkcCJogdLbGl2JGESWJHW2wrdnTEjmJHZ+wsdnTDbmJHb+wtdvTH/mLHYBwsdgzH4WLHaBwtdozH8WLHZJwsdkzH6WLHbJwtdszH+WLHYlwsdqzAFWLHWlwrdmzCTWLHdtwuduzBPWLHQTwodhzDY2LHaTwtdlzAC2LHVbwqdtzEm2LHPbwndjzGx2LHC3whdrzFt2LHR/wodpiQidhhSZZih450YocTOYkdruQqdriTu9jhJVNYXfIjP7GjPJUXOypSRbEjhELEjspUWeyoQTXEjkiKFDtiKEbsiKM4sUP+iB3xFC92tKAWYkciJYodaZQmdnSgDmJHFmWJHV2pq9jRi3qJHf2on9gxiAaJHcNomNjxC/0idoyjcWLHJJokdkyjaWLHLJoldsyjeWLHIlokdiyn5WLHGlojdmykjWLHNtomduym3WLHATogdhylo2LHKToldpyn82LHFboidtygG2LHXbordjyiR2LHc3oudryhN2LHB/oAXVhhReywYAuxw5ZtxQ5HdhQ7inJRsaMElxA7SnEpscOXfcWOclzuix191C3qFiF+p7pTiN+n7hPiD6uHhfgT6gkh/qx6Voi/pF4S4q+r14X42+ptIf6B+kCIf6o+FeJfqa+E+HfqOyH+s/pZiDdDMyHeGq2FeHu0F+Kd0VmId0M3Id4TPYV4H/QR4v3RX4gPwAAhXgY2IT4UQ4X4qlhViK+FtYT4OlhHiK+P9YX4RthIiNdQE+KbYlMhvhW2EuKTMVmIb4fthPhO2EmIz8ZsIb47dhfif8KfhPgBOECIH4JDhPgROEKIH4NjhPgJOEGIn4JThPgZOEOIn4NzhPg/8U8hfgkuEeJX4kohfh2uE+I342YhfgfuEOL34l4h/hAeEuKP43Eh/gyeEeIv4kUh/hpeE+Jv4S0h/j7eF+Kf4BMh/iW+FOILsECI/4SfhHhTMhXirchKiLcjOyG+CBUR4otRMSHegzyEeG/yFuLLUBkhvgJVEOL1pBfiK1ElIb4KVRHia1JNIT6KooT4elRPiG9IDYV4JhbiEyhBiG9JLYX4JEoS4ttSWyG+I3UU4jtTZyG+G3UT4ntTbyG+P/UX4gfTYCF+OA0X4kfTaCF+PI0X4ifTZCF+Ok0X4mfTbCF+Ps0X4hfTYiF+Ba0Q4tfSWiF+E20S4rfTdiF+D+0R4g/SQSH+GB0T4k/TaSH+Al0Q4q/SVSH+Jt0U4u/RPSH+MT0W4l/QCyH+Lb0V4j/SRyHehE2EeEu2FOJ1rBPindhJiHdlVyHend2FeC/2EuL92E+IL8/lhXjTrxOZ+dtv+5GDOPBe3aBuEAc2qZvEgS3qVnFgu7pd6N+p7hL696h7hPt96n7h/qB6UIg/rB4R4o+px8BOPDkJ9upp9TQ4ii3nwEm9oF4AZ3HmMrioV9Wr4Crm5EMx9aZ6E4qLP3eghHpPvQceYtFD8FQfq4+hlLj0DLzUF+oL8BGjXkNp9a36FvzEq/dQRv2ofoSy6mcEodYETYRXMzQXXi3RUki1RhshVYc6YdQeHYRRJ3QSOp3RReh0RVfh0g2LC5fu6C5EemJJIdILvYRFHywtLPqhH9QSG8tCbSyP5SFSnAyEKKyIFSFazDRCXQzBEKgnfoZBfayMkq9iqUwfWANrQCNxtTaoGImRQGJsNDDGYAw0Fm8bQBOMwzhIEHtVaIqEBM3F4cbQAuMxHlqJyc2gNbbAFpAoPreGJEzEREgRq1MgFdMwDdqK2+nQDjtgB2gvhmdAB8zCLOgknneBDOyKXSFLbO8BnbEX9oIu4nwfyMF+2A+6ifm50B0H4SDoKf7/DL1wGA6DnyQFRkIf/AV/gX6SBb9CfxyH4yBXEuE3GIiTcBIMllyYCkNwGk6DoZIOeTAMZ+EsGCEZ8QeMxHk4D36RpFgAo3ERLoJfJS+WwlhcjsthvKTGKpiAa3ANTJTsWA+TcCNuhCmSIFtgKm7DbTBNcmQnTMfduBvyJE32wUw8gAdgtmTKYZiDR/EozJVkOQHz8BSegj8lX87CAjyP52GRpMwlWIxX8Aoslay5DsvwBt6AFZI4t2El3sW7sFpy5wGswUf4CNZJ+jyF9fgcn8NGyaBXsAnf4BvYIkn0DrbiB/wA2yWPPsMOUkiBXZJKZrCbLMgC9ko2WcM+siVbOCAJZQ8HyZEc4bDklDMcoaJUFI5JWrnBcSpBJeCkZJYnnKJSVArOSHL5wFnyJV84L/nlDxeoHJWDS5JiAXCZgigIrkqWGeAaBVMw5EuihcINCqdwuCW5VhVuU3WqDncl3WrBPYqgCHggGVcHHlJdqguPJenqwxOKpVh4JnnXCJ4LYggvJfU0eEVNqAm8kexrCm+pOTWHd5KAreA9taE28FFyMBk+USqlKiBp2E5RqD21V0wlEzspZpRJmYqFJGO2Ykk5lKNYSz52V2yoJ/VUdJKSPyl21Jf6Kg6SlQMURxpIA5UikphDFGcaSkOVopKbIxRXGkWjFDdJzzFKcRpLYxV3ydAJigdNpIlKSUnSKUop+p1+V7wlT2coPjSTZiq+kqpzFD+aS3MVf8nWP5WytJAWKuUlYZcoFWgZLVMCJWdXKkG0mlYreknbdYqBNtAGJVgyd7MSQltpqxIqybtDCaNdtEupLPm7V6lC+2m/Uk1S+JBSnY7QEaWmZPFxpRadpJNKhCTyGSWSztE5pY7k8kUlmi7TZSVG0vmaUo/yKV9pIBl9S4mlO3RHaShJfV9pRA/poYKS108Uomf0TNEktV8qjek1vVbiJbsLlAR6T++VZpLgn5TmDAxKS8lxU6UVm7O50kbS3EpJZBu2UZIl0+2UFHZgByVNkr2I0pZd2EVJl3wvprTn4lxc6Sgp76F04pJcUsmUrPdWsrg0l1ayJfHLKF24LJdVuhbmvtLtB3vAtzYwUb+1gaPcrzUHcAAAB7Fs2KxnPZiwkY1gyiEcAmYcyqFgzuEcDhZchauAJVfjamDFNbgGWHMtrgU2HMERYMtRHAU6juZosOMYjgF7rs/1wYFjORYcuSE3BCdWWYUiUs4EzqyxBi7chJtAUU7gBHDlZtwMinELbgFu3IpbQXFuw22gBCdxErhzCqeAB6dxGnhyO24HJbk9t4dS3JE7ghdncAZ4cxZngQ9nczaU5hzOAV/uxrIDcA/uAWW4F/cCf/6Jf4Ky3Jf7Qjnuz/2hPOdyLlTgQTwIAngID4FAHspDIYiH83CoyCN5JOj5F/4FDDyGx4CRx/JYCObxPB5C+Df+DSrxJJ4EoTyFp0AY/86/QzhP5+lQmfM4D6rwLJ4FVXkOz4FqPJfnQnWez/OhBi/gBVCTF/EiqMVLeAnU5mW8DCJ4Ba+ASF7FqyCK1/AaqMPreB1E8wbeAHV5E2+CGN7CW6Aeb+NtUJ938A5owLt4F8TyHt4DcbyP90FDPsAHoBEf4kOg8hE+AsjH+BgQn+ATwHyKT4HGZ/gMNOZzfA6a8AW+APF8iS9BAl/hK9CUr/E1aMb5nA/N+SbfhBZ8m29DS77Ld6HVj/ZKQatwzt4pM8RhmRfOymxwXeaAB9L5r6TfP0uXW0tvO0tHe0of+0v3GqRnq0qn1pH+bCRd2VR6MVk6sLDvuku3DZAeGyGdNUH6aYZ00Z/SOyulYzZLn+yV7jguPXFROuGW5P8TyfoC/CRpbiXJXURS2kMSuYykr16StoqkapQkaEOBMEGSMUlSsKMkXjdJt8mSaoMlwUZLWk2XNJovybNCUmaTJMoeSY9jkhQXJBVuSgI8Ftvf0kfx2VLcdRJP3cVJP5m6fvBbry9nZbPs2ntlxz4uu/VF2alvyS79RHboAvWT7M1Wsi8XkT3ZQ/bjMrIX62UfriJ7cJTsvw1l702QfTdJ9tyOst92k722v+yzw2WPHS/763TZW+fLvrpC9tRNsp/ukb30mOyjF2QPvSn752PZO9/iR9k1LWXHdJLd0l12Sj/ZJSvKDllZdsdI2RnjZFWMlx0xUXbDDrITdpVdcJJsgoNkA/xFNr9psuvNkx1vuex2G2Wn2y273FHZ4c7L7nZDdrZHsqu9oQ+yn1nIXuYo+1gJ2cN8Zfv6d2dlo7pN3a0eUI+qp9Tz6hX1hnpXfaQ+V9+oH1BBC7RFRyyKJbAU+mI5DMJgDMfqGIF1MRYRm2BzbIOp2B4zMQd7Yl8ciENxFI7Fifg7zsS5uBCX4WrcgFtxF+7HI3gSz+FlzMc7+BCf4Wt8T0DmZEMO5ELFqSSVprIUSEYKo2pUm6KpAanUmJpRa0qhdMqgLtSDfqM+lEs/00j6laZSHv1BC2gpraL1tIV20j46TCfoLF2i63SbHtBTekXv6DObsTXbszO7sSf7sP+/PSubZCrfIxP4MZm2L8hkfVOm6McyMb9VP8pMbCnzr5PMuu4y1/rJDFtR5tXKMptGyhwaJ+NAvMyXiTJLFs6NXWVG7Cfz4DCZ/cbJnDdNZrp5Mr8tl1lto8xlu2UGOyrz1nmZrW7IHPVIZqY3+EGmIguZgBxl2ikhk42vTDFBMrGEy3QSIZNIrEwdTWTCaCPTRHuZHHJkSpgo08FAmQRGSev/Lq0+Vxp8mbT1BmnmXdLCR6Rxz0m75kuTPpTWfE3vpRfNpQMdpO+KS7eV5rL/9qxsULequ9T96hH1pHpOvazmq3fUh+oz9bX6HgHN0QYd0AWLY0ksjWUxEI0YhtWwNkZjA1SxMTbD1piC6ZiBXbAH9sFc/BlH4q/4G07FPPwDF+BSXIXrcQvuxH14GE/gWbyE1/E2PsCn+Arf4WcyI2uyJ2dyI0/yIX8KIAOFUlWqRXWoPjUijZpSK0qmdtSJsqk7TaCfaAANoRE0hqbQDJpDf9ISWknraDPtoL10iI7TGbpI1+gW3acn9JIK6BObshXbcREuxh7szWV++Nv0XV86PEi62yidHSpdXUU6uoZ0c4R0crR0cX3p4IbSvSSd20S6tpl0bCvp1iTp1DTp0vbSoRnSndnSmd2kK3tJR/aVbsyVThwiXThcOvAX6b6x0nm/SddNkY6bLt02SzptrnTZAumwJdJdK6Sz1khXbZCO2iLdtEM6aY900QHpoCPSPSekc85I11yQjrki3ZIvnXJbmuRHv7UsfGUVOJArsoGDuRKHcWWuytW5JtfmSK7DdbkeN+A4bsTIzI05nptyc27JrTmRkzmV23I6d+BOnMmduQt35e7ck3tzH+7HA3ggD+afeRiP4FE8mn/lcTyBJ/JknsrTeAbP5Nn8B8/jP3khL+alvJxX8mpey+t5I2/mrbydd/Ju3sv7+SAf5qN8nE/yaT7L5/kiX+arfJ1v8C2+86NXprjBML7H9/kBP+RH/Jif8FN+xs/5Bb/kV/ya3/BbLuB3/J4/8Ef+xJ810BTNRDPVzDRzzUKz1Kw0a81Gs9V0mp1mrzlojpqTVkRz1ly0opqrVkxz04prJTR3zUPz1EpqpTQvzVvz0UprvpqfVkbz18pq5bTyWgUtQAvUgrSKml4zaEYtWAvRKmmhWpgWrlXWqmhVtWpada2GVlOrpdXWIrRILUqro0VrdbUYrZ5WX2ugxWpxWkOtkaZqqJGMdJrWWGuixWsJWlOtmdZca6G11FpprbU2WqKWpCVrKVqqlqa11dpp6Vp7rYPWUeukZWiZWpbW+QfzxNfPdm2+fT7+1+e8AGULP+VN6ZjR9tvP1l2+/myQb+cGmdmpATGJGW3//qHwr/SA2MLPuOHbZ75fHstzifzr8vUhPefqcz1nWViXHxIz5I2dYmmSl+s5Tq4abaIoBlu9tYV5BXtTkxLmoE+0sKlgoZgpuWEmilke61Ef8N01HrNK9veAal++GkESdIFM6AipkCPfNQq/9N7fPZiZy+e5V2aN3RHSJyula87mgaMOeq1v/y4v1y1Fn2u2U59rujDP1EQxMXEOlkNclOx650Z045UeXw54kd7u76NVzOW4un85TNPGZhbOJo3Z4Kx3Krxg5WwTn9ilXXpG25zMDIOj3r7wSktnS0pN6ZSZkWIoqfcovMbGuWhcenJ2ZpfMtByvyMzsrMzsxJx0+R/e+lKFt5s6u/3jdi29U2og5yR2yvJSI2vrSxazM4TqK+mDDWGGYGNwM7kYpg//+6J+wMr/kSOz09sW3m7rbBbXSCWDv97v68WSGZHpWe1Ss72iuI5XHW5YJVIfHRIYrA8PDYyqEx1u8NOX/vqKPP7lK+LU7G7pyan6XMXn+zOsmINpruIAcr2NSa6iwAzvy4MuZdc7GP82c0RMkomjXb8mzqentu58dkC0OYz/065W7O1Nhz42LZFaxfdW6Wcme6cO6r+Dls7aPuL5qLDGByJCd/Y92SrZ0O36oE9PDaHpEHLrZmy1J/NogcmvuxpktOvxdtnUtc3jr/ufKT/V8s+CFgXvwlxqtxyasizrRtO5CXuGBM5Pb7fm8cRbR9pu8rl4f0bDlaeTxnTfUjR2+4IGRSLDLrjbY0HfmVMfKy+zqjl1Tn4290S3FnHLul4IWfjMZnuxTTNOP3lTruyCCXd8LqWdSK1Z/e7K35euuTp18uHWe3c1G1ml6YQBi34ZUfZDn7XdSx4c0eXEGM+ufUbdXVZkbU5ISfvDn6t6VR7xYKKJqXg0O1exljNirveUU+ppb+Zq5uIdH6A8Gqi2S7+8PS594aiFjcaHJX1hyLO0mZvetb9L6ZC35yk6y+ZRrffd3q+ssGxnpZUOeq3wDqXM4vQN9PXy6ubVGRLZLicnq0rFisnZHYM6/fU+BSVndqqY1SG98NqKWdmZKV2Tc7pU/PttLHwXv7yJQmWQ3EWfYGElYpqbWyqKWay+vj7mr8t6kyHVvj1B9+7d/9UTpGb/m0fO0TsXHq+fmU5v89dDmlr9k5CmhZREhw5vZjL5Wly3DKvqLdMjgq5ea2A3ZYRmuFfgsO1a/soRzidSuteLC9/SYkjq8IKrtQMyaMHOl3Xflp9b0tujSey11XVp1fLau2wTnHz6rgw1v5HjPrdctPeAxz2PLVha5e2lYm+aNRg0vYGlsd3bBr8n0OBJteIDtjV4MfG5zQOTcVHtth+/t6Bkx6vhKUar3a3vFGtujFfX7vkp8ol11gEfXccuk13aN/HpXTDHau69pp1d75v2dTl5IG7jDc9ty1fv/Tg5fsF4++SZhlZvfzt0wLZh3K2Xxvi3i6vuWPzxpmnsw6ixnw+ObTm8WOeYz/7l+tY9bpfbrM3O5IeZmaU6Bd5f9HG58x+zgx8u/ax80udaWEqMPfkaYzaJru1CvqSXxz+nV+svsWBjPbbMsHHPA1KU4q6m8l4YiuuL/Zcrrf9+qwyB+gpfPfb9h8eUmSkhIe9delp6cmJOqlftrjntMrPTc3oWppQ+TB8isWSsFKyvLCllNHy5GKwvvPj/Lz7/Y9DM7Lj86sWYseV/6hBU/Prm/Bu7J2NpdfGRy24NfR0eH593PHZxjt7L6YHlaW1C0Xrj3SPGLpnUQl/mAnS423vzw2GWDm/szSY9HXao1MFg35+nPX/Z1iPgQ+87Qz3v32k4e+b20nxg1Ls6R62PtVp6bFmE2ayCuR3HtT1b9lI0Lxty7FbZ6CD/RUMaNSbdTdOA9+3HjNFn/PyiqX7au75nJq686z2x79sTzi+s1nInWlVnzIwYqF83zcm/XNr8iTdPWgyoP6tg0Dynui7WuTMGPWrc45MyxVO1GgyO+uhHa6+Ujt64K1CbsbRkj9qG7oemXq06cNzMRJPVnnbLP7yZukI54tNA+1xgvnOHl+1fQbNQzsg8vUOheM6K8tnMXG8q/3yXPP8yWAo7wtPBzEz4G6J3tLD+VtRFlcJrQD9g0tdkGjBGP2BUfxf7RbltajXxn3jLz/lD+es2PKHpzTkzk+ck/o/jmevYc7HrzPp5fyyO7ZLw0tI5KFWvfo3Eevq6+jp5kXm1h9T870fi3zdnyzMWZtmXONS+i8MYfbQ+6rs4DP+/icPC1xH59VH/m1Eo59px4vCdLUyjQi/fW7W4+8UjPTFOWR6U07l5J53zwiNbeo9eF3SqyKyRnZLWxZscbOjlrE6+3KtWfvzGpQlTPK57KkMWbezxfMSxh1WVx/lbRtuY7xsVk/+Ui15utHDszTuj2p/uv/32+OcWFQeb3vu1vK9P1vvXH272mBxk98YyP2uTW8Npv3SwyZ6wbmbl39sG7kb7+0ktarpOGuFVM9+yhLHgkKF+N0P1Ctm2++5nVf882Mb56g6bxF+enl1X7EHDEf12V6rQavbWB5v62Eb0PsXZ3o/1Bzb2SG3RXClm42J/4oLLpFfV1qclrAyseKdg8JBD2OTutKzxHRdVjj31uufWBW69kso9mTW1XIhF9xJJ+6uX7FQq96nt3oCNRyNX3ip42Gf1jTnzcyqta7i7c+kiZbrZVqORnZtFR7psWrlyWVzbfTMiPvfv6d1/elF92t2IIq1K7Jvu430s8l6FextfxhwKOHXO2D+2TPkY39bN7jd5MvfK5GkHqmRuHuCfY+H0uJv31qm52/21NcvbVx82s1viqoyZznO3Lqj7tEjmx+HGjis+XcV9I0vvT9s8zfPnIikm1QOXNh297qb3rdXLDiSv6qGZn6odpC4av+yPHgtX5v3WtcT5sT87d/WpaJxvlZHXfKTf1rwngw54n3lQstH+KY/rXXujpGYOs+2zL33f7Yz78yYeMZT7bL+7eYtzce4zz72rOL1mUGPXDvudZ3+UKnCUKnj73USbUnfQ+CEXaxcP/9IJKd9TYyud0Pd/JJLL6st8FbPU97enpHpxetsMedTCUPYy6g2GL4NtJYPeGG6UmggJ/TrYGv6+qB8w8H9jsP12d9Mf3P0/9se4oAvnbteYdN7dIaV13PyEOJeqb3oN+lDNyVDlybAd9QNr/xHT8nKR6U8WppUNOd2nfOPesXHDn297tuIkFR2xZqXVuhGq7eV+79tb511qa7br2dVNXd28am3pETC5yeXHr4Iu14goaFZKv0RxGb4sdzXm5XdK/fTGzWaUvlNclXm7X4QGPm4dkDcWlnvE5h9889nXr9/Z0dsuT8w4sqLYmr0eUfN8D/QdcPu2Yb3r+ZY1jrd7vOVwzLEhAUsu2Xtv3zZ852P3cU2dX++p2j/bbSi/X7hVXyOv+2YaurXXubvLxi2e6T7yVN+5jqrjluv7PFLe/Rz0flfdNNdtg3qHLm29YXBURxOHwGvL7N1n1fD7qz/6yRnp/XUS9SucRP9OK9O/17dYK+W7Pklo0tns6eaNwa8Gtjt0p3/0m/ilJ3z0WHizk5nMknMkQP/5vQrWGwovmjuXDzaEhhgrhFRODU8OTgsONAZXqhwYEpYWGpgYnJQaGJocnhwSVsmQHJIU+l+G45iMlFuq+cncBcXCwnxWd5p/oKvJhB8Px/8yrDOzunwpA6FFsBaohedCnFsX/hWoDwvUh39pg8Tv2qCxXvbY79qgzn98gr8K4d88RY5e93dZm+jhn+z+0glD83UpkZ6XWywZWXWw7+pptc5sTlvUPL7S+Zd3q2yrM9kx7uKqMTcPdsMB1k9Xjot7+arIzc0x/i4LMgPPdhh4as+8nh9O+rlHH+hmMtS4RNXemRbkKrtHrEx2q598a/knl6q6nSef919Xze9emxVPG6+/VKm+eeCDjLbJmypt2eRbsO3ynV1PTH1UF/PVh2as2l9Wed18zVarkJB5i6v6vO9ebG35QQ06VNWy/7ibmBExZvTIrT03edxZ8JvHjKKhhmolE1r7WlebY51UKtj2zMdjEw422nAheP2YNyPz7K+nTZg/ucBsfN2upzdMjJpuczvNXLld6dWlo6MnpmY+fLGbf0qd3dV7dIvADeMiXsT/Odtppn8DXLc2qr3+c06ZnF3D8nvtireak3sta8QlZ/8G5vGrhpf+qGRPaqJ3mLFImbq0TlnfCl6vFumr6t+YlW631aJkQ71NdZMpPZZ7WsxrWH1muWKh7sdHvF7ySNkQMoZjXA+bJUU/s+l7y+x6+UEu4/bPPnG3wg2/X5c51ItbvyDe/vHrgwNu5k8y222XX+TzvtFTijY3+z/FmXk8VP0ex4+x73sJMbKN/YyokV3IPLJWyNJkN2TsWSJjuLayPtYURqHIXg/JLtJqX4sxmR4UT7KUK+oeug9ude997h/31R/n9Tq/33n9zu8s38/v/ft8v+sFBrl/f2+o/GgwguKRqMUayQQ8uhhaa1DiOf2wpOuwrlM+KNAIArauRFMZBuI4apn1xEuCr2n6/Mu33IEBV3BcVLb4rHBu+DB6DOuqaDY1aROe3rKxV7a0ThXT4EyudlMqIPkUP8dW2xfvfcnzBgQJ9CEggdbhT3vAlty3hQLqb1EQEft/WWoVQfDr2ir1VzZpO1RAQlYCwoCyylcjcWiriQQ3mz+dWgTY9zyAbfIABvEA0lzpwpovh6B82QiuhMBhpFS/+JuVSJ6OgLTHrLVpSR0dip8GXR/eziI0ftijk2uEeQHVlk1X2aUySMWD1OmPYw12irmYdlbsXEUu+uqs25k+Us6JaibZ9orRWzLlIYwVwxmnH5/lp511OT+jaC7BpTBdymDaXaNbazfyQJ46oNRt6Ynn0hFb4p5l/fpJlNNtnJNyUFG+I7tcv9avq1MT9KyDtsGFaKlp1qZ87sCmNPV3n6ZkrDmEjSwQBSG+k1xHatFnRubnj6ZEjl6ovhAtMKpRddluJs4kin+RqHCakqwqV37QqqNW47Nifw21elV1RSrqYt81vOyKsUWKiLJ4uwrOKfxE/VX2sn2iUU+W66mjEz5iFnrMmy+nxTS0iPiLY/gQvz2VRKDEs1R+OdQdWpVaLihafMtlzl7YnYxAX8PEvhK36xcx1DB/cNdSU4x6oTfERmFQdMrbjt1MP7BmFSA33IYRMC9aeGsaBQZOGU6rENlnRdENfHW6oXqU1nbfkEnfaTFSs352x7s2QcsXkQlzRmiwuDSRNGeTV7E+XunyqjUz4sL80LzhNFqqmBtRVBzmiv893iEIU60QNWx51bY5EIF4P+/ZjkiSTdI6bNJK/ptu3APG4x0DhUcV/NM/4laD4Fay3HZn069omByMGquM3TuRa7ycUdmgn38uq29yKPbytp+ah3g4+wNLtAPAH8Jo3/YAHhgNixATcAIIAByAo4D2v3qt74zabsz5yh2BIZOP3uOhNSa/KX6I7BWNUwKtv2JuM6Nqkm+UbxiN/p9yQJBuIdVCYt0mEQY8iFFU3ILdmV2wMwdNQeNdsNP5a9bnP9zfH4zI23x4OE1EJhiRBkakbH8keWpomwhq/jkdjGrPwf/GVicvRz/ozbCe9r7Bjt5+8m7+nqDW9g1goJKQInw/cBxwBlwBe+AcgAG8t5LMWMARCIZafoA/1L+ZcPaEDhx0Jg/f/yP6ui5GF2ZNngzml+8f8Xc9kMOcwUl2TM3WyQjrC2ZJbnXGyMtqrLb79npGfm7SnGF6rNp87Nb1JewLx+YDyoWZds5RyWGX9E1PjbCkhvbxGwouqelcMu+p3PCY0qCXl8r5XV2gcODu/sA0lVezTo901YNCRJe4w4qS/SMTlp9IwPSl2+I57t+4RcuSM++25iafni+tKe1hhXYUZsTirLMyKJHLLUlL+jIT66o9jcrvcOLlrysk53vGl9gqshGZWUZs6syLDHFDwu2KfK8WOuSe2+TeQaswdTK1dZaVv64efcEba6ZnhVL0keQPr1qWXJ2QPQLHZlWfjnPDeRXX+rdr0dIVUUkjNAia3EYuzC01RivkpHBBL94wveLzr7Wkna+325k7RLfvdzyUGU0aW1pd3EO8Ikl+VpjZ84edo/aUDf3VGA26QLpeuqoAYZ4me/u7Cy87BWiaSNoP2RB/TDgrzGV+INpmjABDRP3G00uZhYyGBhzZeOEeQKqjKqdQUy9QSLmzr6AgLyTkwJpBunDpp2Oi+JXc1WaPWsPMV28Dgvjn3hzODuYz/DJUI+oW8HvF2vqlt8z4N1jVinVwnuZ4IokU4OmYot57zcLYpBlveYAYxKkoEvJOm6lK89PNpzfsWomxOZY+FsYGei06j3LO2zDhDTw2gvNaGz093R+Z+3Gzhpg+QxJoKkECzW0YFRUYkf6zwfVjS7hTK8mPeLC5+PwziBmpkSy7CzHQU+y0mJFs4O6rvKDozkAaJLS0kdxXbnSVpr+5+RieKp7s5WPMercRdNo1hAVpAZ7Ml8YjAKMtYflCEtus5bhAcoIDJyGhbYrOFeq3h87cgGCiBF7s3yr7ZLC3l6uvvbdbsMI3bKYhUAEGCWNP/EkMKvoC8dyWn25jrDVRLEqjJM8gjMTd1TunNgLgFB6HswHEwSmxYJOYfVwPxQ31eJPLGPeclQtQU4vDvj8WgEnr90N/RNUNOFANaU/1ilj5MWd1Rzfxx1VmhDSUGVSXwTPVv9yUmS3jdEpQR6vbuz/Dg2vPwsnk9669rswTN6W7WOUuXl89JDXyXIi/cpCX0+1BT0xvTHTDTA9Fi7Uny7E5rUN5bm2mzulOlnUfZSLZOEw3z3mEWsvlCTJJeEUvt35O5IqprmrgHjGJ2Vo//iyrgsfRJXHlqCS+DyX4uSD4fZ5jJ850z3V0mwmw1gqpYpPuaPP3MNzJNVtTQREJMAS0PRHb+Ud0SAKMF+ri3ArNxJ+WnP1x4W1XTNqBfLtDknmngEgFTb59hRbJvlUkQ4EoJLRJBEHr7yKS3rH7qBfZHouT6hJVTpHCtLEPhHyTRtuMFZ9fykczOek/ezsXSUhdvL7CsCGAu7eccOpZH+WaRV3shXgSqh1VKLzKYP6FqzCiSpKRbONrep8uKTXm8/E5Tdv3a5p1x4KehiGiq0zp/S6OUYrMsJThxfUP1KsDxtwVynvjDrBYCpOGrU/hGHpcY7UTFV8If9xPeUtp3Tf//B1+6Gm5FMd5QbX6xDO2oW1NVeIYGUTEY48viWpRUfcSbMnxjYQ6P2qvJv19cFa8M99KJ2/LjJSN2hG7KxljuHg/Npjc0hf7Cz2UDuGu4ocf3A0jS8Zniib14dZFhh2s5sMZnFxYEx8+JW7BnKkgvDTioPvUzGIox4YeRdX61xQ6u3G8DwD8A8jBcjANCmVuZHN0cmVhbQ0KZW5kb2JqDQoyNiAwIG9iag0KWyAwWyA2NjNdICAzWyAzNTJdIF0gDQplbmRvYmoNCjI3IDAgb2JqDQpbIDM1Ml0gDQplbmRvYmoNCjI4IDAgb2JqDQo8PC9UeXBlL1hSZWYvU2l6ZSAyOC9XWyAxIDQgMl0gL1Jvb3QgMSAwIFIvSW5mbyAxMyAwIFIvSURbPEJCRTJFN0NEMzg4RjhDNERCQzg0QzI0QjA4NjNGMUYyPjxCQkUyRTdDRDM4OEY4QzREQkM4NEMyNEIwODYzRjFGMj5dIC9GaWx0ZXIvRmxhdGVEZWNvZGUvTGVuZ3RoIDEwNj4+DQpzdHJlYW0NCnicY2AAgv//GYGkIAMDiKqFUFvBFONeMMX0Akwxy0KoxRBqP5hiSYRQq8AU63wwxeYDptgdgXqBhokwsEIoNgjFDqFYIBRUCQdQA4cdjMcMoRghFBNQjvM32EzudDAVFQChKhkYAMJUDPgNCmVuZHN0cmVhbQ0KZW5kb2JqDQp4cmVmDQowIDI5DQowMDAwMDAwMDE0IDY1NTM1IGYNCjAwMDAwMDAwMTcgMDAwMDAgbg0KMDAwMDAwMDEyNSAwMDAwMCBuDQowMDAwMDAwMTgxIDAwMDAwIG4NCjAwMDAwMDA0NDUgMDAwMDAgbg0KMDAwMDAwMDc0NCAwMDAwMCBuDQowMDAwMDAwNzk3IDAwMDAwIG4NCjAwMDAwMDA5MzEgMDAwMDAgbg0KMDAwMDAwMDk1OSAwMDAwMCBuDQowMDAwMDAxMTIxIDAwMDAwIG4NCjAwMDAwMDExOTQgMDAwMDAgbg0KMDAwMDAwMTQzOSAwMDAwMCBuDQowMDAwMDAxNjEyIDAwMDAwIG4NCjAwMDAwMDE4NTcgMDAwMDAgbg0KMDAwMDAwMDAxNSA2NTUzNSBmDQowMDAwMDAwMDE2IDY1NTM1IGYNCjAwMDAwMDAwMTcgNjU1MzUgZg0KMDAwMDAwMDAxOCA2NTUzNSBmDQowMDAwMDAwMDE5IDY1NTM1IGYNCjAwMDAwMDAwMjAgNjU1MzUgZg0KMDAwMDAwMDAyMSA2NTUzNSBmDQowMDAwMDAwMDIyIDY1NTM1IGYNCjAwMDAwMDAwMjMgNjU1MzUgZg0KMDAwMDAwMDAwMCA2NTUzNSBmDQowMDAwMDAyNTU1IDAwMDAwIG4NCjAwMDAwMDI5MTkgMDAwMDAgbg0KMDAwMDAyMzEyMCAwMDAwMCBuDQowMDAwMDIzMTYxIDAwMDAwIG4NCjAwMDAwMjMxODggMDAwMDAgbg0KdHJhaWxlcg0KPDwvU2l6ZSAyOS9Sb290IDEgMCBSL0luZm8gMTMgMCBSL0lEWzxCQkUyRTdDRDM4OEY4QzREQkM4NEMyNEIwODYzRjFGMj48QkJFMkU3Q0QzODhGOEM0REJDODRDMjRCMDg2M0YxRjI+XSA+Pg0Kc3RhcnR4cmVmDQoyMzQ5NQ0KJSVFT0YNCnhyZWYNCjAgMA0KdHJhaWxlcg0KPDwvU2l6ZSAyOS9Sb290IDEgMCBSL0luZm8gMTMgMCBSL0lEWzxCQkUyRTdDRDM4OEY4QzREQkM4NEMyNEIwODYzRjFGMj48QkJFMkU3Q0QzODhGOEM0REJDODRDMjRCMDg2M0YxRjI+XSAvUHJldiAyMzQ5NS9YUmVmU3RtIDIzMTg4Pj4NCnN0YXJ0eHJlZg0KMjQyMzINCiUlRU9G",

"title": "DTC"

}]

},

"request": {

"method": "POST",

"url": "DiagnosticReport"

}

}, {

"fullUrl": "urn:uuid:60b84035-6476-4c94-b917-9db1ef7c04fc",

"resource": {

"resourceType": "Specimen",

"meta": {

"profile": ["http://211.253.251.80:19080/FHIR/StructureDefinition/snubh-specimen-genetics"]

},

"extension": [{

"url": "http://211.253.251.80:19081/FHIR/StructureDefinition/extension-specimen-orderDate",

"valueDateTime": "2017-06-20T11:01:00+09:00"

}, {

"url": "http://211.253.251.80:19081/FHIR/StructureDefinition/extension-specimen-speciesLarge",

"valueString": "Human"

}]

},

"request": {

"method": "POST",

"url": "Specimen"

}

}, {

"fullUrl": "urn:uuid:8da12224-d87d-428c-8033-f7f3fe5d738a",

"resource": {

"resourceType": "Observation",

"meta": {

"profile": ["http://211.253.251.80:19080/FHIR/StructureDefinition/snubh-observation-genetics"]

},

"extension": [{

"url": "http://hl7.org/fhir/StructureDefinition/observation-geneticsGene",

"valueCodeableConcept": {

"coding": [{

"display": "USP1"

}],

"text": "USP1"

}

}, {

"url": "http://hl7.org/fhir/StructureDefinition/observation-geneticsDNASequenceVariantType",

"valueCodeableConcept": {

"coding": [{

"system": "http://vernomen.hgvs.org",

"code": "HGVS.c:c.*144T>C",

"display": "HGVS.c:c.*144T>C"

}],

"text": "HGVS.c:c.*144T>C"

}

}, {

"url": "http://hl7.org/fhir/StructureDefinition/observation-geneticsGene",

"valueCodeableConcept": {

"coding": [{

"display": "USP1"

}],

"text": "USP1"

}

}, {

"url": "http://hl7.org/fhir/StructureDefinition/observation-geneticsDNASequenceVariantType",

"valueCodeableConcept": {

"coding": [{

"system": "http://vernomen.hgvs.org",

"code": "HGVS.c:c.*144T>C",

"display": "HGVS.c:c.*144T>C"

}],

"text": "HGVS.c:c.*144T>C"

}

}, {

"url": "http://hl7.org/fhir/StructureDefinition/observation-geneticsGene",

"valueCodeableConcept": {

"coding": [{

"display": "USP1"

}],

"text": "USP1"

}

}, {

"url": "http://hl7.org/fhir/StructureDefinition/observation-geneticsDNASequenceVariantType",

"valueCodeableConcept": {

"coding": [{

"system": "http://vernomen.hgvs.org",

"code": "HGVS.c:c.*144T>C",

"display": "HGVS.c:c.*144T>C"

}],

"text": "HGVS.c:c.*144T>C"

}

}, {

"url": "http://hl7.org/fhir/StructureDefinition/observation-geneticsGene",

"valueCodeableConcept": {

"coding": [{

"display": "DOCK7"

}],

"text": "DOCK7"

}

}, {

"url": "http://hl7.org/fhir/StructureDefinition/observation-geneticsDNASequenceVariantType",

"valueCodeableConcept": {

"coding": [{

"system": "http://vernomen.hgvs.org",

"code": "HGVS.c:c.*6494A>G",

"display": "HGVS.c:c.*6494A>G"

}],

"text": "HGVS.c:c.*6494A>G"

}

}, {

"url": "http://hl7.org/fhir/StructureDefinition/observation-geneticsGene",

"valueCodeableConcept": {

"coding": [{

"display": "DOCK7"

}],

"text": "DOCK7"

}

}, {

"url": "http://hl7.org/fhir/StructureDefinition/observation-geneticsDNASequenceVariantType",

"valueCodeableConcept": {

"coding": [{

"system": "http://vernomen.hgvs.org",

"code": "HGVS.c:c.*6407A>G",

"display": "HGVS.c:c.*6407A>G"

}],

"text": "HGVS.c:c.*6407A>G"

}

}, {

"url": "http://hl7.org/fhir/StructureDefinition/observation-geneticsGene",

"valueCodeableConcept": {

"coding": [{

"display": "DOCK7"

}],

"text": "DOCK7"

}

}, {

"url": "http://hl7.org/fhir/StructureDefinition/observation-geneticsDNASequenceVariantType",

"valueCodeableConcept": {

"coding": [{

"system": "http://vernomen.hgvs.org",

"code": "HGVS.c:c.*6401A>G",

"display": "HGVS.c:c.*6401A>G"

}],

"text": "HGVS.c:c.*6401A>G"

}

}, {

"url": "http://hl7.org/fhir/StructureDefinition/observation-geneticsGene",

"valueCodeableConcept": {

"coding": [{

"display": "DOCK7"

}],

"text": "DOCK7"

}

}, {

"url": "http://hl7.org/fhir/StructureDefinition/observation-geneticsDNASequenceVariantType",

"valueCodeableConcept": {

"coding": [{

"system": "http://vernomen.hgvs.org",

"code": "HGVS.c:c.*6434A>G",

"display": "HGVS.c:c.*6434A>G"

}],

"text": "HGVS.c:c.*6434A>G"

}

}, {

"url": "http://hl7.org/fhir/StructureDefinition/observation-geneticsGene",

"valueCodeableConcept": {

"coding": [{

"display": "USP1"

}],

"text": "USP1"

}

}, {

"url": "http://hl7.org/fhir/StructureDefinition/observation-geneticsDNASequenceVariantType",

"valueCodeableConcept": {

"coding": [{

"system": "http://vernomen.hgvs.org",

"code": "HGVS.c:c.*2686T>G",

"display": "HGVS.c:c.*2686T>G"

}],

"text": "HGVS.c:c.*2686T>G"

}

}, {

"url": "http://hl7.org/fhir/StructureDefinition/observation-geneticsGene",

"valueCodeableConcept": {

"coding": [{

"display": "DOCK7"

}],

"text": "DOCK7"

}

}, {

"url": "http://hl7.org/fhir/StructureDefinition/observation-geneticsDNASequenceVariantType",

"valueCodeableConcept": {

"coding": [{

"system": "http://vernomen.hgvs.org",

"code": "HGVS.c:c.*6494A>C",

"display": "HGVS.c:c.*6494A>C"

}],

"text": "HGVS.c:c.*6494A>C"

}

}, {

"url": "http://hl7.org/fhir/StructureDefinition/observation-geneticsGene",

"valueCodeableConcept": {

"coding": [{

"display": "USP1"

}],

"text": "USP1"

}

}, {

"url": "http://hl7.org/fhir/StructureDefinition/observation-geneticsDNASequenceVariantType",

"valueCodeableConcept": {

"coding": [{

"system": "http://vernomen.hgvs.org",

"code": "HGVS.c:c.*2591T>G",

"display": "HGVS.c:c.*2591T>G"

}],

"text": "HGVS.c:c.*2591T>G"

}

}, {

"url": "http://hl7.org/fhir/StructureDefinition/observation-geneticsGene",

"valueCodeableConcept": {

"coding": [{

"display": "USP1"

}],

"text": "USP1"

}

}, {

"url": "http://hl7.org/fhir/StructureDefinition/observation-geneticsDNASequenceVariantType",

"valueCodeableConcept": {

"coding": [{

"system": "http://vernomen.hgvs.org",

"code": "HGVS.c:c.*3116T>G",

"display": "HGVS.c:c.*3116T>G"

}],

"text": "HGVS.c:c.*3116T>G"

}

}, {

"url": "http://hl7.org/fhir/StructureDefinition/observation-geneticsGene",

"valueCodeableConcept": {

"coding": [{

"display": "DOCK7"

}],

"text": "DOCK7"

}

}, {

"url": "http://hl7.org/fhir/StructureDefinition/observation-geneticsDNASequenceVariantType",

"valueCodeableConcept": {

"coding": [{

"system": "http://vernomen.hgvs.org",

"code": "HGVS.c:c.*6407A>C",

"display": "HGVS.c:c.*6407A>C"

}],

"text": "HGVS.c:c.*6407A>C"

}

}, {

"url": "http://hl7.org/fhir/StructureDefinition/observation-geneticsGene",

"valueCodeableConcept": {

"coding": [{

"display": "DOCK7"

}],

"text": "DOCK7"

}

}, {

"url": "http://hl7.org/fhir/StructureDefinition/observation-geneticsDNASequenceVariantType",

"valueCodeableConcept": {

"coding": [{

"system": "http://vernomen.hgvs.org",

"code": "HGVS.c:c.*6401A>C",

"display": "HGVS.c:c.*6401A>C"

}],

"text": "HGVS.c:c.*6401A>C"

}

}, {

"url": "http://hl7.org/fhir/StructureDefinition/observation-geneticsGene",

"valueCodeableConcept": {

"coding": [{

"display": "DOCK7"

}],

"text": "DOCK7"

}

}, {

"url": "http://hl7.org/fhir/StructureDefinition/observation-geneticsDNASequenceVariantType",

"valueCodeableConcept": {

"coding": [{

"system": "http://vernomen.hgvs.org",

"code": "HGVS.c:c.*6434A>C",

"display": "HGVS.c:c.*6434A>C"

}],

"text": "HGVS.c:c.*6434A>C"

}

}, {

"url": "http://hl7.org/fhir/StructureDefinition/observation-geneticsGene",

"valueCodeableConcept": {

"coding": [{

"display": "USP1-DOCK7"

}],

"text": "USP1-DOCK7"

}

}, {

"url": "http://hl7.org/fhir/StructureDefinition/observation-geneticsGene",

"valueCodeableConcept": {

"coding": [{

"display": "USP1"

}],

"text": "USP1"

}

}, {

"url": "http://hl7.org/fhir/StructureDefinition/observation-geneticsDNASequenceVariantType",

"valueCodeableConcept": {

"coding": [{

"system": "http://vernomen.hgvs.org",

"code": "HGVS.c:c.*2686G>A",

"display": "HGVS.c:c.*2686G>A"

}],

"text": "HGVS.c:c.*2686G>A"

}

}, {

"url": "http://hl7.org/fhir/StructureDefinition/observation-geneticsGene",

"valueCodeableConcept": {

"coding": [{

"display": "DOCK7"

}],

"text": "DOCK7"

}

}, {

"url": "http://hl7.org/fhir/StructureDefinition/observation-geneticsDNASequenceVariantType",

"valueCodeableConcept": {

"coding": [{

"system": "http://vernomen.hgvs.org",

"code": "HGVS.c:c.*6494C>T",

"display": "HGVS.c:c.*6494C>T"

}],

"text": "HGVS.c:c.*6494C>T"

}

}, {

"url": "http://hl7.org/fhir/StructureDefinition/observation-geneticsGene",

"valueCodeableConcept": {

"coding": [{

"display": "USP1"

}],

"text": "USP1"

}

}, {

"url": "http://hl7.org/fhir/StructureDefinition/observation-geneticsDNASequenceVariantType",

"valueCodeableConcept": {

"coding": [{

"system": "http://vernomen.hgvs.org",

"code": "HGVS.c:c.*2591G>A",

"display": "HGVS.c:c.*2591G>A"

}],

"text": "HGVS.c:c.*2591G>A"

}

}, {

"url": "http://hl7.org/fhir/StructureDefinition/observation-geneticsGene",

"valueCodeableConcept": {

"coding": [{

"display": "USP1"

}],

"text": "USP1"

}

}, {

"url": "http://hl7.org/fhir/StructureDefinition/observation-geneticsDNASequenceVariantType",

"valueCodeableConcept": {

"coding": [{

"system": "http://vernomen.hgvs.org",

"code": "HGVS.c:c.*3116G>A",

"display": "HGVS.c:c.*3116G>A"

}],

"text": "HGVS.c:c.*3116G>A"

}

}, {

"url": "http://hl7.org/fhir/StructureDefinition/observation-geneticsGene",

"valueCodeableConcept": {

"coding": [{

"display": "DOCK7"

}],

"text": "DOCK7"

}

}, {

"url": "http://hl7.org/fhir/StructureDefinition/observation-geneticsDNASequenceVariantType",

"valueCodeableConcept": {

"coding": [{

"system": "http://vernomen.hgvs.org",

"code": "HGVS.c:c.*6407C>T",

"display": "HGVS.c:c.*6407C>T"

}],

"text": "HGVS.c:c.*6407C>T"

}

}, {

"url": "http://hl7.org/fhir/StructureDefinition/observation-geneticsGene",

"valueCodeableConcept": {

"coding": [{

"display": "DOCK7"

}],

"text": "DOCK7"

}

}, {

"url": "http://hl7.org/fhir/StructureDefinition/observation-geneticsDNASequenceVariantType",

"valueCodeableConcept": {

"coding": [{

"system": "http://vernomen.hgvs.org",

"code": "HGVS.c:c.*6401C>T",

"display": "HGVS.c:c.*6401C>T"

}],

"text": "HGVS.c:c.*6401C>T"

}

}, {

"url": "http://hl7.org/fhir/StructureDefinition/observation-geneticsGene",

"valueCodeableConcept": {

"coding": [{

"display": "DOCK7"

}],

"text": "DOCK7"

}

}, {

"url": "http://hl7.org/fhir/StructureDefinition/observation-geneticsDNASequenceVariantType",

"valueCodeableConcept": {

"coding": [{

"system": "http://vernomen.hgvs.org",

"code": "HGVS.c:c.*6434C>T",

"display": "HGVS.c:c.*6434C>T"

}],

"text": "HGVS.c:c.*6434C>T"

}

}, {

"url": "http://hl7.org/fhir/StructureDefinition/observation-geneticsGene",

"valueCodeableConcept": {

"coding": [{

"display": "USP1-DOCK7"

}],

"text": "USP1-DOCK7"

}

}, {

"url": "http://hl7.org/fhir/StructureDefinition/observation-geneticsGene",

"valueCodeableConcept": {

"coding": [{

"display": "USP1"

}],

"text": "USP1"

}

}, {

"url": "http://hl7.org/fhir/StructureDefinition/observation-geneticsDNASequenceVariantType",

"valueCodeableConcept": {

"coding": [{

"system": "http://vernomen.hgvs.org",

"code": "HGVS.c:c.*2686T>A",

"display": "HGVS.c:c.*2686T>A"

}],

"text": "HGVS.c:c.*2686T>A"

}

}, {

"url": "http://hl7.org/fhir/StructureDefinition/observation-geneticsGene",

"valueCodeableConcept": {

"coding": [{

"display": "DOCK7"

}],

"text": "DOCK7"

}

}, {

"url": "http://hl7.org/fhir/StructureDefinition/observation-geneticsDNASequenceVariantType",

"valueCodeableConcept": {

"coding": [{

"system": "http://vernomen.hgvs.org",

"code": "HGVS.c:c.*6494A>T",

"display": "HGVS.c:c.*6494A>T"

}],

"text": "HGVS.c:c.*6494A>T"

}

}, {

"url": "http://hl7.org/fhir/StructureDefinition/observation-geneticsGene",

"valueCodeableConcept": {

"coding": [{

"display": "USP1"

}],

"text": "USP1"

}

}, {

"url": "http://hl7.org/fhir/StructureDefinition/observation-geneticsDNASequenceVariantType",

"valueCodeableConcept": {

"coding": [{

"system": "http://vernomen.hgvs.org",

"code": "HGVS.c:c.*2591T>A",

"display": "HGVS.c:c.*2591T>A"

}],

"text": "HGVS.c:c.*2591T>A"

}

}, {

"url": "http://hl7.org/fhir/StructureDefinition/observation-geneticsGene",

"valueCodeableConcept": {

"coding": [{

"display": "USP1"

}],

"text": "USP1"

}

}, {

"url": "http://hl7.org/fhir/StructureDefinition/observation-geneticsDNASequenceVariantType",

"valueCodeableConcept": {

"coding": [{

"system": "http://vernomen.hgvs.org",

"code": "HGVS.c:c.*3116T>A",

"display": "HGVS.c:c.*3116T>A"

}],

"text": "HGVS.c:c.*3116T>A"

}

}, {

"url": "http://hl7.org/fhir/StructureDefinition/observation-geneticsGene",

"valueCodeableConcept": {

"coding": [{

"display": "DOCK7"

}],

"text": "DOCK7"

}

}, {

"url": "http://hl7.org/fhir/StructureDefinition/observation-geneticsDNASequenceVariantType",

"valueCodeableConcept": {

"coding": [{

"system": "http://vernomen.hgvs.org",

"code": "HGVS.c:c.*6407A>T",

"display": "HGVS.c:c.*6407A>T"

}],

"text": "HGVS.c:c.*6407A>T"

}

}, {

"url": "http://hl7.org/fhir/StructureDefinition/observation-geneticsGene",

"valueCodeableConcept": {

"coding": [{

"display": "DOCK7"

}],

"text": "DOCK7"

}

}, {

"url": "http://hl7.org/fhir/StructureDefinition/observation-geneticsDNASequenceVariantType",

"valueCodeableConcept": {

"coding": [{

"system": "http://vernomen.hgvs.org",

"code": "HGVS.c:c.*6401A>T",

"display": "HGVS.c:c.*6401A>T"

}],

"text": "HGVS.c:c.*6401A>T"

}

}, {

"url": "http://hl7.org/fhir/StructureDefinition/observation-geneticsGene",

"valueCodeableConcept": {

"coding": [{

"display": "DOCK7"

}],

"text": "DOCK7"

}

}, {

"url": "http://hl7.org/fhir/StructureDefinition/observation-geneticsDNASequenceVariantType",

"valueCodeableConcept": {

"coding": [{

"system": "http://vernomen.hgvs.org",

"code": "HGVS.c:c.*6434A>T",

"display": "HGVS.c:c.*6434A>T"

}],

"text": "HGVS.c:c.*6434A>T"

}

}, {

"url": "http://hl7.org/fhir/StructureDefinition/observation-geneticsGene",

"valueCodeableConcept": {

"coding": [{

"display": "USP1-DOCK7"

}],

"text": "USP1-DOCK7"

}

}, {

"url": "http://hl7.org/fhir/StructureDefinition/observation-geneticsGene",

"valueCodeableConcept": {

"coding": [{

"display": "USP1"

}],

"text": "USP1"

}

}, {

"url": "http://hl7.org/fhir/StructureDefinition/observation-geneticsDNASequenceVariantType",

"valueCodeableConcept": {

"coding": [{

"system": "http://vernomen.hgvs.org",

"code": "HGVS.c:c.*2686delA",

"display": "HGVS.c:c.*2686delA"

}],

"text": "HGVS.c:c.*2686delA"

}

}, {

"url": "http://hl7.org/fhir/StructureDefinition/observation-geneticsGene",

"valueCodeableConcept": {

"coding": [{

"display": "DOCK7"

}],

"text": "DOCK7"

}

}, {

"url": "http://hl7.org/fhir/StructureDefinition/observation-geneticsDNASequenceVariantType",

"valueCodeableConcept": {

"coding": [{

"system": "http://vernomen.hgvs.org",

"code": "HGVS.c:c.*6494delT",

"display": "HGVS.c:c.*6494delT"

}],

"text": "HGVS.c:c.*6494delT"

}

}, {

"url": "http://hl7.org/fhir/StructureDefinition/observation-geneticsGene",

"valueCodeableConcept": {

"coding": [{

"display": "USP1"

}],

"text": "USP1"

}

}, {

"url": "http://hl7.org/fhir/StructureDefinition/observation-geneticsDNASequenceVariantType",

"valueCodeableConcept": {

"coding": [{

"system": "http://vernomen.hgvs.org",

"code": "HGVS.c:c.*2591delA",

"display": "HGVS.c:c.*2591delA"

}],

"text": "HGVS.c:c.*2591delA"

}

}, {

"url": "http://hl7.org/fhir/StructureDefinition/observation-geneticsGene",

"valueCodeableConcept": {

"coding": [{

"display": "USP1"

}],

"text": "USP1"

}

}, {

"url": "http://hl7.org/fhir/StructureDefinition/observation-geneticsDNASequenceVariantType",

"valueCodeableConcept": {

"coding": [{

"system": "http://vernomen.hgvs.org",

"code": "HGVS.c:c.*3116delA",

"display": "HGVS.c:c.*3116delA"

}],

"text": "HGVS.c:c.*3116delA"

}

}, {

"url": "http://hl7.org/fhir/StructureDefinition/observation-geneticsGene",

"valueCodeableConcept": {

"coding": [{

"display": "DOCK7"

}],

"text": "DOCK7"

}

}, {

"url": "http://hl7.org/fhir/StructureDefinition/observation-geneticsDNASequenceVariantType",

"valueCodeableConcept": {

"coding": [{

"system": "http://vernomen.hgvs.org",

"code": "HGVS.c:c.*6407delT",

"display": "HGVS.c:c.*6407delT"

}],

"text": "HGVS.c:c.*6407delT"

}

}, {

"url": "http://hl7.org/fhir/StructureDefinition/observation-geneticsGene",

"valueCodeableConcept": {

"coding": [{

"display": "DOCK7"

}],

"text": "DOCK7"

}

}, {

"url": "http://hl7.org/fhir/StructureDefinition/observation-geneticsDNASequenceVariantType",

"valueCodeableConcept": {

"coding": [{

"system": "http://vernomen.hgvs.org",

"code": "HGVS.c:c.*6401delT",

"display": "HGVS.c:c.*6401delT"

}],

"text": "HGVS.c:c.*6401delT"

}

}, {

"url": "http://hl7.org/fhir/StructureDefinition/observation-geneticsGene",

"valueCodeableConcept": {

"coding": [{

"display": "DOCK7"

}],

"text": "DOCK7"

}

}, {

"url": "http://hl7.org/fhir/StructureDefinition/observation-geneticsDNASequenceVariantType",

"valueCodeableConcept": {

"coding": [{

"system": "http://vernomen.hgvs.org",

"code": "HGVS.c:c.*6434delT",

"display": "HGVS.c:c.*6434delT"

}],

"text": "HGVS.c:c.*6434delT"

}

}, {

"url": "http://hl7.org/fhir/StructureDefinition/observation-geneticsGene",

"valueCodeableConcept": {

"coding": [{

"display": "USP1-DOCK7"

}],

"text": "USP1-DOCK7"

}

}, {

"url": "http://hl7.org/fhir/StructureDefinition/observation-geneticsGene",

"valueCodeableConcept": {

"coding": [{

"display": "USP1"

}],

"text": "USP1"

}

}, {

"url": "http://hl7.org/fhir/StructureDefinition/observation-geneticsDNASequenceVariantType",

"valueCodeableConcept": {

"coding": [{

"system": "http://vernomen.hgvs.org",

"code": "HGVS.c:c.*2686A>G",

"display": "HGVS.c:c.*2686A>G"

}],

"text": "HGVS.c:c.*2686A>G"

}

}, {

"url": "http://hl7.org/fhir/StructureDefinition/observation-geneticsGene",

"valueCodeableConcept": {

"coding": [{

"display": "DOCK7"

}],

"text": "DOCK7"

}

}, {

"url": "http://hl7.org/fhir/StructureDefinition/observation-geneticsDNASequenceVariantType",

"valueCodeableConcept": {

"coding": [{

"system": "http://vernomen.hgvs.org",

"code": "HGVS.c:c.*6494T>C",

"display": "HGVS.c:c.*6494T>C"

}],

"text": "HGVS.c:c.*6494T>C"

}

}, {

"url": "http://hl7.org/fhir/StructureDefinition/observation-geneticsGene",

"valueCodeableConcept": {

"coding": [{

"display": "USP1"

}],

"text": "USP1"

}

}, {

"url": "http://hl7.org/fhir/StructureDefinition/observation-geneticsDNASequenceVariantType",

"valueCodeableConcept": {

"coding": [{

"system": "http://vernomen.hgvs.org",

"code": "HGVS.c:c.*2591A>G",

"display": "HGVS.c:c.*2591A>G"

}],

"text": "HGVS.c:c.*2591A>G"

}

}, {

"url": "http://hl7.org/fhir/StructureDefinition/observation-geneticsGene",

"valueCodeableConcept": {

"coding": [{

"display": "USP1"

}],

"text": "USP1"

}

}, {

"url": "http://hl7.org/fhir/StructureDefinition/observation-geneticsDNASequenceVariantType",

"valueCodeableConcept": {

"coding": [{

"system": "http://vernomen.hgvs.org",

"code": "HGVS.c:c.*3116A>G",

"display": "HGVS.c:c.*3116A>G"

}],

"text": "HGVS.c:c.*3116A>G"

}

}, {

"url": "http://hl7.org/fhir/StructureDefinition/observation-geneticsGene",

"valueCodeableConcept": {

"coding": [{

"display": "DOCK7"

}],

"text": "DOCK7"

}

}, {

"url": "http://hl7.org/fhir/StructureDefinition/observation-geneticsDNASequenceVariantType",

"valueCodeableConcept": {

"coding": [{

"system": "http://vernomen.hgvs.org",

"code": "HGVS.c:c.*6407T>C",

"display": "HGVS.c:c.*6407T>C"

}],

"text": "HGVS.c:c.*6407T>C"

}

}, {

"url": "http://hl7.org/fhir/StructureDefinition/observation-geneticsGene",

"valueCodeableConcept": {

"coding": [{

"display": "DOCK7"

}],

"text": "DOCK7"

}

}, {

"url": "http://hl7.org/fhir/StructureDefinition/observation-geneticsDNASequenceVariantType",

"valueCodeableConcept": {

"coding": [{

"system": "http://vernomen.hgvs.org",

"code": "HGVS.c:c.*6401T>C",

"display": "HGVS.c:c.*6401T>C"

}],

"text": "HGVS.c:c.*6401T>C"

}

}, {

"url": "http://hl7.org/fhir/StructureDefinition/observation-geneticsGene",

"valueCodeableConcept": {

"coding": [{

"display": "DOCK7"

}],

"text": "DOCK7"

}

}, {

"url": "http://hl7.org/fhir/StructureDefinition/observation-geneticsDNASequenceVariantType",

"valueCodeableConcept": {

"coding": [{

"system": "http://vernomen.hgvs.org",

"code": "HGVS.c:c.*6434T>C",

"display": "HGVS.c:c.*6434T>C"

}],

"text": "HGVS.c:c.*6434T>C"

}

}, {

"url": "http://hl7.org/fhir/StructureDefinition/observation-geneticsGene",

"valueCodeableConcept": {

"coding": [{

"display": "USP1-DOCK7"

}],

"text": "USP1-DOCK7"

}

}, {

"url": "http://hl7.org/fhir/StructureDefinition/observation-geneticsGene",

"valueCodeableConcept": {

"coding": [{

"display": "USP1"

}],

"text": "USP1"

}

}, {

"url": "http://hl7.org/fhir/StructureDefinition/observation-geneticsDNASequenceVariantType",

"valueCodeableConcept": {

"coding": [{

"system": "http://vernomen.hgvs.org",

"code": "HGVS.c:c.*2686delT",

"display": "HGVS.c:c.*2686delT"

}],

"text": "HGVS.c:c.*2686delT"

}

}, {

"url": "http://hl7.org/fhir/StructureDefinition/observation-geneticsGene",

"valueCodeableConcept": {

"coding": [{

"display": "DOCK7"

}],

"text": "DOCK7"

}

}, {

"url": "http://hl7.org/fhir/StructureDefinition/observation-geneticsDNASequenceVariantType",

"valueCodeableConcept": {

"coding": [{

"system": "http://vernomen.hgvs.org",

"code": "HGVS.c:c.*6494delA",

"display": "HGVS.c:c.*6494delA"

}],

"text": "HGVS.c:c.*6494delA"

}

}, {

"url": "http://hl7.org/fhir/StructureDefinition/observation-geneticsGene",

"valueCodeableConcept": {

"coding": [{

"display": "USP1"

}],

"text": "USP1"

}

}, {

"url": "http://hl7.org/fhir/StructureDefinition/observation-geneticsDNASequenceVariantType",

"valueCodeableConcept": {

"coding": [{

"system": "http://vernomen.hgvs.org",

"code": "HGVS.c:c.*2591delT",

"display": "HGVS.c:c.*2591delT"

}],

"text": "HGVS.c:c.*2591delT"

}

}, {

"url": "http://hl7.org/fhir/StructureDefinition/observation-geneticsGene",

"valueCodeableConcept": {

"coding": [{

"display": "USP1"

}],

"text": "USP1"

}

}, {

"url": "http://hl7.org/fhir/StructureDefinition/observation-geneticsDNASequenceVariantType",

"valueCodeableConcept": {

"coding": [{

"system": "http://vernomen.hgvs.org",

"code": "HGVS.c:c.*3116delT",

"display": "HGVS.c:c.*3116delT"

}],

"text": "HGVS.c:c.*3116delT"

}

}, {

"url": "http://hl7.org/fhir/StructureDefinition/observation-geneticsGene",

"valueCodeableConcept": {

"coding": [{

"display": "DOCK7"

}],

"text": "DOCK7"

}

}, {

"url": "http://hl7.org/fhir/StructureDefinition/observation-geneticsDNASequenceVariantType",

"valueCodeableConcept": {

"coding": [{

"system": "http://vernomen.hgvs.org",

"code": "HGVS.c:c.*6407delA",

"display": "HGVS.c:c.*6407delA"

}],

"text": "HGVS.c:c.*6407delA"

}

}, {

"url": "http://hl7.org/fhir/StructureDefinition/observation-geneticsGene",

"valueCodeableConcept": {

"coding": [{

"display": "DOCK7"

}],

"text": "DOCK7"

}

}, {

"url": "http://hl7.org/fhir/StructureDefinition/observation-geneticsDNASequenceVariantType",

"valueCodeableConcept": {

"coding": [{

"system": "http://vernomen.hgvs.org",

"code": "HGVS.c:c.*6401delA",

"display": "HGVS.c:c.*6401delA"

}],

"text": "HGVS.c:c.*6401delA"

}

}, {

"url": "http://hl7.org/fhir/StructureDefinition/observation-geneticsGene",

"valueCodeableConcept": {

"coding": [{

"display": "DOCK7"

}],

"text": "DOCK7"

}

}, {

"url": "http://hl7.org/fhir/StructureDefinition/observation-geneticsDNASequenceVariantType",

"valueCodeableConcept": {

"coding": [{

"system": "http://vernomen.hgvs.org",

"code": "HGVS.c:c.*6434delA",

"display": "HGVS.c:c.*6434delA"

}],

"text": "HGVS.c:c.*6434delA"

}

}, {

"url": "http://hl7.org/fhir/StructureDefinition/observation-geneticsGene",

"valueCodeableConcept": {

"coding": [{

"display": "USP1-DOCK7"

}],

"text": "USP1-DOCK7"

}

}, {

"url": "http://hl7.org/fhir/StructureDefinition/observation-geneticsGene",

"valueCodeableConcept": {

"coding": [{

"display": "USP1"

}],

"text": "USP1"

}

}, {

"url": "http://hl7.org/fhir/StructureDefinition/observation-geneticsDNASequenceVariantType",

"valueCodeableConcept": {

"coding": [{

"system": "http://vernomen.hgvs.org",

"code": "HGVS.c:c.*2686G>C",

"display": "HGVS.c:c.*2686G>C"

}],

"text": "HGVS.c:c.*2686G>C"

}

}, {

"url": "http://hl7.org/fhir/StructureDefinition/observation-geneticsGene",

"valueCodeableConcept": {

"coding": [{

"display": "DOCK7"

}],

"text": "DOCK7"

}

}, {

"url": "http://hl7.org/fhir/StructureDefinition/observation-geneticsDNASequenceVariantType",

"valueCodeableConcept": {

"coding": [{

"system": "http://vernomen.hgvs.org",

"code": "HGVS.c:c.*6494C>G",

"display": "HGVS.c:c.*6494C>G"

}],

"text": "HGVS.c:c.*6494C>G"

}

}, {

"url": "http://hl7.org/fhir/StructureDefinition/observation-geneticsGene",

"valueCodeableConcept": {

"coding": [{

"display": "USP1"

}],

"text": "USP1"

}

}, {

"url": "http://hl7.org/fhir/StructureDefinition/observation-geneticsDNASequenceVariantType",

"valueCodeableConcept": {

"coding": [{

"system": "http://vernomen.hgvs.org",

"code": "HGVS.c:c.*2591G>C",

"display": "HGVS.c:c.*2591G>C"

}],

"text": "HGVS.c:c.*2591G>C"

}

}, {

"url": "http://hl7.org/fhir/StructureDefinition/observation-geneticsGene",

"valueCodeableConcept": {

"coding": [{

"display": "USP1"

}],

"text": "USP1"

}

}, {

"url": "http://hl7.org/fhir/StructureDefinition/observation-geneticsDNASequenceVariantType",

"valueCodeableConcept": {

"coding": [{

"system": "http://vernomen.hgvs.org",

"code": "HGVS.c:c.*3116G>C",

"display": "HGVS.c:c.*3116G>C"

}],

"text": "HGVS.c:c.*3116G>C"

}

}, {

"url": "http://hl7.org/fhir/StructureDefinition/observation-geneticsGene",

"valueCodeableConcept": {

"coding": [{

"display": "DOCK7"

}],

"text": "DOCK7"

}

}, {

"url": "http://hl7.org/fhir/StructureDefinition/observation-geneticsDNASequenceVariantType",

"valueCodeableConcept": {

"coding": [{

"system": "http://vernomen.hgvs.org",

"code": "HGVS.c:c.*6407C>G",

"display": "HGVS.c:c.*6407C>G"

}],

"text": "HGVS.c:c.*6407C>G"

}

}, {

"url": "http://hl7.org/fhir/StructureDefinition/observation-geneticsGene",

"valueCodeableConcept": {

"coding": [{

"display": "DOCK7"

}],

"text": "DOCK7"

}

}, {

"url": "http://hl7.org/fhir/StructureDefinition/observation-geneticsDNASequenceVariantType",

"valueCodeableConcept": {

"coding": [{

"system": "http://vernomen.hgvs.org",

"code": "HGVS.c:c.*6401C>G",

"display": "HGVS.c:c.*6401C>G"

}],

"text": "HGVS.c:c.*6401C>G"

}

}, {

"url": "http://hl7.org/fhir/StructureDefinition/observation-geneticsGene",

"valueCodeableConcept": {

"coding": [{

"display": "DOCK7"

}],

"text": "DOCK7"

}

}, {

"url": "http://hl7.org/fhir/StructureDefinition/observation-geneticsDNASequenceVariantType",

"valueCodeableConcept": {

"coding": [{

"system": "http://vernomen.hgvs.org",

"code": "HGVS.c:c.*6434C>G",

"display": "HGVS.c:c.*6434C>G"

}],

"text": "HGVS.c:c.*6434C>G"

}

}, {

"url": "http://hl7.org/fhir/StructureDefinition/observation-geneticsGene",

"valueCodeableConcept": {

"coding": [{

"display": "USP1-DOCK7"

}],

"text": "USP1-DOCK7"

}

}, {

"url": "http://hl7.org/fhir/StructureDefinition/observation-geneticsGene",

"valueCodeableConcept": {

"coding": [{

"display": "USP1"

}],

"text": "USP1"

}

}, {

"url": "http://hl7.org/fhir/StructureDefinition/observation-geneticsDNASequenceVariantType",

"valueCodeableConcept": {

"coding": [{

"system": "http://vernomen.hgvs.org",

"code": "HGVS.c:c.*2686_*2686insAT",

"display": "HGVS.c:c.*2686_*2686insAT"

}],

"text": "HGVS.c:c.*2686_*2686insAT"

}

}, {

"url": "http://hl7.org/fhir/StructureDefinition/observation-geneticsGene",

"valueCodeableConcept": {

"coding": [{

"display": "DOCK7"

}],

"text": "DOCK7"

}

}, {

"url": "http://hl7.org/fhir/StructureDefinition/observation-geneticsDNASequenceVariantType",

"valueCodeableConcept": {

"coding": [{

"system": "http://vernomen.hgvs.org",

"code": "HGVS.c:c.*6494_*6494insAT",

"display": "HGVS.c:c.*6494_*6494insAT"

}],

"text": "HGVS.c:c.*6494_*6494insAT"

}

}, {

"url": "http://hl7.org/fhir/StructureDefinition/observation-geneticsGene",

"valueCodeableConcept": {

"coding": [{

"display": "USP1"

}],

"text": "USP1"

}

}, {

"url": "http://hl7.org/fhir/StructureDefinition/observation-geneticsDNASequenceVariantType",

"valueCodeableConcept": {

"coding": [{

"system": "http://vernomen.hgvs.org",

"code": "HGVS.c:c.*2591_*2591insAT",

"display": "HGVS.c:c.*2591_*2591insAT"

}],

"text": "HGVS.c:c.*2591_*2591insAT"

}

}, {

"url": "http://hl7.org/fhir/StructureDefinition/observation-geneticsGene",

"valueCodeableConcept": {

"coding": [{

"display": "USP1"

}],

"text": "USP1"

}

}, {

"url": "http://hl7.org/fhir/StructureDefinition/observation-geneticsDNASequenceVariantType",

"valueCodeableConcept": {

"coding": [{

"system": "http://vernomen.hgvs.org",

"code": "HGVS.c:c.*3116_*3116insAT",

"display": "HGVS.c:c.*3116_*3116insAT"

}],

"text": "HGVS.c:c.*3116_*3116insAT"

}

}, {

"url": "http://hl7.org/fhir/StructureDefinition/observation-geneticsGene",

"valueCodeableConcept": {

"coding": [{

"display": "DOCK7"

}],

"text": "DOCK7"

}

}, {

"url": "http://hl7.org/fhir/StructureDefinition/observation-geneticsDNASequenceVariantType",

"valueCodeableConcept": {

"coding": [{

"system": "http://vernomen.hgvs.org",

"code": "HGVS.c:c.*6407_*6407insAT",

"display": "HGVS.c:c.*6407_*6407insAT"

}],

"text": "HGVS.c:c.*6407_*6407insAT"

}

}, {

"url": "http://hl7.org/fhir/StructureDefinition/observation-geneticsGene",

"valueCodeableConcept": {

"coding": [{

"display": "DOCK7"

}],

"text": "DOCK7"

}

}, {

"url": "http://hl7.org/fhir/StructureDefinition/observation-geneticsDNASequenceVariantType",

"valueCodeableConcept": {

"coding": [{

"system": "http://vernomen.hgvs.org",

"code": "HGVS.c:c.*6401_*6401insAT",

"display": "HGVS.c:c.*6401_*6401insAT"

}],

"text": "HGVS.c:c.*6401_*6401insAT"

}

}, {

"url": "http://hl7.org/fhir/StructureDefinition/observation-geneticsGene",

"valueCodeableConcept": {

"coding": [{

"display": "DOCK7"

}],

"text": "DOCK7"

}

}, {

"url": "http://hl7.org/fhir/StructureDefinition/observation-geneticsDNASequenceVariantType",

"valueCodeableConcept": {

"coding": [{

"system": "http://vernomen.hgvs.org",

"code": "HGVS.c:c.*6434_*6434insAT",

"display": "HGVS.c:c.*6434_*6434insAT"

}],

"text": "HGVS.c:c.*6434_*6434insAT"

}

}, {

"url": "http://hl7.org/fhir/StructureDefinition/observation-geneticsGene",

"valueCodeableConcept": {

"coding": [{

"display": "USP1-DOCK7"

}],

"text": "USP1-DOCK7"

}

}, {

"url": "http://hl7.org/fhir/StructureDefinition/observation-geneticsDNASequenceVariantType",

"valueCodeableConcept": {

"coding": [{

"system": "http://vernomen.hgvs.org",

"code": "HGVS.c:n.null_nullinsAT}]",

"display": "HGVS.c:n.null_nullinsAT}]"

}],

"text": "HGVS.c:n.null_nullinsAT}]"

}

}, {

"url": "http://hl7.org/fhir/StructureDefinition/observation-geneticsGene",

"valueCodeableConcept": {

"coding": [{

"display": "USP1"

}],

"text": "USP1"

}

}, {

"url": "http://hl7.org/fhir/StructureDefinition/observation-geneticsDNASequenceVariantType",

"valueCodeableConcept": {

"coding": [{

"system": "http://vernomen.hgvs.org",

"code": "HGVS.c:c.*2686A>G",

"display": "HGVS.c:c.*2686A>G"

}],

"text": "HGVS.c:c.*2686A>G"

}

}, {

"url": "http://hl7.org/fhir/StructureDefinition/observation-geneticsGene",

"valueCodeableConcept": {

"coding": [{

"display": "DOCK7"

}],

"text": "DOCK7"

}

}, {

"url": "http://hl7.org/fhir/StructureDefinition/observation-geneticsDNASequenceVariantType",

"valueCodeableConcept": {

"coding": [{

"system": "http://vernomen.hgvs.org",

"code": "HGVS.c:c.*6494T>C",

"display": "HGVS.c:c.*6494T>C"

}],

"text": "HGVS.c:c.*6494T>C"

}

}, {

"url": "http://hl7.org/fhir/StructureDefinition/observation-geneticsGene",

"valueCodeableConcept": {

"coding": [{

"display": "USP1"

}],

"text": "USP1"

}

}, {

"url": "http://hl7.org/fhir/StructureDefinition/observation-geneticsDNASequenceVariantType",

"valueCodeableConcept": {

"coding": [{

"system": "http://vernomen.hgvs.org",

"code": "HGVS.c:c.*2591A>G",

"display": "HGVS.c:c.*2591A>G"

}],

"text": "HGVS.c:c.*2591A>G"

}

}, {

"url": "http://hl7.org/fhir/StructureDefinition/observation-geneticsGene",

"valueCodeableConcept": {

"coding": [{

"display": "USP1"

}],

"text": "USP1"

}

}, {

"url": "http://hl7.org/fhir/StructureDefinition/observation-geneticsDNASequenceVariantType",

"valueCodeableConcept": {

"coding": [{

"system": "http://vernomen.hgvs.org",

"code": "HGVS.c:c.*3116A>G",

"display": "HGVS.c:c.*3116A>G"

}],

"text": "HGVS.c:c.*3116A>G"

}

}, {

"url": "http://hl7.org/fhir/StructureDefinition/observation-geneticsGene",

"valueCodeableConcept": {

"coding": [{

"display": "DOCK7"

}],

"text": "DOCK7"

}

}, {

"url": "http://hl7.org/fhir/StructureDefinition/observation-geneticsDNASequenceVariantType",

"valueCodeableConcept": {

"coding": [{

"system": "http://vernomen.hgvs.org",

"code": "HGVS.c:c.*6407T>C",

"display": "HGVS.c:c.*6407T>C"

}],

"text": "HGVS.c:c.*6407T>C"

}

}, {

"url": "http://hl7.org/fhir/StructureDefinition/observation-geneticsGene",

"valueCodeableConcept": {

"coding": [{

"display": "DOCK7"

}],

"text": "DOCK7"

}

}, {

"url": "http://hl7.org/fhir/StructureDefinition/observation-geneticsDNASequenceVariantType",

"valueCodeableConcept": {

"coding": [{

"system": "http://vernomen.hgvs.org",

"code": "HGVS.c:c.*6401T>C",

"display": "HGVS.c:c.*6401T>C"

}],

"text": "HGVS.c:c.*6401T>C"

}

}, {

"url": "http://hl7.org/fhir/StructureDefinition/observation-geneticsGene",

"valueCodeableConcept": {

"coding": [{

"display": "DOCK7"

}],

"text": "DOCK7"

}

}, {

"url": "http://hl7.org/fhir/StructureDefinition/observation-geneticsDNASequenceVariantType",

"valueCodeableConcept": {

"coding": [{

"system": "http://vernomen.hgvs.org",

"code": "HGVS.c:c.*6434T>C",

"display": "HGVS.c:c.*6434T>C"

}],

"text": "HGVS.c:c.*6434T>C"

}

}, {

"url": "http://hl7.org/fhir/StructureDefinition/observation-geneticsGene",

"valueCodeableConcept": {

"coding": [{

"display": "USP1-DOCK7"

}],

"text": "USP1-DOCK7"

}

}, {

"url": "http://hl7.org/fhir/StructureDefinition/observation-geneticsGene",

"valueCodeableConcept": {

"coding": [{

"display": "USP1"

}],

"text": "USP1"

}

}, {

"url": "http://hl7.org/fhir/StructureDefinition/observation-geneticsDNASequenceVariantType",

"valueCodeableConcept": {

"coding": [{

"system": "http://vernomen.hgvs.org",

"code": "HGVS.c:c.*2686A>G",

"display": "HGVS.c:c.*2686A>G"

}],

"text": "HGVS.c:c.*2686A>G"

}

}, {

"url": "http://hl7.org/fhir/StructureDefinition/observation-geneticsGene",

"valueCodeableConcept": {

"coding": [{

"display": "DOCK7"

}],

"text": "DOCK7"

}

}, {

"url": "http://hl7.org/fhir/StructureDefinition/observation-geneticsDNASequenceVariantType",

"valueCodeableConcept": {

"coding": [{

"system": "http://vernomen.hgvs.org",

"code": "HGVS.c:c.*6494T>C",

"display": "HGVS.c:c.*6494T>C"

}],

"text": "HGVS.c:c.*6494T>C"

}

}, {

"url": "http://hl7.org/fhir/StructureDefinition/observation-geneticsGene",

"valueCodeableConcept": {

"coding": [{

"display": "USP1"

}],

"text": "USP1"

}

}, {

"url": "http://hl7.org/fhir/StructureDefinition/observation-geneticsDNASequenceVariantType",

"valueCodeableConcept": {

"coding": [{

"system": "http://vernomen.hgvs.org",

"code": "HGVS.c:c.*2591A>G",

"display": "HGVS.c:c.*2591A>G"

}],

"text": "HGVS.c:c.*2591A>G"

}

}, {

"url": "http://hl7.org/fhir/StructureDefinition/observation-geneticsGene",

"valueCodeableConcept": {

"coding": [{

"display": "USP1"

}],

"text": "USP1"

}

}, {

"url": "http://hl7.org/fhir/StructureDefinition/observation-geneticsDNASequenceVariantType",

"valueCodeableConcept": {

"coding": [{

"system": "http://vernomen.hgvs.org",

"code": "HGVS.c:c.*3116A>G",

"display": "HGVS.c:c.*3116A>G"

}],

"text": "HGVS.c:c.*3116A>G"

}

}, {

"url": "http://hl7.org/fhir/StructureDefinition/observation-geneticsGene",

"valueCodeableConcept": {

"coding": [{

"display": "DOCK7"

}],

"text": "DOCK7"

}

}, {

"url": "http://hl7.org/fhir/StructureDefinition/observation-geneticsDNASequenceVariantType",

"valueCodeableConcept": {

"coding": [{

"system": "http://vernomen.hgvs.org",

"code": "HGVS.c:c.*6407T>C",

"display": "HGVS.c:c.*6407T>C"

}],

"text": "HGVS.c:c.*6407T>C"

}

}, {

"url": "http://hl7.org/fhir/StructureDefinition/observation-geneticsGene",

"valueCodeableConcept": {

"coding": [{

"display": "DOCK7"

}],

"text": "DOCK7"

}

}, {

"url": "http://hl7.org/fhir/StructureDefinition/observation-geneticsDNASequenceVariantType",

"valueCodeableConcept": {

"coding": [{

"system": "http://vernomen.hgvs.org",

"code": "HGVS.c:c.*6401T>C",

"display": "HGVS.c:c.*6401T>C"

}],

"text": "HGVS.c:c.*6401T>C"

}

}, {

"url": "http://hl7.org/fhir/StructureDefinition/observation-geneticsGene",

"valueCodeableConcept": {

"coding": [{

"display": "DOCK7"

}],

"text": "DOCK7"

}

}, {

"url": "http://hl7.org/fhir/StructureDefinition/observation-geneticsDNASequenceVariantType",

"valueCodeableConcept": {

"coding": [{

"system": "http://vernomen.hgvs.org",

"code": "HGVS.c:c.*6434T>C",

"display": "HGVS.c:c.*6434T>C"

}],

"text": "HGVS.c:c.*6434T>C"

}

}, {

"url": "http://hl7.org/fhir/StructureDefinition/observation-geneticsGene",

"valueCodeableConcept": {

"coding": [{

"display": "USP1-DOCK7"

}],

"text": "USP1-DOCK7"

}

}, {

"url": "http://hl7.org/fhir/StructureDefinition/observation-geneticsGene",

"valueCodeableConcept": {

"coding": [{

"display": "USP1"

}],

"text": "USP1"

}

}, {

"url": "http://hl7.org/fhir/StructureDefinition/observation-geneticsDNASequenceVariantType",

"valueCodeableConcept": {

"coding": [{

"system": "http://vernomen.hgvs.org",

"code": "HGVS.c:c.*2686C>A",

"display": "HGVS.c:c.*2686C>A"

}],

"text": "HGVS.c:c.*2686C>A"

}

}, {

"url": "http://hl7.org/fhir/StructureDefinition/observation-geneticsGene",

"valueCodeableConcept": {

"coding": [{

"display": "DOCK7"

}],

"text": "DOCK7"

}

}, {

"url": "http://hl7.org/fhir/StructureDefinition/observation-geneticsDNASequenceVariantType",

"valueCodeableConcept": {

"coding": [{

"system": "http://vernomen.hgvs.org",

"code": "HGVS.c:c.*6494G>T",

"display": "HGVS.c:c.*6494G>T"

}],

"text": "HGVS.c:c.*6494G>T"

}

}, {

"url": "http://hl7.org/fhir/StructureDefinition/observation-geneticsGene",

"valueCodeableConcept": {

"coding": [{

"display": "USP1"

}],

"text": "USP1"

}

}, {

"url": "http://hl7.org/fhir/StructureDefinition/observation-geneticsDNASequenceVariantType",

"valueCodeableConcept": {

"coding": [{

"system": "http://vernomen.hgvs.org",

"code": "HGVS.c:c.*2591C>A",

"display": "HGVS.c:c.*2591C>A"

}],

"text": "HGVS.c:c.*2591C>A"

}

}, {

"url": "http://hl7.org/fhir/StructureDefinition/observation-geneticsGene",

"valueCodeableConcept": {

"coding": [{

"display": "USP1"

}],

"text": "USP1"

}

}, {

"url": "http://hl7.org/fhir/StructureDefinition/observation-geneticsDNASequenceVariantType",

"valueCodeableConcept": {

"coding": [{

"system": "http://vernomen.hgvs.org",

"code": "HGVS.c:c.*3116C>A",

"display": "HGVS.c:c.*3116C>A"

}],

"text": "HGVS.c:c.*3116C>A"

}

}, {

"url": "http://hl7.org/fhir/StructureDefinition/observation-geneticsGene",

"valueCodeableConcept": {

"coding": [{

"display": "DOCK7"

}],

"text": "DOCK7"

}

}, {

"url": "http://hl7.org/fhir/StructureDefinition/observation-geneticsDNASequenceVariantType",

"valueCodeableConcept": {

"coding": [{

"system": "http://vernomen.hgvs.org",

"code": "HGVS.c:c.*6407G>T",

"display": "HGVS.c:c.*6407G>T"

}],

"text": "HGVS.c:c.*6407G>T"

}

}, {

"url": "http://hl7.org/fhir/StructureDefinition/observation-geneticsGene",

"valueCodeableConcept": {

"coding": [{

"display": "DOCK7"

}],

"text": "DOCK7"

}

}, {

"url": "http://hl7.org/fhir/StructureDefinition/observation-geneticsDNASequenceVariantType",

"valueCodeableConcept": {

"coding": [{

"system": "http://vernomen.hgvs.org",

"code": "HGVS.c:c.*6401G>T",

"display": "HGVS.c:c.*6401G>T"

}],

"text": "HGVS.c:c.*6401G>T"

}

}, {

"url": "http://hl7.org/fhir/StructureDefinition/observation-geneticsGene",

"valueCodeableConcept": {

"coding": [{

"display": "DOCK7"

}],

"text": "DOCK7"

}

}, {

"url": "http://hl7.org/fhir/StructureDefinition/observation-geneticsDNASequenceVariantType",

"valueCodeableConcept": {

"coding": [{

"system": "http://vernomen.hgvs.org",

"code": "HGVS.c:c.*6434G>T",

"display": "HGVS.c:c.*6434G>T"

}],

"text": "HGVS.c:c.*6434G>T"

}

}, {

"url": "http://hl7.org/fhir/StructureDefinition/observation-geneticsGene",

"valueCodeableConcept": {

"coding": [{

"display": "USP1-DOCK7"

}],

"text": "USP1-DOCK7"

}

}, {

"url": "http://hl7.org/fhir/StructureDefinition/observation-geneticsGene",

"valueCodeableConcept": {

"coding": [{

"display": "USP1"

}],

"text": "USP1"

}

}, {

"url": "http://hl7.org/fhir/StructureDefinition/observation-geneticsDNASequenceVariantType",

"valueCodeableConcept": {

"coding": [{

"system": "http://vernomen.hgvs.org",

"code": "HGVS.c:c.*2686T>C",

"display": "HGVS.c:c.*2686T>C"

}],

"text": "HGVS.c:c.*2686T>C"

}

}, {

"url": "http://hl7.org/fhir/StructureDefinition/observation-geneticsGene",

"valueCodeableConcept": {

"coding": [{

"display": "USP1"

}],

"text": "USP1"

}

}, {

"url": "http://hl7.org/fhir/StructureDefinition/observation-geneticsDNASequenceVariantType",

"valueCodeableConcept": {

"coding": [{

"system": "http://vernomen.hgvs.org",

"code": "HGVS.c:c.*2591T>C",

"display": "HGVS.c:c.*2591T>C"

}],

"text": "HGVS.c:c.*2591T>C"

}

}, {

"url": "http://hl7.org/fhir/StructureDefinition/observation-geneticsGene",

"valueCodeableConcept": {

"coding": [{

"display": "USP1"

}],

"text": "USP1"

}

}, {

"url": "http://hl7.org/fhir/StructureDefinition/observation-geneticsDNASequenceVariantType",

"valueCodeableConcept": {

"coding": [{

"system": "http://vernomen.hgvs.org",

"code": "HGVS.c:c.*3116T>C",

"display": "HGVS.c:c.*3116T>C"

}],

"text": "HGVS.c:c.*3116T>C"

}

}, {

"url": "http://hl7.org/fhir/StructureDefinition/observation-geneticsGene",

"valueCodeableConcept": {

"coding": [{

"display": "DOCK7"

}],

"text": "DOCK7"

}

}, {

"url": "http://hl7.org/fhir/StructureDefinition/observation-geneticsDNASequenceVariantType",

"valueCodeableConcept": {

"coding": [{

"system": "http://vernomen.hgvs.org",

"code": "HGVS.c:c.6348-295A>G",

"display": "HGVS.c:c.6348-295A>G"

}],

"text": "HGVS.c:c.6348-295A>G"

}

}, {

"url": "http://hl7.org/fhir/StructureDefinition/observation-geneticsGene",

"valueCodeableConcept": {

"coding": [{

"display": "DOCK7"

}],

"text": "DOCK7"

}

}, {

"url": "http://hl7.org/fhir/StructureDefinition/observation-geneticsDNASequenceVariantType",

"valueCodeableConcept": {

"coding": [{

"system": "http://vernomen.hgvs.org",

"code": "HGVS.c:c.6261-295A>G",

"display": "HGVS.c:c.6261-295A>G"

}],

"text": "HGVS.c:c.6261-295A>G"

}

}, {

"url": "http://hl7.org/fhir/StructureDefinition/observation-geneticsGene",

"valueCodeableConcept": {

"coding": [{

"display": "DOCK7"

}],

"text": "DOCK7"

}

}, {

"url": "http://hl7.org/fhir/StructureDefinition/observation-geneticsDNASequenceVariantType",

"valueCodeableConcept": {

"coding": [{

"system": "http://vernomen.hgvs.org",

"code": "HGVS.c:c.6255-295A>G",

"display": "HGVS.c:c.6255-295A>G"

}],

"text": "HGVS.c:c.6255-295A>G"

}

}, {

"url": "http://hl7.org/fhir/StructureDefinition/observation-geneticsGene",

"valueCodeableConcept": {

"coding": [{

"display": "DOCK7"

}],

"text": "DOCK7"

}

}, {

"url": "http://hl7.org/fhir/StructureDefinition/observation-geneticsDNASequenceVariantType",

"valueCodeableConcept": {

"coding": [{

"system": "http://vernomen.hgvs.org",

"code": "HGVS.c:c.6288-295A>G",

"display": "HGVS.c:c.6288-295A>G"

}],

"text": "HGVS.c:c.6288-295A>G"

}

}, {

"url": "http://hl7.org/fhir/StructureDefinition/observation-geneticsGene",

"valueCodeableConcept": {

"coding": [{

"display": "USP1"

}],

"text": "USP1"

}

}, {

"url": "http://hl7.org/fhir/StructureDefinition/observation-geneticsDNASequenceVariantType",

"valueCodeableConcept": {

"coding": [{

"system": "http://vernomen.hgvs.org",

"code": "HGVS.c:c.*2686T>C",

"display": "HGVS.c:c.*2686T>C"

}],

"text": "HGVS.c:c.*2686T>C"

}

}, {

"url": "http://hl7.org/fhir/StructureDefinition/observation-geneticsGene",

"valueCodeableConcept": {

"coding": [{

"display": "USP1"

}],

"text": "USP1"

}

}, {

"url": "http://hl7.org/fhir/StructureDefinition/observation-geneticsDNASequenceVariantType",

"valueCodeableConcept": {

"coding": [{

"system": "http://vernomen.hgvs.org",

"code": "HGVS.c:c.*2591T>C",

"display": "HGVS.c:c.*2591T>C"

}],

"text": "HGVS.c:c.*2591T>C"

}

}, {

"url": "http://hl7.org/fhir/StructureDefinition/observation-geneticsGene",

"valueCodeableConcept": {

"coding": [{

"display": "USP1"

}],

"text": "USP1"

}

}, {

"url": "http://hl7.org/fhir/StructureDefinition/observation-geneticsDNASequenceVariantType",

"valueCodeableConcept": {

"coding": [{

"system": "http://vernomen.hgvs.org",

"code": "HGVS.c:c.*3116T>C",

"display": "HGVS.c:c.*3116T>C"

}],

"text": "HGVS.c:c.*3116T>C"

}

}, {

"url": "http://hl7.org/fhir/StructureDefinition/observation-geneticsGene",

"valueCodeableConcept": {

"coding": [{

"display": "DOCK7"

}],

"text": "DOCK7"

}

}, {

"url": "http://hl7.org/fhir/StructureDefinition/observation-geneticsDNASequenceVariantType",

"valueCodeableConcept": {

"coding": [{

"system": "http://vernomen.hgvs.org",

"code": "HGVS.c:c.6348-459A>G",

"display": "HGVS.c:c.6348-459A>G"

}],

"text": "HGVS.c:c.6348-459A>G"

}

}, {

"url": "http://hl7.org/fhir/StructureDefinition/observation-geneticsGene",

"valueCodeableConcept": {

"coding": [{

"display": "DOCK7"

}],

"text": "DOCK7"

}

}, {

"url": "http://hl7.org/fhir/StructureDefinition/observation-geneticsDNASequenceVariantType",

"valueCodeableConcept": {

"coding": [{

"system": "http://vernomen.hgvs.org",

"code": "HGVS.c:c.6261-459A>G",

"display": "HGVS.c:c.6261-459A>G"

}],

"text": "HGVS.c:c.6261-459A>G"

}

}, {

"url": "http://hl7.org/fhir/StructureDefinition/observation-geneticsGene",

"valueCodeableConcept": {

"coding": [{

"display": "DOCK7"

}],

"text": "DOCK7"

}

}, {

"url": "http://hl7.org/fhir/StructureDefinition/observation-geneticsDNASequenceVariantType",

"valueCodeableConcept": {

"coding": [{

"system": "http://vernomen.hgvs.org",

"code": "HGVS.c:c.6255-459A>G",

"display": "HGVS.c:c.6255-459A>G"

}],

"text": "HGVS.c:c.6255-459A>G"

}

}, {

"url": "http://hl7.org/fhir/StructureDefinition/observation-geneticsGene",

"valueCodeableConcept": {

"coding": [{

"display": "DOCK7"

}],

"text": "DOCK7"

}

}, {

"url": "http://hl7.org/fhir/StructureDefinition/observation-geneticsDNASequenceVariantType",

"valueCodeableConcept": {

"coding": [{

"system": "http://vernomen.hgvs.org",

"code": "HGVS.c:c.6288-459A>G",

"display": "HGVS.c:c.6288-459A>G"

}],

"text": "HGVS.c:c.6288-459A>G"

}

}, {

"url": "http://hl7.org/fhir/StructureDefinition/observation-geneticsGene",

"valueCodeableConcept": {

"coding": [{

"display": "LIPG"

}],

"text": "LIPG"

}

}, {

"url": "http://hl7.org/fhir/StructureDefinition/observation-geneticsDNASequenceVariantType",

"valueCodeableConcept": {

"coding": [{

"system": "http://vernomen.hgvs.org",

"code": "HGVS.c:c.*1755_*1755insT",

"display": "HGVS.c:c.*1755_*1755insT"

}],

"text": "HGVS.c:c.*1755_*1755insT"

}

}, {

"url": "http://hl7.org/fhir/StructureDefinition/observation-geneticsGene",

"valueCodeableConcept": {

"coding": [{

"display": "LIPG-ACAA2"

}],

"text": "LIPG-ACAA2"

}

}, {

"url": "http://hl7.org/fhir/StructureDefinition/observation-geneticsDNASequenceVariantType",

"valueCodeableConcept": {

"coding": [{

"system": "http://vernomen.hgvs.org",

"code": "HGVS.c:n.null_nullinsT}]",

"display": "HGVS.c:n.null_nullinsT}]"

}],

"text": "HGVS.c:n.null_nullinsT}]"

}

}, {

"url": "http://hl7.org/fhir/StructureDefinition/observation-geneticsGene",

"valueCodeableConcept": {

"coding": [{

"display": "LIPG"

}],

"text": "LIPG"

}

}, {

"url": "http://hl7.org/fhir/StructureDefinition/observation-geneticsDNASequenceVariantType",

"valueCodeableConcept": {

"coding": [{

"system": "http://vernomen.hgvs.org",

"code": "HGVS.c:c.*1755A>T",

"display": "HGVS.c:c.*1755A>T"

}],

"text": "HGVS.c:c.*1755A>T"

}

}, {

"url": "http://hl7.org/fhir/StructureDefinition/observation-geneticsGene",

"valueCodeableConcept": {

"coding": [{

"display": "LIPG-ACAA2"

}],

"text": "LIPG-ACAA2"

}

}, {

"url": "http://hl7.org/fhir/StructureDefinition/observation-geneticsGene",

"valueCodeableConcept": {

"coding": [{

"display": "LIPG"

}],

"text": "LIPG"

}

}, {

"url": "http://hl7.org/fhir/StructureDefinition/observation-geneticsDNASequenceVariantType",

"valueCodeableConcept": {

"coding": [{

"system": "http://vernomen.hgvs.org",

"code": "HGVS.c:c.*1755G>T",

"display": "HGVS.c:c.*1755G>T"

}],

"text": "HGVS.c:c.*1755G>T"

}

}, {

"url": "http://hl7.org/fhir/StructureDefinition/observation-geneticsGene",

"valueCodeableConcept": {

"coding": [{

"display": "LIPG-ACAA2"

}],

"text": "LIPG-ACAA2"

}

}, {

"url": "http://hl7.org/fhir/StructureDefinition/observation-geneticsGene",

"valueCodeableConcept": {

"coding": [{

"display": "LIPG"

}],

"text": "LIPG"

}

}, {

"url": "http://hl7.org/fhir/StructureDefinition/observation-geneticsDNASequenceVariantType",

"valueCodeableConcept": {

"coding": [{

"system": "http://vernomen.hgvs.org",

"code": "HGVS.c:c.*1755A>G",

"display": "HGVS.c:c.*1755A>G"

}],

"text": "HGVS.c:c.*1755A>G"

}

}, {

"url": "http://hl7.org/fhir/StructureDefinition/observation-geneticsGene",

"valueCodeableConcept": {

"coding": [{

"display": "LIPG-ACAA2"

}],

"text": "LIPG-ACAA2"

}

}, {

"url": "http://hl7.org/fhir/StructureDefinition/observation-geneticsGene",

"valueCodeableConcept": {

"coding": [{

"display": "MC4R"

}],

"text": "MC4R"

}

}, {

"url": "http://hl7.org/fhir/StructureDefinition/observation-geneticsDNASequenceVariantType",

"valueCodeableConcept": {

"coding": [{

"system": "http://vernomen.hgvs.org",

"code": "HGVS.c:c.-420C>T",

"display": "HGVS.c:c.-420C>T"

}],

"text": "HGVS.c:c.-420C>T"

}

}, {

"url": "http://hl7.org/fhir/StructureDefinition/observation-geneticsGene",

"valueCodeableConcept": {

"coding": [{

"display": "MC4R-CDH20"

}],

"text": "MC4R-CDH20"

}

}, {

"url": "http://hl7.org/fhir/StructureDefinition/observation-geneticsGene",

"valueCodeableConcept": {

"coding": [{

"display": "MC4R"

}],

"text": "MC4R"

}

}, {

"url": "http://hl7.org/fhir/StructureDefinition/observation-geneticsDNASequenceVariantType",

"valueCodeableConcept": {

"coding": [{

"system": "http://vernomen.hgvs.org",

"code": "HGVS.c:c.-420T>A",

"display": "HGVS.c:c.-420T>A"

}],

"text": "HGVS.c:c.-420T>A"

}

}, {

"url": "http://hl7.org/fhir/StructureDefinition/observation-geneticsGene",

"valueCodeableConcept": {

"coding": [{

"display": "MC4R-CDH20"

}],

"text": "MC4R-CDH20"

}

}, {

"url": "http://hl7.org/fhir/StructureDefinition/observation-geneticsGene",

"valueCodeableConcept": {

"coding": [{

"display": "MC4R"

}],

"text": "MC4R"

}

}, {

"url": "http://hl7.org/fhir/StructureDefinition/observation-geneticsDNASequenceVariantType",

"valueCodeableConcept": {

"coding": [{

"system": "http://vernomen.hgvs.org",

"code": "HGVS.c:c.-420C>A",

"display": "HGVS.c:c.-420C>A"

}],

"text": "HGVS.c:c.-420C>A"

}

}, {

"url": "http://hl7.org/fhir/StructureDefinition/observation-geneticsGene",

"valueCodeableConcept": {

"coding": [{

"display": "MC4R-CDH20"

}],

"text": "MC4R-CDH20"

}

}, {

"url": "http://hl7.org/fhir/StructureDefinition/observation-geneticsGene",

"valueCodeableConcept": {

"coding": [{

"display": "GCKR"

}],

"text": "GCKR"

}

}, {

"url": "http://hl7.org/fhir/StructureDefinition/observation-geneticsDNASequenceVariantType",

"valueCodeableConcept": {

"coding": [{

"system": "http://vernomen.hgvs.org",

"code": "HGVS.c:c.1337T>C",

"display": "HGVS.c:c.1337T>C"

}],

"text": "HGVS.c:c.1337T>C"

}

}, {

"url": "http://hl7.org/fhir/StructureDefinition/observation-geneticsGene",

"valueCodeableConcept": {

"coding": [{

"display": "GCKR"

}],

"text": "GCKR"

}

}, {

"url": "http://hl7.org/fhir/StructureDefinition/observation-geneticsDNASequenceVariantType",

"valueCodeableConcept": {

"coding": [{

"system": "http://vernomen.hgvs.org",

"code": "HGVS.c:c.1337T>C",

"display": "HGVS.c:c.1337T>C"

}],

"text": "HGVS.c:c.1337T>C"

}

}, {

"url": "http://hl7.org/fhir/StructureDefinition/observation-geneticsGene",

"valueCodeableConcept": {

"coding": [{

"display": "GCKR"

}],

"text": "GCKR"

}

}, {

"url": "http://hl7.org/fhir/StructureDefinition/observation-geneticsDNASequenceVariantType",

"valueCodeableConcept": {

"coding": [{

"system": "http://vernomen.hgvs.org",

"code": "HGVS.c:c.1422+94T>C",

"display": "HGVS.c:c.1422+94T>C"

}],

"text": "HGVS.c:c.1422+94T>C"

}

}, {

"url": "http://hl7.org/fhir/StructureDefinition/observation-geneticsGene",

"valueCodeableConcept": {

"coding": [{

"display": "PAX1-LINC01432"

}],

"text": "PAX1-LINC01432"

}

}, {

"url": "http://hl7.org/fhir/StructureDefinition/observation-geneticsGene",

"valueCodeableConcept": {

"coding": [{

"display": "LINC01432"

}],

"text": "LINC01432"

}

}, {

"url": "http://hl7.org/fhir/StructureDefinition/observation-geneticsDNASequenceVariantType",

"valueCodeableConcept": {

"coding": [{

"system": "http://vernomen.hgvs.org",

"code": "HGVS.c:n.445+1177A>G",

"display": "HGVS.c:n.445+1177A>G"

}],

"text": "HGVS.c:n.445+1177A>G"

}

}, {

"url": "http://211.253.251.80:19080/FHIR/StructureDefinition/extension-observation-classificationVariants",

"extension": [{

"url": "pathogeny",

"valueCodeableConcept": {

"coding": [{

"system": "http://loinc.org",

"code": "53037-8",

"display": "Genetic disease sequence variation interpretation"

}],

"text": "Genetic disease sequence variation interpretation"

}

}, {

"url": "clinicalRelavance",

"valueCodeableConcept": {

"coding": [{

"system": "http://snomed.info/sct",

"code": "7882003",

"display": "Identified"

}],

"text": "Identified"

}

}]

}, {

"url": "http://211.253.251.80:19080/FHIR/StructureDefinition/extension-observation-HGVSversion",

"valueString": "15.11"

}, {

"url": "http://211.253.251.80:19080/FHIR/StructureDefinition/extension-observation-geneticsAllelicFrequency",

"valueString": "1"

}],

"status": "final",

"code": {

"coding": [{

"system": "http://loinc.org",

"code": "51969-4",

"display": "Genetic Analysis Report"

}]

},

"subject": {

"reference": "Patient/04294"

}

},

"request": {

"method": "POST",

"url": "Observation"

}

}, {

"fullUrl": "urn:uuid:919a8530-1ea0-4a00-a4ea-1b29a44814cf",

"resource": {

"resourceType": "Sequence",

"meta": {

"profile": ["http://211.253.251.80:19080/FHIR/StructureDefinition/snubh-sequence-genetics"]

},

"extension": [{

"url": "ReadDepth(DP)",

"valueString": "30"

}, {

"url": "ReferenceAllelicDepth(AD_Ref)",

"valueString": "16"

}, {

"url": "AlternativeAllelicDepth(AD_Alt)",

"valueString": "14"

}, {

"url": "AlleleFrequency(AF)",

"valueString": "0.5"

}, {

"url": "GENOTYPE",

"valueString": "T/C"

}, {

"url": "ReadDepth(DP)",

"valueString": "28"

}, {

"url": "ReferenceAllelicDepth(AD_Ref)",

"valueString": "11"

}, {

"url": "AlternativeAllelicDepth(AD_Alt)",

"valueString": "17"

}, {

"url": "AlleleFrequency(AF)",

"valueString": "0.5"

}, {

"url": "GENOTYPE",

"valueString": "T/G"

}, {

"url": "ReadDepth(DP)",

"valueString": "26"

}, {

"url": "ReferenceAllelicDepth(AD_Ref)",

"valueString": "10"

}, {

"url": "AlternativeAllelicDepth(AD_Alt)",

"valueString": "16"

}, {

"url": "AlleleFrequency(AF)",

"valueString": "0.5"

}, {

"url": "GENOTYPE",

"valueString": "G/A"

}, {

"url": "ReadDepth(DP)",

"valueString": "25"

}, {

"url": "ReferenceAllelicDepth(AD_Ref)",

"valueString": "10"

}, {

"url": "AlternativeAllelicDepth(AD_Alt)",

"valueString": "15"

}, {

"url": "AlleleFrequency(AF)",

"valueString": "0.5"

}, {

"url": "GENOTYPE",

"valueString": "T/A"

}, {

"url": "ReadDepth(DP)",

"valueString": "18"

}, {

"url": "ReferenceAllelicDepth(AD_Ref)",

"valueString": "7"

}, {

"url": "AlternativeAllelicDepth(AD_Alt)",

"valueString": "11"

}, {

"url": "AlleleFrequency(AF)",

"valueString": "0.5"

}, {

"url": "GENOTYPE",

"valueString": "CA/C"

}, {

"url": "ReadDepth(DP)",

"valueString": "31"

}, {

"url": "ReferenceAllelicDepth(AD_Ref)",

"valueString": "16"

}, {

"url": "AlternativeAllelicDepth(AD_Alt)",

"valueString": "15"

}, {

"url": "AlleleFrequency(AF)",

"valueString": "0.5"

}, {

"url": "GENOTYPE",

"valueString": "A/G"

}, {

"url": "ReadDepth(DP)",

"valueString": "33"

}, {

"url": "ReferenceAllelicDepth(AD_Ref)",

"valueString": "18"

}, {

"url": "AlternativeAllelicDepth(AD_Alt)",

"valueString": "15"

}, {

"url": "AlleleFrequency(AF)",

"valueString": "0.5"

}, {

"url": "GENOTYPE",

"valueString": "GT/G"

}, {

"url": "ReadDepth(DP)",

"valueString": "26"

}, {

"url": "ReferenceAllelicDepth(AD_Ref)",

"valueString": "11"

}, {

"url": "AlternativeAllelicDepth(AD_Alt)",

"valueString": "15"

}, {

"url": "AlleleFrequency(AF)",

"valueString": "0.5"

}, {

"url": "GENOTYPE",

"valueString": "G/C"

}, {

"url": "ReadDepth(DP)",

"valueString": "17"

}, {

"url": "ReferenceAllelicDepth(AD_Ref)",

"valueString": "10"

}, {

"url": "AlternativeAllelicDepth(AD_Alt)",

"valueString": "7"

}, {

"url": "AlleleFrequency(AF)",

"valueString": "0.5"

}, {

"url": "GENOTYPE",

"valueString": "C/CAT"

}, {

"url": "ReadDepth(DP)",

"valueString": "19"

}, {

"url": "ReferenceAllelicDepth(AD_Ref)",

"valueString": "10"

}, {

"url": "AlternativeAllelicDepth(AD_Alt)",

"valueString": "9"

}, {

"url": "AlleleFrequency(AF)",

"valueString": "0.5"

}, {

"url": "GENOTYPE",

"valueString": "A/G"

}, {

"url": "ReadDepth(DP)",

"valueString": "19"

}, {

"url": "ReferenceAllelicDepth(AD_Ref)",

"valueString": "12"

}, {

"url": "AlternativeAllelicDepth(AD_Alt)",

"valueString": "7"

}, {

"url": "AlleleFrequency(AF)",

"valueString": "0.5"

}, {

"url": "GENOTYPE",

"valueString": "A/G"

}, {

"url": "ReadDepth(DP)",

"valueString": "30"

}, {

"url": "ReferenceAllelicDepth(AD_Ref)",

"valueString": "15"

}, {

"url": "AlternativeAllelicDepth(AD_Alt)",

"valueString": "15"

}, {

"url": "AlleleFrequency(AF)",

"valueString": "0.5"

}, {

"url": "GENOTYPE",

"valueString": "C/A"

}, {

"url": "ReadDepth(DP)",

"valueString": "29"

}, {

"url": "ReferenceAllelicDepth(AD_Ref)",

"valueString": "20"

}, {

"url": "AlternativeAllelicDepth(AD_Alt)",

"valueString": "9"

}, {

"url": "AlleleFrequency(AF)",

"valueString": "0.5"

}, {

"url": "GENOTYPE",

"valueString": "T/C"

}, {

"url": "ReadDepth(DP)",

"valueString": "31"

}, {

"url": "ReferenceAllelicDepth(AD_Ref)",

"valueString": "10"

}, {

"url": "AlternativeAllelicDepth(AD_Alt)",

"valueString": "21"

}, {

"url": "AlleleFrequency(AF)",

"valueString": "0.5"

}, {

"url": "GENOTYPE",

"valueString": "T/C"

}, {

"url": "ReadDepth(DP)",

"valueString": "23"

}, {

"url": "ReferenceAllelicDepth(AD_Ref)",

"valueString": "0"

}, {

"url": "AlternativeAllelicDepth(AD_Alt)",

"valueString": "23"

}, {

"url": "AlleleFrequency(AF)",

"valueString": "1.0"

}, {

"url": "GENOTYPE",

"valueString": "GT/GT"

}, {

"url": "ReadDepth(DP)",

"valueString": "28"

}, {

"url": "ReferenceAllelicDepth(AD_Ref)",

"valueString": "0"

}, {

"url": "AlternativeAllelicDepth(AD_Alt)",

"valueString": "28"

}, {

"url": "AlleleFrequency(AF)",

"valueString": "1.0"

}, {

"url": "GENOTYPE",

"valueString": "T/T"

}, {

"url": "ReadDepth(DP)",

"valueString": "26"

}, {

"url": "ReferenceAllelicDepth(AD_Ref)",

"valueString": "0"

}, {

"url": "AlternativeAllelicDepth(AD_Alt)",

"valueString": "26"

}, {

"url": "AlleleFrequency(AF)",

"valueString": "1.0"

}, {

"url": "GENOTYPE",

"valueString": "T/T"

}, {

"url": "ReadDepth(DP)",

"valueString": "21"

}, {

"url": "ReferenceAllelicDepth(AD_Ref)",

"valueString": "0"

}, {

"url": "AlternativeAllelicDepth(AD_Alt)",

"valueString": "21"

}, {

"url": "AlleleFrequency(AF)",

"valueString": "1.0"

}, {

"url": "GENOTYPE",

"valueString": "G/G"

}, {

"url": "ReadDepth(DP)",

"valueString": "26"

}, {

"url": "ReferenceAllelicDepth(AD_Ref)",

"valueString": "13"

}, {

"url": "AlternativeAllelicDepth(AD_Alt)",

"valueString": "13"

}, {

"url": "AlleleFrequency(AF)",

"valueString": "0.5"

}, {

"url": "GENOTYPE",

"valueString": "G/A"

}, {

"url": "ReadDepth(DP)",

"valueString": "20"

}, {

"url": "ReferenceAllelicDepth(AD_Ref)",

"valueString": "0"

}, {

"url": "AlternativeAllelicDepth(AD_Alt)",

"valueString": "20"

}, {

"url": "AlleleFrequency(AF)",

"valueString": "1.0"

}, {

"url": "GENOTYPE",

"valueString": "T/T"

}, {

"url": "ReadDepth(DP)",

"valueString": "21"

}, {

"url": "ReferenceAllelicDepth(AD_Ref)",

"valueString": "10"

}, {

"url": "AlternativeAllelicDepth(AD_Alt)",

"valueString": "11"

}, {

"url": "AlleleFrequency(AF)",

"valueString": "0.5"

}, {

"url": "GENOTYPE",

"valueString": "G/T"

}, {

"url": "ReadDepth(DP)",

"valueString": "21"

}, {

"url": "ReferenceAllelicDepth(AD_Ref)",

"valueString": "0"

}, {

"url": "AlternativeAllelicDepth(AD_Alt)",

"valueString": "21"

}, {

"url": "AlleleFrequency(AF)",

"valueString": "1.0"

}, {

"url": "GENOTYPE",

"valueString": "C/C"

}, {

"url": "ReadDepth(DP)",

"valueString": "21"

}, {

"url": "ReferenceAllelicDepth(AD_Ref)",

"valueString": "0"

}, {

"url": "AlternativeAllelicDepth(AD_Alt)",

"valueString": "21"

}, {

"url": "AlleleFrequency(AF)",

"valueString": "1.0"

}, {

"url": "GENOTYPE",

"valueString": "C/C"

}, {

"url": "ReadDepth(DP)",

"valueString": "16"

}, {

"url": "ReferenceAllelicDepth(AD_Ref)",

"valueString": "0"

}, {

"url": "AlternativeAllelicDepth(AD_Alt)",

"valueString": "16"

}, {

"url": "AlleleFrequency(AF)",

"valueString": "1.0"

}, {

"url": "GENOTYPE",

"valueString": "C/C"

}, {

"url": "ReadDepth(DP)",

"valueString": "27"

}, {

"url": "ReferenceAllelicDepth(AD_Ref)",

"valueString": "0"

}, {

"url": "AlternativeAllelicDepth(AD_Alt)",

"valueString": "27"

}, {

"url": "AlleleFrequency(AF)",

"valueString": "1.0"

}, {

"url": "GENOTYPE",

"valueString": "T/T"

}, {

"url": "ReadDepth(DP)",

"valueString": "25"

}, {

"url": "ReferenceAllelicDepth(AD_Ref)",

"valueString": "15"

}, {

"url": "AlternativeAllelicDepth(AD_Alt)",

"valueString": "10"

}, {

"url": "AlleleFrequency(AF)",

"valueString": "0.5"

}, {

"url": "GENOTYPE",

"valueString": "A/G"

}, {

"url": "libraryType",

"valueString": "TruSeqDNAPCRFree(350)"

}, {

"url": "libraryName",

"valueString": "04294"

}, {

"url": "MID",

"valueString": "D707"

}, {

"url": "application",

"valueString": "WholeGenomeResequencing"

}, {

"url": "Q20Ratio",

"valueString": "96.44"

}, {

"url": "GcRatio",

"valueString": "42.26"

}, {

"url": "Q30Ratio",

"valueString": "90.15"

}, {

"url": "AlignmentTools",

"valueString": "Dragen(ver.2)"

}, {

"url": "VariantCalling",

"valueString": "GaTK(ver.4)"

}, {

"url": "SNPAnnotation",

"valueString": "SnpEff_v.4.1"

}],

"coordinateSystem": 1,

"patient": {

"reference": "Patient/04294"

},

"device": {

"reference": "urn:uuid:0e1d4e1e-885f-4bba-8432-1e50d3923330"

},

"referenceSeq": {

"strand": 0,

"windowStart": 0,

"windowEnd": 0

},

"quality": [{

"type": "snp",

"method": {

"coding": [{

"code": "targetRegionSize",

"display": "756206028"

}]

}

}],

"readCoverage": 559650144,

"repository": [{

"type": "other",

"datasetId": "CLINVAR, dbSNP_143.hg19/SnpEff_v.4.1"

}]

},

"request": {

"method": "POST",

"url": "Sequence"

}

}, {

"fullUrl": "urn:uuid:065dae06-f3c6-4f53-b1ee-cc3868ff60f6",

"resource": {

"resourceType": "Organization",

"active": true,

"name": "Macrogen"

},

"request": {

"method": "POST",

"url": "Organization"

}

}, {

"fullUrl": "urn:uuid:0e1d4e1e-885f-4bba-8432-1e50d3923330",

"resource": {

"resourceType": "Device",

"meta": {

"profile": ["http://211.253.251.80:19080/FHIR/StructureDefinition/snubh-device-genetics"]

},

"extension": [{

"url": "http://211.253.251.80:19080/FHIR/StructureDefinition/extension-device-platformType",

"valueString": "HiSeqXTen"

}, {

"url": "http://211.253.251.80:19080/FHIR/StructureDefinition/extension-device-libraryType",

"valueString": "TruSeqDNAPCRFree(350)"

}]

},

"request": {

"method": "POST",

"url": "Device"

}

}]

}
